# On the range of lattice models in high dimensions

**DOI:** 10.1007/s00440-019-00933-1

**Published:** 2019-07-06

**Authors:** Mark Holmes, Edwin Perkins

**Affiliations:** 1 School of Mathematics and Statistics, The University of Melbourne, Parkville, VIC 3010 Australia; 2 Department of Mathematics, The University of British Columbia, Vancouver, BC V6T1Z2 Canada

**Keywords:** Super-Brownian motion, One-arm probability, Lattice trees, Voter model, Oriented percolation, Contact process, 60J68, 60F17, 60K35, 82B41, 05C05

## Abstract

We investigate the scaling limit of the *range* (the set of visited vertices) for a general class of critical lattice models, starting from a single initial particle at the origin. Conditions are given on the random sets and an associated “ancestral relation” under which, conditional on longterm survival, the rescaled ranges converge weakly to the range of super-Brownian motion as random sets. These hypotheses also give precise asymptotics for the limiting behaviour of the probability of exiting a large ball, that is for the *extrinsic one-arm probability*. We show that these conditions are satisfied by the voter model in dimensions $d\ge 2$, sufficiently spread out critical oriented percolation and critical contact processes in dimensions $d>4$, and sufficiently spread out critical lattice trees in dimensions $d>8$. The latter result proves Conjecture 1.6 of van der Hofstad et al. (Ann Probab 45:278–376, 2017) and also has important consequences for the behaviour of random walks on lattice trees in high dimensions.

## Introduction

Super-Brownian motion is a measure-valued process arising as a universal scaling limit for a variety of critical lattice models above the critical dimension in statistical physics and mathematical biology. Examples include oriented percolation [44], lattice trees [22], models for competing species such as voter models [3, 8], models for spread of disease such as contact processes [42], and percolation [18], where the full result in the latter context is the subject of ongoing research (e.g., [20]). The nature of the convergence in all these contexts is that of convergence of the associated empirical processes, conditioned on long term survival and suitably rescaled, to super-Brownian motion conditioned on survival. Moreover, often only convergence of the finite-dimensional distributions is known. Extending this to convergence on path space for lattice trees was recently carried out in [39] with great effort.

Convergence of the actual random sets of occupied sites to the range of super-Brownian motion is one of the most natural questions, but has not been achieved in any of these settings (convergence at a fixed time was done for the voter model in [3], and for the simple setting of branching random walk it is implicit in [10]). We provide a unified solution to this problem in the form of quite general conditions under which the rescaled ranges of a single occupancy particle model on the integer lattice (in discrete or continuous time) converge to the range of super-Brownian motion, conditional on survival. The conditions include convergence of the associated integrated measure-valued processes to integrated super-Brownian motion, but a feature of our results is that convergence of finite-dimensional distributions suffices (see Lemma 2.2 below). We verify the conditions for the voter model in two or more dimensions, for critical oriented percolation and the critical contact process in more than 4 dimensions, and for critical lattice trees in more than eight dimensions.

As a consequence of the above convergence, we obtain the precise asymptotics for the *extrinsic one-arm probability* (i.e. the probability that the random set is not contained in the ball of radius *r*, centred at the origin). The simpler problem of establishing the asymptotics of the *intrinsic one-arm probability* (the probability that there is an occupied site at time *t*) has itself only recently been resolved at this level of generality [38]. For sufficiently spread-out (unoriented) percolation in dimensions $d>6$, Kozma and Nachmias [28] have identified the correct power law decay, but have not proved a limit theorem.

Our general lattice models include a random “ancestral relation” which in the case of random graphs such as lattice trees or oriented percolation is a fundamental part of the model, but for particle models such as the voter model or contact process, arises naturally from the graphical construction of such models. Our first main result will in fact be a uniform modulus of continuity for all “ancestral paths”. This result plays a crucial role in our proof of the above convergence, but it is also important in its own right.

We begin by briefly defining the four models which motivated our general results. These models depend on a random walk step kernel (probability mass function), $D:{\mathbb {Z}}^d\rightarrow {\mathbb {R}}_+$, with finite range and covariance matrix $\sigma ^2_{\scriptscriptstyle D}I_{d\times d}$ for some $\sigma ^2_{\scriptscriptstyle D}>0$, and such that $D(-x)=D(x)$ and $D(o)=0$, where *o* will denote the origin. By finite range we mean there is an $L\ge 1$ such that $D(x)=0$ if $\Vert x\Vert >L$ where $\Vert x\Vert$ is the $L_\infty$ norm of *x*. Let |*A*| denote the cardinality of a finite set *A*.

***The voter model***

The *voter model* on ${\mathbb {Z}}^d$ (introduced in [7, 21]) is a spin-flip system, and so in particular, a continuous time Feller process $(\xi _t)_{t\ge 0}$ with state space $\{0,1\}^{{\mathbb {Z}}^d}$ and flip rates as follows. With rate one each vertex, say at *x*, imposes its type (0 or 1) on a randomly chosen vertex *y* with probability $D(y-x)$. Let $\xi _t(x)\in \{0,1\}$ denote the type of $x\in {\mathbb {Z}}^d$ at time $t\ge 0$, and let ${\mathcal {T}}_t:=\{x \in {\mathbb {Z}}^d:\xi _t(x)=1\}$. In the notation of [30] the flip rate at site *x* in state $\xi$ is

$$\begin{aligned} c(x,\xi )=\sum _{y:\xi (y)\ne \xi (x)}D(y-x). \end{aligned}$$

If ${\mathbb {E}}[|{\mathcal {T}}_0|]<\infty$, then $|{\mathcal {T}}_t|$ is a non-negative martingale and the extinction time $S^{\scriptscriptstyle (1)}=\inf \{t\ge 0:|{\mathcal {T}}_t|=0\}$ is a.s. finite (see Lemma 7.1(b) below). We will usually assume that the process starts with a single site of type 1 at time 0, located at the origin *o*, i.e.

$$\begin{aligned} {\mathbb {P}}({\mathcal {T}}_0=\{o\})=1. \end{aligned}$$

***Oriented percolation***

For an introduction to oriented percolation (OP) see e.g. [44]. For simplicity we take *D* to be uniform on $([-L,L]^d{\setminus } \{o\})\cap {\mathbb {Z}}^d$, where $d>4$, although more general kernels are possible (see Remark 2.7). The bond $((n,x),(n+1,y))$ is occupied with probability $pD(y-x)$, where $p\in [0,\Vert D\Vert _{\infty }^{-1}]$, independent of all other bonds. Let ${\mathbb {P}}_p$ denote the law of the model. We say that there is an occupied path from (*n*, *x*) to $(n',x')$, and write $(n,x) \rightarrow (n',x')$, if there is a sequence $x=x_0,x_1,\dots , x_{n'-n}=x'$ in ${\mathbb {Z}}^d$ such that $((n+i-1,x_{i-1}),(n+i,x_{i}))$ is occupied for each $i=1,\dots , n'-n\ge 0$. We include the convention that $(n,x) \rightarrow (n,x')$ if and only if $x=x'$. Let ${\mathcal {T}}_n=\{x\in {\mathbb {Z}}^d:(0,o) \rightarrow (n,x)\}$, and observe that ${\mathbb {P}}_p({\mathcal {T}}_0=\{o\})=1$. Define $p_c=\sup \{p:\lim _{n \rightarrow \infty }{\mathbb {P}}_p(\mathcal {T}_n\ne \varnothing )=0\}$. By (1.12) of [44], $p_c=1+O(L^{-d})$, and so $p_c\in (0,\Vert D\Vert _{\infty }^{-1})$ for *L* large. Let ${\mathbb {P}}={\mathbb {P}}_{p_c}$. It is well known (e.g. [38]) that $\lim _{n \rightarrow \infty }{\mathbb {P}}(\mathcal {T}_n\ne \varnothing )=0$.

***Lattice trees***

A lattice tree (LT) *T* on ${\mathbb {Z}}^d$, is a finite connected simple graph in ${\mathbb {Z}}^d$ with no cycles. It consists of a set of lattice bonds, *E*(*T*) (unordered pairs of points in ${\mathbb {Z}}^d$), together with the corresponding set of end-vertices, *V*(*T*), in ${\mathbb {Z}}^d$. By connected we mean that for any distinct $v_1,v_2\in V(T)$ there is an $m \in {\mathbb {N}}$ and a function $w:\{0,\dots ,m\}\rightarrow V(T)$ so that $w(0)=v_1$, $w(m)=v_2$, and for all $1\le k\le m$, $\{w(k-1),w(k)\}\in E(T)$. We call *w* a path in *T* of length *m* from $v_1$ to $v_2$. Given any two vertices $v_1,v_2$ in the tree, the lack of cycles means there is a unique path (length 0 if $v_1=v_2$) of bonds connecting $v_1$ and $v_2$. The number of such bonds, $d_T(v_1,v_2)$, is the tree distance between $v_1$ and $v_2$. It is a metric on the set of vertices, called the tree metric. Let ${\mathbb {T}}_L(x)$ denote the countable space of lattice trees on ${\mathbb {Z}}^d$ whose vertex set contains $x\in {\mathbb {Z}}^d$ and for which every bond has $L_\infty$-norm at most $L\ge 1$. The parameter *L* will be taken sufficiently large for our main results. We now describe a way of choosing a “random” lattice tree $\mathcal {T}$ containing the origin, i.e. $\mathcal {T}\in {\mathbb {T}}_L:={\mathbb {T}}_L(o)$.

Let $d>8$ and let $D(\cdot )$ be the probability mass function of the uniform distribution on a finite box $([-L,L]^d{\setminus } o)\cap {\mathbb {Z}}^d$ (see Remark 2.7). For $e=(y,x)$, let $D(e):=D(x-y)$. For a lattice tree $T\in {\mathbb {T}}_L(x)$ define

1.1 $$\begin{aligned} W_{z,D}(T)=z^{|T|}\prod _{e\in E(T)}D(e), \end{aligned}$$

where |*T*| is the number of edges in *T*. Since *D* is uniform (1.1) could also be written as $(c_{\scriptscriptstyle L}z)^{|T|}$. For any $z>0$ such that $\rho _z:=\sum _{T\in {\mathbb {T}}_L(o)}W_{z,D}(T)<\infty$ we can define a probability on ${\mathbb {T}}_L(o)$ by ${\mathbb {P}}_z({\mathcal {T}}=T)=\rho _z^{-1}W_{z,D}(T)$. Sub-additivity arguments show that $z_{\scriptscriptstyle D}=\sup \{z>0:\rho _z<\infty \}\in (0,\infty )$. It is known (e.g. Theorem 1.2 of [17]) that $\rho _{z_{\scriptscriptstyle D}}<\infty$ and that $z_{\scriptscriptstyle D}=\sup \{z:{\mathbb {E}}_{z}[|{\mathcal {T}}|]<\infty \}$, but ${\mathbb {E}}_{z_{\scriptscriptstyle D}}[|{\mathcal {T}}|]=\infty$. Hereafter we write $W(\cdot )$ for the critical weighting $W_{z_{\scriptscriptstyle D},D}(\cdot )$, write $\rho :=\rho _{z_{\scriptscriptstyle D}}$ and ${\mathbb {P}}={\mathbb {P}}_{z_{\scriptscriptstyle D}}$, and we select a random tree ${\mathcal {T}}$ according to this critical weighting.

For $T\in {\mathbb {T}}_L(o)$ and $m \in {\mathbb {Z}}_+$, let $T_{m}$ denote the set of vertices in *T* of tree distance *m* from *o*, so ${\mathcal {T}}_m$ is the corresponding set of vertices for our random tree ${\mathcal {T}}$ and ${\mathbb {P}}({\mathcal {T}}_0=\{o\})=1$. Note that $\lim _{n \rightarrow \infty }{\mathbb {P}}(\mathcal {T}_n\ne \varnothing )=0$ (see e.g. [38]).

***Contact process***

The contact process (CP) on ${\mathbb {Z}}^d$ is a spin-flip system $(\xi _t)_{t\ge 0}$ (hence a cadlag Feller process) with state space $\{0,1\}^{{\mathbb {Z}}^d}$ and rates ($\lambda >0$)

$$\begin{aligned} c(x,\xi )=\xi (x)+(1-\xi (x))\lambda \sum _y D(y-x)\xi (y). \end{aligned}$$

It describes the spread of an infection in that $\xi _t(x)=1$ if and only if site *x* is infected at time *t*, and the above rates imply that an infected site *x* recovers at rate 1, and an uninfected site *x* with rate $\lambda$ chooses a “neighbour” *y* with probability $D(y-x)$ and becomes infected if *y* is infected. For simplicity (see Remark 2.7) we will take *D* to be the probability mass function of the uniform distribution on $([-L,L]^d{\setminus }\{o\})\cap {\mathbb {Z}}^d$ where $d>4$. We start from a single infected particle at the origin at time 0. Let ${\mathbb {P}}_{\lambda }$ denote a probability measure under which $(\xi _t)_{t\ge 0}$ has the law above with rates $\lambda$. Let $\mathcal {T}_t=\{x:\xi _t(x)=1\}$, so ${\mathbb {P}}_\lambda (\mathcal {T}_0=\{o\})=1$. Let $\lambda _c=\sup \{\lambda :\lim _{t \rightarrow \infty }{\mathbb {P}}_\lambda (\mathcal {T}_t\ne \varnothing )=0\}$, and ${\mathbb {P}}={\mathbb {P}}_{\lambda _c}$. It is known (e.g. [38, 41]) that $\lambda _c\in (0,\infty )$, and that $\lim _{t \rightarrow \infty }{\mathbb {P}}(\mathcal {T}_t\ne \varnothing )=0$.

***General models and ancestral relations***

Our goal is to establish general conditions for convergence of the ranges of a wide class of rescaled lattice models (including the voter model, oriented percolation, lattice trees, the contact process, and perhaps also percolation). We introduce our general framework in this section. The time index *I* will either be ${\mathbb {Z}}_+$ (discrete time) or $[0,\infty )$ (continuous time). We use the notation $I_t=\{s \in I:s\le t\}$. As we will be dealing with random compact sets, we let $\mathcal {K}$ denote the set of compact subsets of ${\mathbb {R}}^d$. We equip it with the Hausdorff metric $d_0$ (and note that $(\mathcal {K},d_0)$ is Polish) defined by $d_0(\varnothing ,K)=d_0(K,\varnothing )=1$ for $K\ne \varnothing$, while for $K,K'\ne \varnothing$

1.2 $$\begin{aligned} d_0(K,K')&=d_1(K,K')\wedge 1, \quad \text { where } \nonumber \\ d_1(K,K')&:=\varDelta _1(K,K')+\varDelta _1(K',K), \nonumber \\ \varDelta _1(K,K')&:=\inf \big \{\delta >0:K\subset \{x:d(x,K')\le \delta \}\big \},\quad \text { and } \nonumber \\ d(x,K)&:=\inf \{|x-y|:y \in K\}. \end{aligned}$$

As our models of interest will be single occupancy models, we assume throughout that

1.3 $$\begin{aligned}&\varvec{{\mathcal {T}}}=({\mathcal {T}}_t)_{t\in I} \text { is a stochastic process taking values in the finite} \nonumber \\&\text {subsets of }{\mathbb {Z}}^d\text { such that }{\mathcal {T}}_0=\{o\}, \text {and in continuous time the}\nonumber \\&\text {sample paths are cadlag }\mathcal {K}-\text {valued.} \end{aligned}$$

**Notation.** For a metric space *M*, $\mathcal {D}([0,\infty ),M)$ will denote the space of cadlag *M*-valued paths with the Skorokhod topology, and $C_b(M)$ is the space of bounded ${\mathbb {R}}$-valued continuous functions on *M*. $C^2_b({\mathbb {R}}^d)$ is the set of bounded continuous functions whose first and second order partials are also in $C_b({\mathbb {R}}^d)$. Integration of *f* with respect to a measure $\mu$ is often denoted by $\mu (f)$.

Cadlag paths are bounded on bounded intervals and so this implies

1.4 $$\begin{aligned} \text {for any }t\in I, \cup _{s\in I_t}{\mathcal {T}}_s\text { is a finite subset of }{\mathbb {Z}}^d. \end{aligned}$$

We will write

$$\begin{aligned} (t,x)\in {\mathcal {\varvec{T}}}\text { if and only if }x\in {\mathcal {T}}_t,\text { where }(t,x)\in I\times {\mathbb {Z}}^d. \end{aligned}$$

$(\mathcal {F}_t)_{t\in I}$ will denote a filtration with respect to which $({\mathcal {T}}_t)_{t \in I}$ is adapted. In practice it may be larger than the filtration generated by $\varvec{{\mathcal {T}}}$.

A random ancestral relation, $(s,y)\overset{\varvec{a}}{\rightarrow }(t,x)$, on $I\times {\mathbb {Z}}^d$ will be fundamental to our analysis. If it holds we say that (*s*, *y*) is an ancestor of (*t*, *x*), and it will imply $s\le t$. We write

$$\begin{aligned} (s_1,y_1)\overset{\varvec{a}}{\rightarrow }\cdots \overset{\varvec{a}}{\rightarrow }(s_N,y_N)\text { iff }(s_i,y_i)\overset{\varvec{a}}{\rightarrow }(s_{i+1},y_{i+1})\text { for }i=1,\dots ,N-1, \end{aligned}$$

and define (for $s,t\in I$, $x,y\in {\mathbb {Z}}^d$),

1.5 $$\begin{aligned} e_{s,t}(y,x)={\left\{ \begin{array}{ll}\mathbb {1}((s,y)\overset{\varvec{a}}{\rightarrow }(t,x))&{}\text { if }s< t\\ \mathbb {1}(x=y\in {\mathcal {T}}_t)&{}\text { if }s\ge t, \end{array}\right. } \text { and }{{\hat{e}}}_t(y,x)(s)=e_{s,t}(y,x). \end{aligned}$$

We will assume $\overset{\varvec{a}}{\rightarrow }$ satisfies the following conditions where (AR)(i)–(iii) will hold off a single null set which we usually ignore:

1.6 $$\begin{aligned} \mathbf{(AR) } (i)&\quad \text {For all }(s,y),(t,x)\in I\times {\mathbb {Z}}^d: \nonumber \\&\quad (s,y)\overset{\varvec{a}}{\rightarrow }(s,x)\text { iff }x=y\in {\mathcal {T}}_s, \end{aligned}$$

1.7 $$\begin{aligned}&\quad (s,y)\overset{\varvec{a}}{\rightarrow }(t,x) \text { implies } s\le t, y\in {\mathcal {T}}_s,\text { and }x\in {\mathcal {T}}_t, \end{aligned}$$

1.8 $$\begin{aligned}&\quad (0,o)\overset{\varvec{a}}{\rightarrow }(t,x)\text { iff }x\in {\mathcal {T}}_t. \end{aligned}$$

1.9 $$\begin{aligned} (ii)&\quad \text {For any }0\le s_1<s_2<s_3\text { in }I \text { and }y_1,y_2,y_3\in {\mathbb {Z}}^d: \nonumber \\&\quad (s_1,y_1)\overset{\varvec{a}}{\rightarrow }(s_2,y_2)\overset{\varvec{a}}{\rightarrow }(s_3,y_3) \text { implies}\ (s_1,y_1)\overset{\varvec{a}}{\rightarrow }(s_3,y_3). \end{aligned}$$

1.10 $$\begin{aligned}&\quad \text {Conversely if }(s_1,y_1)\overset{\varvec{a}}{\rightarrow }(s_3,y_3)\text { then }\exists y_{2}\in {\mathcal {T}}_{s_2} \text { s.t. } \end{aligned}$$

1.11 $$\begin{aligned}&\quad (s_1,y_1)\overset{\varvec{a}}{\rightarrow }(s_2,y_2)\overset{\varvec{a}}{\rightarrow }(s_3,y_3). \nonumber \\ (iii)&\quad \text {If } I=[0,\infty ),\text { then for every }x,y\in {\mathbb {Z}}^d: \end{aligned}$$

1.12 $$\begin{aligned}&\quad {{\hat{e}}}_t(y,x)\in \mathcal {D}([0,\infty ),{\mathbb {R}})=:\mathcal {D}_{{\mathbb {R}}}, \text { for every }t \in I, \text { and } \nonumber \\&\quad t\mapsto {{\hat{e}}}_t(y,x)\in \mathcal {D}([0,\infty ),\mathcal {D}_{{\mathbb {R}}}). \nonumber \\ (iv)&\quad e_{s,t}(y,x)\text { is }\mathcal {F}_t-\text {measurable for all }s, t\text { in }I \text { and }x,y\in {\mathbb {Z}}^d. \end{aligned}$$

We call $\overset{\varvec{a}}{\rightarrow }$ an ancestral relation iff (AR) holds. In this case we call $(\varvec{{\mathcal {T}}},\overset{\varvec{a}}{\rightarrow })$ an ancestral system.

### Remark 1.1

1. It is immediate from (1.8) and (1.10) (the latter with $(s_1,s_2,s_3)=(0,s,t)$) that
  1.13 $$\begin{aligned} {\mathcal {T}}_s=\varnothing \Rightarrow {\mathcal {T}}_t=\varnothing \ \forall t\ge s. \end{aligned}$$
2. In practice it is often easiest to verify (AR)(iii) by showing $s\mapsto e_{s,t}(y,x)$ is cadlag for each *t*, *x*, *y*, and that
  1.14 $$\begin{aligned}&\text {For each }t\ge 0,x,y\in {\mathbb {Z}}^d\text { there is a }\delta >0\text { s.t. } \nonumber \\&{{\hat{e}}}_u(y,x)={{\hat{e}}}_t(y,x),\ \forall u\in [t,t+\delta )\ \text { and } \end{aligned}$$
  1.15 $$\begin{aligned}&{{\hat{e}}}_u(y,x)={{\hat{e}}}_{u'}(y,x),\ \forall u,u'\in (t-\delta ,t)\cap [0,\infty ). \end{aligned}$$

**We will always assume **(1.3) **and (AR) when dealing with our abstract models.**

In the discrete time case we can extend ${\mathcal {T}}_t$ and $\mathcal {F}_t$ to $t\in [0,\infty )$ by

$$\begin{aligned} {\mathcal {T}}_t={\mathcal {T}}_{\lfloor t\rfloor },\quad {\mathcal {\varvec{T}}}=\{(t,x):x\in {\mathcal {T}}_t,t\ge 0\},\quad \mathcal {F}_t=\mathcal {F}_{\lfloor t\rfloor }, \end{aligned}$$

and define $(s,y)\overset{\varvec{a}}{\rightarrow }(t,x)$ for all $t\ge s\ge 0$ by

1.16 $$\begin{aligned} (s,y)\overset{\varvec{a}}{\rightarrow }(t,x)\text { iff }(\lfloor s \rfloor ,y)\overset{\varvec{a}}{\rightarrow }(\lfloor t \rfloor ,x). \end{aligned}$$

It is easy to check then that in the discrete case (AR)(i)–(iv) hold, where now $s,s_i,t$ are allowed to take values in $[0,\infty )$. Moreover (1.14) and (1.15) hold.

**Note: We allow***n***to denote a real parameter in**$[1,\infty )$.

For $A\subset {\mathbb {R}}^d$ and $a\in {\mathbb {R}}$ define $aA=\{ax:x \in A\}$. To rescale our model for $n\in [1,\infty )$ we set

$$\begin{aligned} {\mathcal {T}}^{\scriptscriptstyle {(n)}}_t={\mathcal {T}}_{nt}/\sqrt{n},\text { for }t\ge 0, \end{aligned}$$

and for $$s,t\ge 0$$, $$x,y\in {\mathbb {Z}}^d/\sqrt{n}$$

$$\begin{aligned} \text {we write }(s,y)\overset{\varvec{a,n}}{\rightarrow }(t,x)\text { iff }(ns,\sqrt{n} y)\overset{\varvec{a}}{\rightarrow }(nt,\sqrt{n} x). \end{aligned}$$

We also define for $n\in [1,\infty )$, $s,t\ge 0$ and $x,y\in {\mathbb {Z}}^d/\sqrt{n}$,

1.17 $$\begin{aligned} {{\hat{e}}}^{\scriptscriptstyle {(n)}}_t(y,x)(s)=e^{\scriptscriptstyle {(n)}}_{s,t}(y,x)=e_{ns,nt}(\sqrt{n} y,\sqrt{n} x), \end{aligned}$$

and note that ${{\hat{e}}}^{\scriptscriptstyle {(n)}}_t(y,x)(s)={{\hat{e}}}_{nt}(\sqrt{n} y,\sqrt{n} x)(ns)$.

Here is a simple consequence of (AR)(ii) which will be used frequently.

### Lemma 1.2

W.p.1 if $n\in [1,\infty )$, $M\in {\mathbb {N}}$, $0\le s_0<s_1<\dots <s_M$, and $(s_0,y_0)\overset{\varvec{a,n}}{\rightarrow }(s_M,y_M)$, then there are $y_1\in {\mathcal {T}}^{\scriptscriptstyle {(n)}}_{s_1},\dots , y_{M-1}\in {\mathcal {T}}^{\scriptscriptstyle {(n)}}_{s_{M-1}}$ s.t. $(s_{i-1},y_{i-1})\overset{\varvec{a,n}}{\rightarrow }(s_i,y_i)$ for $i=1,\dots ,M$.

### Proof

Fix $\omega$ s.t. (AR)(i)–(iii) hold. By scaling we may assume $n=1$. By (1.10) there is a $y_1\in {\mathcal {T}}_{s_1}$ s.t. $(s_0,y_0)\overset{\varvec{a}}{\rightarrow }(s_1,y_1)\overset{\varvec{a}}{\rightarrow }(s_M,y_M)$. Repeat this argument $M-2$ times to construct the required sequence. $\square$

### Definition 1.3

An *ancestral path* to $(t,x)\in {\mathcal {\varvec{T}}}$ is a cadlag path $w=(w_s)_{s \ge 0}$ for which $(s,w_s)\overset{\varvec{a}}{\rightarrow }(s',w_{s'})$ for every $0\le s\le s'\le t$, and $w_s=x$ for all $s\ge t$. The random collection of all ancestral paths to points in ${\mathcal {\varvec{T}}}$ is denoted by $\mathcal {W}$ and is called the system of ancestral paths for $(\varvec{{\mathcal {T}}},\overset{\varvec{a}}{\rightarrow })$.

If $n\in [1,\infty )$ and *w* is an ancestral path to $(nt,\sqrt{n} x)$, we define the rescaled ancestral path $w^{\scriptscriptstyle {(n)}}$ by $w^{\scriptscriptstyle {(n)}}_s=w_{ns}/\sqrt{n}$, and call $w^{\scriptscriptstyle {(n)}}$ an ancestral path to $(t,x)\in {\mathcal {T}}^{\scriptscriptstyle {(n)}}_t$.

### Remark 1.4

It is easy to check that if $I={\mathbb {Z}}_+$ then (1.16) and (1.6) imply that for any ancestral path $w\in \mathcal {W}$, $w_s=w_{\lfloor s \rfloor }$ for all $s\ge 0$. For this reason we will often restrict our ancestral paths to $s\in {\mathbb {Z}}_+$.

### Proposition 1.5

With probability 1 for any $(t,x)\in {\mathcal {\varvec{T}}}$, $\mathcal {W}$ includes at least one ancestral path to (*t*, *x*).

The elementary proof is given in Sect. 6 below.

Let us briefly consider (1.3) and (AR) for our prototype models. For lattice trees (1.3) is immediate. Since a lattice tree $T\in {\mathbb {T}}_L$ is a tree, for any $x\in T_{m}$ there is a unique “ancestral” path $w(m,x)=(w_k(m,x))_{k\le m}$ of length *m* in the tree from *o* to *x*. Moreover $w_k(m,x)\in T_k$ for all $0\le k\le m$. Define $(k,y)\overset{\varvec{a}}{\rightarrow }(m,x)$ iff $x\in {\mathcal {T}}_m$, $0\le k\le m$, and $w_k(m,x)=y$. Here we allow $m=0$. AR(i) and AR(ii) are then elementary to verify. It remains to verify AR(iv) which is deferred to Sect. 9 where the definition of $\mathcal {F}_t$ is also given.

For the voter model there will be a unique ancestral path *w*(*t*, *x*) for each $(t,x)\in {\mathcal {\varvec{T}}}$ (see Lemma 7.3), obtained by tracing back the opinion 1 at *x* at time *t* to its source at the origin at time 0. Formally the ancestral paths are defined by reversing the dual system of coalescing random walks, obtained from the graphical construction of the voter model. We then define $(s,y)\overset{\varvec{a}}{\rightarrow }(t,x)$ iff $0\le s\le t$, $x\in {\mathcal {T}}_t$ and $w_s(t,x)=y$. This standard construction is described in Sect. 7 where (AR) and (1.3) are then verified (see Lemmas 7.1 and 7.2).

For oriented percolation (1.3) is immediate, and we write $(n,x) \overset{\varvec{a}}{\rightarrow }(n',x')$ if $(0,o) \rightarrow (n,x)$ and $(n,x) \rightarrow (n',x')$. Here ancestral paths are not unique, since there may be many occupied paths between (*n*, *x*) and $(n',x')$. Let $\mathcal {F}_n$ denote the $\sigma$-field generated by the bond occupation status for all bonds $((m-1,x),(m,x'))$ for $1\le m\le n$ and $x,x'\in {\mathbb {Z}}^d$. It is easy to see that $\overset{\varvec{a}}{\rightarrow }$ is an ancestral relation (i.e. (AR) holds).

For the contact process, the ancestral relation will be similar to that for oriented percolation, but will be obtained from the graphical construction of the contact process (see Sect. 10 below).

***Survival probability and measure-valued processes***

The survival time of our scaled model ${\mathcal {T}}^{\scriptscriptstyle {(n)}}$ is

$$\begin{aligned} S^{\scriptscriptstyle {(n)}}=\inf \{t\ge 0:{\mathcal {T}}^{\scriptscriptstyle {(n)}}_t=\varnothing \}, \end{aligned}$$

so that by (1.13) and (1.3),

1.18 $$\begin{aligned} {\mathcal {T}}^{\scriptscriptstyle {(n)}}_t=\varnothing \text { for all }t\ge S^{\scriptscriptstyle {(n)}}. \end{aligned}$$

The unscaled survival time is $S^{\scriptscriptstyle (1)}$, and for $t>0$, the unscaled survival probability is defined as

$$\begin{aligned} \theta (t):={\mathbb {P}}(S^{\scriptscriptstyle (1)}>t). \end{aligned}$$

Our main results require a number of conditions on ${\varvec{{\mathcal {T}}}}$, the first of which concerns the asymptotics of the survival probability.

**Notation.** Write $f(t)\sim g(t)$ as $t\uparrow \infty$ iff $\lim _{t\rightarrow \infty }f(t)/g(t)=1$. Similarly for $f(t)\sim g(t)$ as $t\downarrow 0$.

### Condition 1

*(Survival Probability)* There is a constant $s_D>0$ and a non-decreasing function $m:[0,\infty )\rightarrow (0,\infty )$ such that $m(t)\uparrow \infty$, as $t\uparrow \infty$

1.19 $$\begin{aligned}&\theta (t)\sim \frac{s_D}{m(t)}\text { as }t\uparrow \infty , \end{aligned}$$

1.20 $$\begin{aligned}&\text {for any }s>0,\ \lim _{t\rightarrow \infty }m(st)/m(t)=s, \end{aligned}$$

and a constant $c_{1.21}\ge 1$ such that

1.21 $$\begin{aligned} \frac{m(sn)}{m(n)}\le c_{1.21}s, \, \forall s,n\ge 1,\quad \frac{m(n)}{m(sn)}\le c_{1.21}\frac{1}{s}, \, \text { for }1\le s\le n. \end{aligned}$$

The monotonicity properties of *m* and $\theta$ and (1.19) easily show that

1.22 $$\begin{aligned} 0<{\underline{s}}_D=\inf _{t\ge 0}m(t)\theta (t)\le \sup _{t\ge 0}m(t)\theta (t)={\overline{s}}_D<\infty , \end{aligned}$$

Note also that the first inequality in (1.21) with $n=1$ implies that

1.23 $$\begin{aligned} m(s)\le c_{1.23}s, \text { for all } s\ge 1. \end{aligned}$$

For oriented percolation with $d>4$ we set

1.24 $$\begin{aligned} m(t)=m^{OP}(t)=A^2V(t\vee 1), \end{aligned}$$

where $A,V>0$ are constants that depend on *D*;

1.25 $$\begin{aligned} A=\lim _{t\rightarrow \infty }{\mathbb {E}}[|{\mathcal {T}}_t|], \end{aligned}$$

and *V* is called the vertex factor (see [38] and in particular Condition 1.1 with $r=2$ and $\mathbf {k}=\mathbf {0}$ for the former). Similarly we have such an *A* and *V* for the contact process with $d>4$ and for lattice trees with $d>8$ and we set

1.26 $$\begin{aligned} m(t)&=m^{CP}(t)=A^2V(t\vee 1), \text { and } \end{aligned}$$

1.27 $$\begin{aligned} m(t)&=m^{LT}(t)=A^2V(t\vee 1). \end{aligned}$$

For the voter model in two or more dimensions we set

$$\begin{aligned} m(t)=m^{VM}(t)={\left\{ \begin{array}{ll} t\vee 1 &{}\text { if }d>2\\ \frac{t\vee e}{\log (t\vee e)}&{}\text { if }d=2, \end{array}\right. } \end{aligned}$$

and

$$\begin{aligned} 0<\beta _D={\left\{ \begin{array}{ll}P_o(S_n\ne o\ \ \forall n\in {\mathbb {N}})&{}\text { if }d>2\\ 2\pi \sigma ^2_{\scriptscriptstyle D}&{}\text { if } d=2. \end{array}\right. } \end{aligned}$$

In the above under $P_o$, $(S_n)_{n \in {\mathbb {Z}}_+}$ is a discrete-time random walk with step distribution *D*, started at *o*, and $\sigma ^2_{\scriptscriptstyle D}I_{d\times d}$ is the covariance matrix of *D*.

### Proposition 1.6

- Condition 1 holds for critical sufficiently spread out oriented percolation in dimension $d>4$ with $s_D=2A$.
- Condition 1 holds for critical sufficiently spread out lattice trees in dimension $d>8$ with $s_D=2A$.
- Condition 1 holds for critical sufficiently spread out contact processes in dimension $d>4$ with $s_D=2A$.
- Condition 1 holds for the voter model in dimension $d>1$ with $s_D=\beta _D^{-1}$.

### Proof

Conditions (1.20) and (1.21) are obvious in all four cases.

(a),(b),(c) For all 3 models (1.19) is a special case of Theorem 1.4 of [38] (for oriented percolation this was first proved in [36, 37]).

(d) Theorem $1^\prime$ of [4] (or (1.5) of [3]) gives (1.19) for the voter model. $\square$

We can reinterpret the state of our rescaled models in terms of an empirical measure given by

$$\begin{aligned} X^{\scriptscriptstyle {(n)}}_t=\frac{1}{m(n)}\sum _{x\in {\mathcal {T}}^{\scriptscriptstyle {(n)}}_t}\delta _x=\frac{1}{m(n)}\sum _{x\in {\mathcal {T}}_{nt}}\delta _{x/\sqrt{n}}. \end{aligned}$$

So $X_t^{\scriptscriptstyle {(n)}}$ takes values in the Polish space $\mathcal {M}_F({\mathbb {R}}^d)$ of finite measures on ${\mathbb {R}}^d$ equipped with the topology of weak convergence. It follows from (1.3) that the measure-valued process $\varvec{X}^{\scriptscriptstyle {(n)}}=(X_t^{\scriptscriptstyle {(n)}})_{t\ge 0}$ is in the Polish space $\mathcal {D}:=\mathcal {D}([0,\infty ),\mathcal {M}_F({\mathbb {R}}^d))$. We define the survival map $\mathcal {S}:\mathcal {D} \rightarrow [0,\infty )$ for $\varvec{\nu }=(\nu _t)_{t\ge 0}\in \mathcal {D}$ by

$$\begin{aligned} \mathcal {S}(\varvec{\nu })=\inf \{t>0:\nu _t({\mathbb {R}}^d)=0\}, \end{aligned}$$

so that our survival times satisfy $S^{\scriptscriptstyle {(n)}}=\mathcal {S}(\varvec{X}^{\scriptscriptstyle {(n)}})$, and $X^{\scriptscriptstyle {(n)}}_t=0$ for all $t\ge S^{\scriptscriptstyle {(n)}}$ by (1.18).

***Weak convergence and super-Brownian motion***

An adapted a.s. continuous $\mathcal {M}_F({\mathbb {R}}^d)$-valued process, $\varvec{X}=(X_s)_{s\ge 0}$, on a complete filtered probability space $(\varOmega ,\mathcal {F},\mathcal {F}_t,{\mathbb {P}}_{X_0})$ is said to be a super-Brownian motion (SBM) with branching rate $\gamma >0$ and diffusion parameter $\sigma _0^2>0$ (or a $(\gamma , \sigma _0^2)$-SBM) starting at $X_0\in \mathcal {M}_F({\mathbb {R}}^d)$ iff it solves the following martingale problem (see [32, Section II.5] for well-posedness of this martingale problem):

$$\begin{aligned}&\forall \phi \in C_b^2({\mathbb {R}}^d), M_t(\phi )=X_t(\phi )-X_0(\phi )-\int _0^t X_s( \sigma _0^2\varDelta \phi /2)\,ds\\&\text {is a continuous }\mathcal {F}_t-\text {martingale starting at }0, \text { and with square} \\&\text {function }\langle M(\phi )\rangle _t=\int _0^tX_s(\gamma \phi ^2)ds. \end{aligned}$$

Let $S=\mathcal {S}(\varvec{X})$. Associated with such a SBM is a $\sigma$-finite measure, ${\mathbb {N}}_o={\mathbb {N}}_o^{\gamma , \sigma _0^2}$, on the space of continuous measure-valued paths satisfying $\nu _0=0$, $0<S<\infty$ and $\nu _s=0$ for all $s\ge S$; let $\varOmega ^{Ex}_C$ denote the space of such paths. ${\mathbb {N}}_o$ is called the canonical measure for super-Brownian motion. The connection between ${\mathbb {N}}_o$ and super-Brownian motion is that if $\varXi$ is a Poisson point process on the space $\varOmega _C^{Ex}$ with intensity ${\mathbb {N}}_o$, then

1.28 $$\begin{aligned} X_t=\int \nu _t\,d\varXi (\varvec{\nu }), t>0;\quad X_0=\delta _0 \end{aligned}$$

defines a SBM starting at $\delta _0$. It is known that

1.29 $$\begin{aligned} {\mathbb {N}}_o(S>s)=\frac{2}{\gamma s}<\infty \text { for all s>0}. \end{aligned}$$

Intuitively ${\mathbb {N}}_o$ governs the evolution of the descendants of a single ancestor at time zero, starting from the origin. For the above and more information on the canonical measure of super-Brownian motion see, e.g., Section II.7 of [32]. We will sometimes work with the unconditioned measures ($n\in [1,\infty )$)

$$\begin{aligned} \mu _n(\cdot )=m(n){\mathbb {P}}(\cdot ). \end{aligned}$$

Note that (1.19) and (1.20) of Condition 1 together imply

1.30 $$\begin{aligned} \text {for each }s>0,\quad \lim _{n\rightarrow \infty }\frac{2}{ s_D}\mu _n(S^{\scriptscriptstyle {(n)}}>s)=\frac{2}{ s}. \end{aligned}$$

Combining (1.22) with (1.21) and taking limits from the left, we arrive at

1.31 $$\begin{aligned} \frac{{\underline{s}}_D}{c_{1.21}(s\vee 1)}\le \mu _n(S^{\scriptscriptstyle {(n)}}>s)\le \mu _n(S^{\scriptscriptstyle {(n)}}\ge s)\le \frac{c_{1.21}{\overline{s}}_D}{s\wedge n},\quad \forall s\ge 0. \end{aligned}$$

Suppressing dependence on $\gamma , \sigma _0^2$, for $s>0$ we define probabilities by

1.32 $$\begin{aligned} {\mathbb {P}}_n^{s}(\cdot )={\mathbb {P}}\big ( \cdot \big | S^{\scriptscriptstyle {(n)}}>s) \end{aligned}$$

and

1.33 $$\begin{aligned} {\mathbb {N}}_o^s(\cdot )={\mathbb {N}}_o(\cdot \,|\, S>s). \end{aligned}$$

We slightly abuse the above notation and will also denote super-Brownian motion under ${\mathbb {N}}_o$, or the probabilities ${\mathbb {N}}_o^s$, by $\varvec{X}=(X_t)_{t\ge 0}$. Then for $\phi _k(x):=e^{ik \cdot x}$ we have

1.34 $$\begin{aligned} {\mathbb {N}}_o[X_t(\phi _k)]=e^{-\sigma _0^2|k|^2t/2}. \end{aligned}$$

For LT, OP, and CP and *d* large as usual, we will use the fact that our rescaled models converge (at least in the sense of finite-dimensional distributions) to SBM with $\gamma =1$ and

1.35 $$\begin{aligned} \sigma _0^2= \sigma ^2_{\scriptscriptstyle D}v=\frac{\sigma ^2}{d}v, \end{aligned}$$

where $\sigma ^2(=d \sigma ^2_{\scriptscriptstyle D})$ and *v* are as in [22, 42, 44], and the model dependent constant $v>0$ is non-trivial (it involves so-called lace expansion coefficients). *v* satisfies (see e.g. [40, page 295] with $p^*=2$ for LT and $p^*=1$ for OP,CP)

1.36 $$\begin{aligned} v=1+O(L^{-d/p^*}). \end{aligned}$$

For the VM and LT, the above convergence holds on path space:

### Proposition 1.7

Consider either critical lattice trees with $d>8$ and *L* sufficiently large, or the voter model with $d>1$. Then for any $s>0$,

$$\begin{aligned} {\mathbb {P}}_n^{s}(\varvec{X}^{\scriptscriptstyle {(n)}}\in \cdot ) \overset{w}{\longrightarrow }{\mathbb {N}}_o^s, \quad (\text {weak convergence on }\mathcal {D}), \end{aligned}$$

where ${\mathbb {N}}_o^s$ has parameters $(\gamma , \sigma _0^2)=(1, \sigma ^2_{\scriptscriptstyle D}v)$ for lattice trees, and $(\gamma , \sigma _0^2)=(2\beta _D, \sigma ^2_{\scriptscriptstyle D})$ for the voter model.

### Proof

For the voter model this is Theorem 4(b) of [3]. For lattice trees this is an immediate consequence of Theorem 1.2 of [39] (and an elementary rescaling) and (1.30). The reader should note, however, that the definition of $\mu _n$ in [39] and that given above differ by a constant factor of *A*. $\square$

### Remark 1.8

The size of our random tree, $|\mathcal {T}|$, is random. If we were to condition on $|\mathcal {T}|=n$ then (since all trees with *n* edges have equal weight) our conditional probability measure chooses a tree with *n* edges uniformly at random. If one rescales the $n+1$ vertex locations by $D_1^{-1}n^{-1/4}$ (where $D_1$ is a constant depending on *L*) and considers the random mass distribution obtained by assigning mass $(n+1)^{-1}$ to each vertex, Aldous (Section 4.2 of [1]) had conjectured, and Derbez and Slade [11, 34] then proved, that the resulting rescaled empirical measures converges weakly to Integrated Super-Brownian Excursion (ISE). The latter is essentially super-Brownian motion integrated over time and conditioned to have total mass one.

For oriented percolation and the contact process, convergence of *the finite-dimensional distributions* (f.d.d.’s) to those of a $(1, \sigma ^2_{\scriptscriptstyle D}v)$-SBM, where *v* is as in (1.36), is known [42, 44] (see also [24, 38]) but tightness (and convergence on path space) remains open. The actual weak convergence result we will impose on our lattice models (Condition 6 below) will in fact follow from convergence of the f.d.d.’s and a moment bound on the total mass (see Lemma 2.2).

Our main objective is to give general conditions for the convergence of the rescaled sets of occupied sites. This convergence follows neither from the notions of weak convergence above, nor from the weak convergence of the so-called historical processes (see e.g. [9, 23]).

***Range***

The range of $\mathbf{\mathcal {T}}$ is $R^{\scriptscriptstyle (1)}=\cup _{t\in I}{\mathcal {T}}_t$, which by (1.4) and (1.13) is a finite subset of ${\mathbb {Z}}^d$ on $\{S^{\scriptscriptstyle (1)}<\infty \}$, and hence under Condition 1 will be finite a.s. The range of ${\mathcal {T}}^{\scriptscriptstyle {(n)}}$ is $R^{\scriptscriptstyle {(n)}}=R^{\scriptscriptstyle (1)}/\sqrt{n}=\cup _{t\ge 0}{\mathcal {T}}^{\scriptscriptstyle {(n)}}_t$. So by the above we see that

1.37 $$\begin{aligned} \text {Condition}~1 \text { implies }R^{\scriptscriptstyle {(n)}} \text { is a.s. a finite subset of }{\mathbb {R}}^d. \end{aligned}$$

Let $\mathcal {R}:\mathcal {D} \rightarrow$ closed subsets of ${\mathbb {R}}^d$ be defined by

$$\begin{aligned} \mathcal {R}(\varvec{\nu })=\text {supp}\left( \int _0^\infty \nu _t \,dt\right) , \end{aligned}$$

where ${\mathrm {supp}}(\mu )$ is the closed support of a measure $\mu$. Clearly $R^{\scriptscriptstyle {(n)}}=\mathcal {R}(\varvec{X}^{\scriptscriptstyle {(n)}})$ for all $n\ge 1$. The radius mapping $r_0: \mathcal {K}\rightarrow [0,\infty )$ on the space of compact subsets of ${\mathbb {R}}^d$ is given by

$$\begin{aligned} r_0(K)=\sup \{|x|:x\in K\}. \end{aligned}$$

Of particular interest is the *extrinsic one-arm probability*

$$\begin{aligned} \eta _r={\mathbb {P}}\big (R^{\scriptscriptstyle (1)}\cap \overline{B(o,r)}^c\ne \varnothing \big )={\mathbb {P}}(r_0(R^{\scriptscriptstyle (1)})>r). \end{aligned}$$

In the setting of high-dimensional critical percolation, Kozma and Nachmias [28] have proved that as $r\rightarrow \infty$, $r^2\eta _r$ is bounded above and below by positive constants. It is believed (see e.g. [19, Open Problem 11.2] and [39, Conjecture 1.6]) that in fact $r^2\eta _r\rightarrow C>0$ for various critical models (percolation, voter, lattice trees, oriented percolation, and the contact process) all above their respective critical dimensions. To understand this $r^{-2}$ behaviour in terms of the above weak convergence results, consider the one-arm probabilities for the limiting super-Brownian motion.

The range of a $(\gamma , \sigma _0^2)$-SBM ${\varvec{X}}$ is denoted by

$$\begin{aligned} R=\mathcal {R}(\varvec{X}). \end{aligned}$$

The a.e. continuity of $(X_t)_{t\ge 0}$ easily shows that

$$\begin{aligned} R=\overline{\cup _{t\ge 0}\, \text {supp}(X_t)}\quad {\mathbb {N}}_o-\text {a.e.} \end{aligned}$$

We note that *R* is a compact subset ${\mathbb {N}}_o$-a.e. This is well-known under ${\mathbb {P}}_{\delta _0}$ (see, e.g. Corollary III.1.4 of [32]) and then follows easily under ${\mathbb {N}}_o$ using (1.28). We now state a quantitative version of this from [27]. For $d\ge 1$, let $v_d:B_d(0,1)\rightarrow {\mathbb {R}}_+$ be the unique positive radial solution of

1.38 $$\begin{aligned} \varDelta v_d=v_d^2, \qquad \text { with }\lim _{|x|\uparrow 1}v_d(x)=+\infty . \end{aligned}$$

(See Theorem 1 of [27] for existence and uniqueness of $v_d$.)

### Lemma 1.9

For all $d\ge 1$ and $r>0$, ${\mathbb {N}}_o^{\gamma , \sigma _0^2}(r_0(R)>r)=\frac{v_d(0) \sigma _0^2}{\gamma }r^{-2}$.

### Proof

Theorem 1 of [27] and a simple scaling argument show that

$$\begin{aligned} {\mathbb {P}}_{\delta _0}(r_0(R)>r)=1-\exp \Bigl (-\frac{v_d(0) \sigma _0^2}{\gamma }r^{-2}\Bigr ). \end{aligned}$$

On the other hand, the left-hand side of the above is $1-\exp (-{\mathbb {N}}_o(r_0(R)>r))$ by (1.28). Combining these equalities completes the proof. $\square$

## Statement of main results

We continue to state the general conditions which will imply our main results. Recall our standing assumptions (1.3) and (AR), the function *m* from (1.21), and the unconditioned measures ($n\ge 1$) $\mu _n(\cdot )=m(n){\mathbb {P}}(\cdot )$. Recall also that $(\mathcal {F}_t)_{t\in I}$ is the filtration introduced prior to (AR) which will contain the filtration generated by $({\mathcal {T}}_s)_{s\le t}$ or equivalently by $(X^{\scriptscriptstyle (1)}_s)_{s \le t}$.

### **Conditions**

We now introduce additional conditions on $(\varvec{{\mathcal {T}}},\overset{\varvec{a}}{\rightarrow })$. Condition 2 is simple for the voter model, and is one of the outputs of the inductive approach to the lace expansion [40, 43] for other models, while Condition 3 will usually follow from Condition 1 and a form of the Markov property or Markov inequality.

#### Condition 2

*(Total Mass)*$$\quad \sup _{t\in I}{\mathbb {E}}\big [|{\mathcal {T}}_t|\big ]=c_{3}<\infty $$.

#### Condition 3

*(Conditional Survival Probability)* There exists $c_{3}>0$ such that for all $s\ge 0,t>0$, on the event $\{y \in {\mathcal {T}}_s\}$ we have (recall the function $m(\cdot )$ from (1.21))

$$\begin{aligned} {\mathbb {P}}\big (\exists z : (s,y) \overset{\varvec{a}}{\rightarrow }(s+t,z) \big |\mathcal {F}_s\big )\le \frac{c_{3}}{m(t)} \qquad \text { a.s.} \end{aligned}$$

The next condition is the main input for our uniform modulus of continuity for ancestral paths (e.g. Theorem 1 below).

#### Condition 4

*(Spatial Increments)* There exists a $p>4$ and $c_{4}=c_{4}(p)>0$ such that for every $0<s\le t$,

2.1 $$\begin{aligned} {\mathbb {E}}\left[ \sum _{x\in {\mathcal {T}}_t}\sum _{y\in {\mathcal {T}}_{t-s}}\mathbb {1}((t-s,y)\overset{\varvec{a}}{\rightarrow }(t,x))|x-y|^p\right] \le c_{4} (s\vee 1)^{p/2}. \end{aligned}$$

We will need an additional hypothesis to control the ancestral paths just before the terminal value.

#### Condition 5

*(Local Jumps)* There are $\kappa >4$ and $c_{5}>0$ such that for all $s\ge 0$, $y\in {\mathbb {Z}}^d$, and $N>0$,

2.2 $$\begin{aligned}&{\mathbb {P}}(\exists \ (t,x)\text { s.t. } (s,y)\overset{\varvec{a}}{\rightarrow }(t,x),\ t\in [s,s+2],\ |y-x|\ge N|\mathcal {F}_s) \nonumber \\&\quad \le c_{5}N^{-\kappa }\text { on }\{y\in {\mathcal {T}}_s\}. \end{aligned}$$

#### Remark 2.1

In discrete time if for some $L>0$,

2.3 $$\begin{aligned} \forall k\in {\mathbb {Z}}_+,\ \forall x,y\in {\mathbb {Z}}^d\ \ [(k,x)\overset{\varvec{a}}{\rightarrow }(k+1,y)\Rightarrow \Vert x-y\Vert \le L], \end{aligned}$$

then Condition 5 holds for any $\kappa >4$. This is obvious since the conditional probability on the left-hand side of (2.2) is then zero if $N>2\sqrt{d} L$.

If $\varvec{\nu }=(\nu _t)_{t\ge 0}\in \mathcal {D}$ and $r>0$, define ${\bar{\nu }}_{r}\in M_F({\mathbb {R}}^d)$ by ${\bar{\nu }}_r(\cdot )=\int _0^r \nu _t(\cdot ) {\mathrm {d}}t$, and $\bar{\nu }^{}_\infty \in M_F({\mathbb {R}}^d)$ by $\bar{\nu }^{}_\infty (\cdot )=\mathbb {1}(\mathcal {S}(\nu )<\infty )\int _0^\infty \nu _t(\cdot ) {\mathrm {d}}t$.

#### Condition 6

*(Measure Convergence)* There are parameter values $(\gamma , \sigma _0^2)$ for ${\mathbb {N}}_o$ so that for every $s>0$ and $0\le t_0<t_1<\infty$, as $n \rightarrow \infty$,

$$\begin{aligned} {\mathbb {P}}_n^{s}\left( {\bar{X}}^{\scriptscriptstyle {(n)}}_{t_1}-{\bar{X}}^{\scriptscriptstyle {(n)}}_{t_0}\in \cdot \right) \overset{w}{\longrightarrow }{\mathbb {N}}_o^{s }\left( {\bar{X}}_{t_1}-{\bar{X}}_{t_0}\in \cdot \right) \text { on }\mathcal {M}_F({\mathbb {R}}^d). \end{aligned}$$

For critical lattice trees ($d>8$) with *L* sufficiently large, and voter models ($d\ge 2$) the above is immediate from Proposition 1.7 with the parameter values therein. For applications to other models (including oriented percolation and the contact process) it is worth noting that convergence of finite-dimensional distributions and boundedness of arbitrary moments of the total mass suffice. The hypotheses of the next lemma will be verified for OP and CP in Sects. 8 and 10, respectively.

#### Lemma 2.2

Fix $s>0$ and let ${\mathbb {P}}_n^s$ and ${\mathbb {N}}_o^s$ be as in (1.32) and (1.33), respectively. Assume that for all $p \in {\mathbb {N}}$:

2.4 $$\begin{aligned}&\text {for all }0<u_1,\dots ,u_p<\infty , \text { as }n\rightarrow \infty , \nonumber \\&{\mathbb {P}}_n^s((X^{\scriptscriptstyle {(n)}}_{u_1},\dots ,X^{\scriptscriptstyle {(n)}}_{u_p})\in \cdot )\overset{w}{\longrightarrow }{\mathbb {N}}_0^s((X_{u_1},\dots ,X_{u_p})\in \cdot )\text { on }\mathcal {M}_F({\mathbb {R}}^d)^p, \end{aligned}$$

2.5 $$\begin{aligned}&\text {and for } t^*>0,\quad \sup _{n\ge 1}\sup _{t\le t^*}{\mathbb {E}}^s_n\left[ X_t^{\scriptscriptstyle {(n)}}(1)^p\right] :=C_{2.2}(s,t^*,p)<\infty . \end{aligned}$$

Then Condition 6 holds.

The proof is an easy Fubini argument and is given in Sect. 6.

The final condition is needed to ensure the rescaled ranges of $\varvec{{\mathcal {T}}}$ converge weakly to the range of super-Brownian motion. Together with uniform control of the ancestral paths, it will ensure that any occupied regions will be close to regions of positive integrated mass of the limiting super-Brownian motion.

#### Condition 7

*(Low Density Inequality)* There exists $c_{7}>0$ such that for any $t\ge 0$, $M>0$, and $\varDelta \ge 4$,

2.6 $$\begin{aligned}&{\mathbb {E}}\left[ \sum _{x\in {\mathcal {T}}_t}\mathbb {1}\Bigl (\exists x' \text { s.t. }(t,x)\overset{\varvec{a}}{\rightarrow }(t+\varDelta ,x'), \int _{t+\varDelta }^{t+2\varDelta }|\{y:(t,x)\overset{\varvec{a}}{\rightarrow }(s,y)\}|\,ds\le M\Bigr )\right] \nonumber \\&\le c_{7}{\mathbb {P}}\Bigg (S^{\scriptscriptstyle (1)}>\varDelta ,\ \int _{\varDelta +2}^{2\varDelta -2}|{\mathcal {T}}_s|ds\le M\Bigg ). \end{aligned}$$

Here is a condition which implies the above and is more user-friendly in discrete time. The elementary proof is given in Sect. 6.

#### Lemma 2.3

Assume the discrete time setting and there is a $c_{7}$ such that for all $\ell \in {\mathbb {Z}}_+$, $m\in {\mathbb {N}}^{\ge 4}$ and $M>0$,

2.7 $$\begin{aligned}&{\mathbb {E}}\left[ \sum _{x\in {\mathcal {T}}_\ell }\mathbb {1}(\exists x'\text { s.t. }(\ell ,x)\overset{\varvec{a}}{\rightarrow }(\ell +m,x')\text { and } \right. \nonumber \\&\left. \qquad |\{(i,y):(\ell ,x)\overset{\varvec{a}}{\rightarrow }(i,y), \ell +m+2\le i\le \ell +2m-1\}|\le M)\right] \nonumber \\&\quad \le c_{7}{\mathbb {P}}\Bigg (S^{\scriptscriptstyle (1)}>m,\ \sum _{i=m+2}^{2m-1}|{\mathcal {T}}_i|\le M\Bigg ). \end{aligned}$$

Then Condition 7 holds.

Note that Condition 4 for $s=t$ gives bounds on the spatial moments $\sum _x|x|^p{\mathbb {P}}(x\in {\mathcal {T}}_t)$. In fact a subset of the above conditions (including Condition 4) give exact asymptotics on these moments as $t\rightarrow \infty$:

#### Proposition 2.4

Assume Condition 1 and that the conclusion of Condition 4 holds for $s=t$ and all $p>4$. Assume also that (2.4) holds for $p=1$ and (2.5) holds for $p=2$. If *Z* denotes a *d*-dimensional standard normal random vector, then for all $p>0$,

2.8 $$\begin{aligned} \lim _{t\rightarrow \infty }\frac{\sum _x|x|^p{\mathbb {P}}(x\in {\mathcal {T}}_t)}{t^{p/2}}=\frac{s_D\gamma \sigma _0^{p}}{2}E[|Z|^p]. \end{aligned}$$

The easy proof will be given in Sect. 6. Such exact asymptotics were established for OP ($d>4$) in [6] under more general conditions on *D*.

### Main results

We start with a uniform modulus of continuity for the system of ancestral paths in either discrete or continuous time. As was noted before Condition 7, this modulus plays an important role in the convergence of the rescaled ranges but is also of independent interest. For critical branching Brownian motion such a modulus was first given in Theorem 4.7 of [10]. Although we assume Condition 1 for convenience, in fact the proof only requires the existence of a non-decreasing function *m* satisfying (1.21) and Condition 3, as well as Conditions 2, 4, and 5 but not the exact asymptotics in (1.19) or (1.20). We will often assume

2.9 $$\begin{aligned} \alpha \in (0,1/2), \ \beta \in (0,1]\text { satisfy }\frac{1-2\alpha }{1+\beta }\ge \frac{4}{p}, \end{aligned}$$

where *p* is as in Condition 4. We will also sometimes assume

2.10 $$\begin{aligned} 0<\alpha <\frac{1}{2}-\frac{2}{\kappa }, \end{aligned}$$

where $\kappa$ is as in Condition 5. Note that such $\alpha ,\beta$ exist since $\kappa ,p>4$.

#### Theorem 1

Assume Conditions 1 to 5 for $I={\mathbb {Z}}_+$ or ${\mathbb {R}}_+$, and $\alpha ,\beta$ satisfy (2.9) and (2.10). Set $q=\frac{\kappa (1/2-\alpha )-2}{2}\wedge 1\in (0,1]$. There is a constant $C_{1}\ge 1$ and for all $n\ge 1$, a random variable $\delta _n\in [0,1]$, such that if

2.11 $$\begin{aligned} |s_2-s_1|\le \delta _n \text { and }(s_1,y_1)\overset{\varvec{a,n}}{\rightarrow }(s_2,y_2), \end{aligned}$$

then

2.12 $$\begin{aligned} |y_1-y_2|\le C_{1}[|s_2-s_1|^\alpha +n^{-\alpha }. \end{aligned}$$

Moreover, $$\delta _n$$ satisfies

2.13 $$\begin{aligned} \mu _n(\delta _n\le \rho )\le C_{1}[\rho ^\beta +n^{-q}],\quad \forall \rho \in [0,1). \end{aligned}$$

In the discrete time setting recall that ${\mathbb {Z}}_+/n=\{j/n:j\in {\mathbb {Z}}_+\}$ is the natural time line. In this setting we can get a cleaner statement if we also assume the stronger (2.3) in place of Condition 5.

#### Theorem 1′

Assume Conditions 1 to 4 and (2.3), where $I={\mathbb {Z}}_+$. Assume also that $\alpha ,\beta$ are as in (2.9). There is a constant $C_{1'}$ and for all $n\ge 1$ a random variable $\delta _n\in (0,1]$ such that if

2.14 $$\begin{aligned} s_1,s_2\in {\mathbb {Z}}_+/n, \quad |s_2-s_1|\le \delta _n, \quad \text { and }\quad (s_1,y_1)\overset{\varvec{a,n}}{\rightarrow }(s_2,y_2), \end{aligned}$$

then

2.15 $$\begin{aligned} |y_1-y_2|\le C_{1'}|s_2-s_1|^{\alpha }, \end{aligned}$$

and $$\delta _n$$ satisfies

2.16 $$\begin{aligned} \mu _n(\delta _n\le \rho )\le C_{1'}\rho ^{\beta },\quad \forall \rho \in [0,1). \end{aligned}$$

Moreover if $s_1,s_2, y_1,y_2$ are as in (2.14) but with $s_i\in {\mathbb {R}}_+$, then (2.12) holds.

Theorems 1 and $1^\prime$ can be reinterpreted as (uniform) moduli of continuity for all ancestral paths.

#### Definition 2.5

For $\alpha ,\beta >0$, the system of ancestral paths $\mathcal {W}$ for $(\varvec{{\mathcal {T}}},\overset{\varvec{a}}{\rightarrow })$ is said to satisfy an $(\alpha ,\beta )$-modulus of continuity if there exists a random function $\delta :[1,\infty )\rightarrow [0,1]$, a function $\varepsilon :[1,\infty )\rightarrow [0,\infty )$ such that $\lim _{n\rightarrow \infty }\varepsilon (n)=0$ and a constant $c>0$ such that for any $n\in [1,\infty )$:

For every ancestral path $w\in \mathcal {W}$, and all $0\le s_1<s_2$,

$$\begin{aligned} (1)&\ |s_2-s_1|\le \delta _n\Rightarrow |w^{\scriptscriptstyle {(n)}}_{s_2}- w^{\scriptscriptstyle {(n)}}_{s_1}|\le c(|s_2-s_1|^\alpha + n^{-\alpha }).\\ (2)&\ m(n){\mathbb {P}}(\delta _n\le \rho )\le c \rho ^{\beta } + \varepsilon (n)\text { for each }\rho \in [0,1). \end{aligned}$$

#### Corollary 1

Assume Conditions 1 to 5 for $I={\mathbb {Z}}_+$ or ${\mathbb {R}}_+$, then for $\alpha ,\beta ,q$ as in Theorem 1, $\mathcal {W}$ satisfies an $(\alpha ,\beta )$-modulus of continuity with $\varepsilon (n)=C_{1}n^{-q}$.

#### Proof

If *w* is an ancestral path to $(nt,\sqrt{n} x)$ and $0\le s_1<s_2$, then for $s_i\le t$, $(s_1,w^{\scriptscriptstyle {(n)}}_{s_1})\overset{\varvec{a,n}}{\rightarrow }(s_2,w^{\scriptscriptstyle {(n)}}_{s_2})$. (1) and (2) of Definition 2.5 now follow immediately from Theorem 1 with $\delta _n$ as in the theorem and $\varepsilon (n)$ as claimed. In general the result follows because $w^{\scriptscriptstyle {(n)}}_s=w^{\scriptscriptstyle {(n)}}_{s\wedge t}$. $\square$

#### Corollary 1′

Assume Conditions 1 to 4 and (2.3), where $({\mathcal {T}}_t)_{t\in {\mathbb {Z}}_+}$ is in discrete time, and let $\alpha ,\beta$ and $\delta _n$ be as in Theorem $1^\prime$. Then for any $n\ge 1$ and $w\in \mathcal {W}$,

$$\begin{aligned} s_i\in {\mathbb {Z}}_+/n, |s_2-s_1|\le \delta _n\quad \text { implies }\quad |w^{\scriptscriptstyle {(n)}}_{s_2}-w^{\scriptscriptstyle {(n)}}_{s_1}|\le C_{1}[|s_2-s_1|^\alpha ]. \end{aligned}$$

If $s_i$ are as above but now in ${\mathbb {R}}_+$, then $|w^{\scriptscriptstyle {(n)}}_{s_2}-w^{\scriptscriptstyle {(n)}}_{s_1}|\le C_{1}[|s_2-s_1|^\alpha +n^{-\alpha }]$.

#### Proof

As above but now use Theorem $1^\prime$ in place of Theorem 1. $\square$

We will use this modulus of continuity as a tool for proving weak convergence of the range, however the result is useful more generally. For example, it provides a means to check tightness of the spatial component of the model in the extended Gromov–Hausdorff–Prohorov toplogy, cf. [2, Lemma 4.3]. For LT in particular, this has implications for random walk on LT (see e.g. [2]).

Our second main result is that, conditional on longterm survival, the rescaled range converges weakly to the range of (conditioned) SBM.

#### Theorem 2

(Convergence of the range) Assume Conditions 1–7, and let ${\mathbb {N}}_o$ be the canonical measure with parameters $(\gamma , \sigma _0^2)$ from Condition 6. Then for every $s>0$,

$$\begin{aligned} {\mathbb {P}}(R^{\scriptscriptstyle {(n)}}\in \cdot |S^{\scriptscriptstyle {(n)}}>s) \overset{w}{\longrightarrow }{\mathbb {N}}_o(R\in \cdot |S>s)\text { as }n\rightarrow \infty ,\ \ n\in [1,\infty ), \end{aligned}$$

as probability measures on $\mathcal {K}$ equipped with the Hausdorff metric.

With Lemma 2.2 in mind it is perhaps a bit surprising that such a result could be proved without a formal tightness condition. It is Theorem 1 that effectively gives tightness of the approximating ranges.

Recall that $v_d:B_d(0,1)\rightarrow {\mathbb {R}}_+$ is the unique solution of (1.38). The next result uses both Theorems 1 and 2 to give exact leading asymptotics for the extrinsic one-arm probability.

#### Theorem 3

(One-arm asymptotics) Assume Conditions 1–7. Then

2.17 $$\begin{aligned} \lim _{r\rightarrow \infty } \frac{{\mathbb {P}}(r_0(R^{\scriptscriptstyle (1)})>r)}{{\mathbb {P}}(S^{\scriptscriptstyle (1)}>r^2)}=\frac{ \sigma _0^2}{2} v_d(0), \end{aligned}$$

and so (Condition 1)

2.18 $$\begin{aligned} \lim _{r\rightarrow \infty }m(r^2){\mathbb {P}}(r_0(R^{\scriptscriptstyle (1)})>r)=\frac{ \sigma _0^2}{2} s_Dv_d(0). \end{aligned}$$

#### Remark 2.6

The proof in Sect. 5 only uses Condition 1 and the conclusions of Theorems 1 and 2.

We finally show that all of the above conditions are satisfied by the voter model ($d\ge 2$), OP ($d>4$), LT ($d>8$), and the contact process ($d>4$).

#### Theorem 4

(Voter model) For the voter model, Conditions 1–7 hold in dimensions $d\ge 2$ for any $p>4$ in Condition 4, any $\kappa >4$ in Condition 5, and $(\gamma , \sigma _0^2)=(2\beta _D, \sigma ^2_{\scriptscriptstyle D})$ in Condition 6. Hence for $d\ge 2$, if ${\mathbb {N}}_o$ is the canonical measure of SBM with parameters $(2\beta _D, \sigma ^2_{\scriptscriptstyle D})$, then

For any $\alpha \in (0,1/2)$, the system of ancestral paths, $\mathcal {W}$, satisfies an $(\alpha ,1)$-modulus of continuity with $\varepsilon (n)=C_{1}n^{-1}$ for some $C_1\ge 1$,
${\mathbb {P}}_n^{s}(R^{\scriptscriptstyle (n)}\in \cdot )\overset{w}{\longrightarrow }{\mathbb {N}}_o^{s}(R\in \cdot )$ in $\mathcal {K}$, as $n\rightarrow \infty$, for every $s>0$,
(i) $$\lim _{r\rightarrow \infty }r^2{\mathbb {P}}(r_0(R^{\scriptscriptstyle (1)})>r)=\frac{ \sigma ^2_{\scriptscriptstyle D}v_d(0)}{2\beta _D}$$ if $$d>2$$,
  (ii) $$\lim _{r\rightarrow \infty }\frac{r^2}{\log r}{\mathbb {P}}(r_0(R^{\scriptscriptstyle (1)})>r)=v_2(0)(2\pi )^{-1}$$ if $$d=2$$.

Part (v1) will give a uniform modulus of continuity for all of the rescaled dual coalescing random walks between 1’s in a voter model conditioned on longterm survival. This is stated and proved in Sect. 7 (Corollary 7.4).

In the next three results *v* is the model-dependent constant satisfying (1.36).

#### Theorem 5

(Oriented percolation) For critical OP with $d>4$ and *L* sufficiently large, Conditions 1–7 hold for any $p>4$ in Condition 4, any $\kappa >4$ in Condition 5, and $(\gamma , \sigma _0^2)=(1, \sigma ^2_{\scriptscriptstyle D}v)$ in Condition 6. Hence for $d>4$, if ${\mathbb {N}}_o$ is the canonical measure of SBM with parameters $(1, \sigma ^2_{\scriptscriptstyle D}v)$, then

1. For $\alpha \in (0,1/2)$ the system of ancestral paths, $\mathcal {W}$, satisfies an $(\alpha ,1)$-modulus of continuity with $\varepsilon (n)\equiv 0$,
2. ${\mathbb {P}}_n^{s}(R^{\scriptscriptstyle {(n)}}\in \cdot )\overset{w}{\longrightarrow }{\mathbb {N}}_o^{s}(R\in \cdot )$ in $\mathcal {K}$, as $n\rightarrow \infty$, for every $s>0$,
3. $$\lim _{r\rightarrow \infty }r^2{\mathbb {P}}(r_0(R^{\scriptscriptstyle (1)})>r)=\frac{ \sigma ^2_{\scriptscriptstyle D}vv_d(0)}{AV}$$.

#### Theorem 6

(Lattice trees) For critical lattice trees with $d>8$ and *L* sufficiently large, Conditions 1–7 hold for any $p>4$ in Condition 4, any $\kappa >4$ in Condition 5, and $(\gamma , \sigma _0^2)=(1, \sigma ^2_{\scriptscriptstyle D}v)$ in Condition 6. Hence for $d>8$, if ${\mathbb {N}}_o$ is the canonical measure of SBM with parameters $(1, \sigma ^2_{\scriptscriptstyle D}v)$, then

1. For $\alpha \in (0,1/2)$ the system of ancestral paths, $\mathcal {W}$, satisfies an $(\alpha ,1)$-modulus of continuity with $\varepsilon (n)\equiv 0$,
2. ${\mathbb {P}}_n^{s}(R^{\scriptscriptstyle {(n)}}\in \cdot )\overset{w}{\longrightarrow }{\mathbb {N}}_o^{s}(R\in \cdot )$ in $\mathcal {K}$, as $n\rightarrow \infty$, for every $s>0$,
3. $$\lim _{r\rightarrow \infty }r^2{\mathbb {P}}(r_0(R^{\scriptscriptstyle (1)})>r)=\frac{ \sigma ^2_{\scriptscriptstyle D}vv_d(0)}{AV}$$.

#### Theorem 7

(Contact process) For the critical contact process with $d>4$ and *L* large enough, Conditions 1–7 hold for any $p>4$ in Condition 4, any $\kappa >4$ in Condition 5, and $(\gamma , \sigma _0^2)=(1, \sigma ^2_{\scriptscriptstyle D}v)$ in Condition 6. Hence for $d>4$, if ${\mathbb {N}}_o$ is the canonical measure of SBM with parameters $(1, \sigma ^2_{\scriptscriptstyle D}v)$, then

1. For any $\alpha \in (0,1/2)$, the system of ancestral paths, $\mathcal {W}$, satisfies an $(\alpha ,1)$-modulus of continuity with $\varepsilon (n)=C_{{1}}n^{-1}$ for some $C_1\ge 1$,
2. ${\mathbb {P}}_n^{s}(R^{\scriptscriptstyle (n)}\in \cdot )\overset{w}{\longrightarrow }{\mathbb {N}}_o^{s}(R\in \cdot )$ in $\mathcal {K}$, as $n\rightarrow \infty$, for every $s>0$,
3. $$\lim _{r\rightarrow \infty }r^2{\mathbb {P}}(r_0(R^{\scriptscriptstyle (1)})>r)=\frac{ \sigma ^2_{\scriptscriptstyle D}vv_d(0)}{AV}$$.

#### Remark 2.7

Although we have assumed *D* is uniform on $[-L,L]^d{\setminus }\{o\}$ for OP, LT and CP, the results (and proofs) for these models also hold for more general kernels. Namely, if $h:{\mathbb {R}}^d\rightarrow [0,\infty )$ is bounded, continuous almost everywhere, supported on $[-1,1]^d$, invariant under the symmetries of ${\mathbb {Z}}^d$ and such that $\int h(x)dx =1$, then for $L\ge 1$ we may define *D* by

$$\begin{aligned} D(x)={\left\{ \begin{array}{ll}\frac{h(x/L)}{\sum _{y\in {\mathbb {Z}}^d{\setminus }\{0\}}h(y/L)}&{} \text { if }x\ne 0,\\ 0&{}\text { if } x=0.\end{array}\right. } \end{aligned}$$

The case $h=2^{-d}1_{[-1,1]^d}$ gives the uniform distribution. This is simply a matter of noting that the references we provide for checking these conditions hold for this class of more general *D*’s, and that our own arguments never make use of the uniform nature of *D*.

#### Remark 2.8

In Sects. 8–10 the hypotheses of Lemma 2.2 will be verified for LT, OP and the CP (for the dimensions noted in the above theorems). As Conditions 1-7 also hold by the above, we see in particular that Proposition 2.4 applies for all of these models. Recalling from the above that $s_D=2A$, $\gamma =1$ and $\sigma _0^2= \sigma ^2_{\scriptscriptstyle D}v$ for all of these models, Proposition 2.4 gives

2.19 $$\begin{aligned} \lim _{t\rightarrow \infty }\frac{\sum _x|x|^p{\mathbb {P}}(x\in {\mathcal {T}}_t)}{t^{p/2}}=A( \sigma ^2_{\scriptscriptstyle D}v)^{p/2}E[|Z|^{p}]. \end{aligned}$$

Recall that for lattice trees $w_k(m,x)$ is the location of the ancestor of $x\in {\mathcal {T}}_m$ in generation $k\le m$ of the tree. We can also apply Corollary $1^\prime$ to obtain a modulus of continuity for the large scale behaviour of $k\mapsto w_k(m,x)$ conditional on longterm survival; see Corollary 9.1 in Sect. 9.

Note that the lower bounds on ${\mathbb {P}}(r_0(R^{(1)})>r)$ in (v3) and (t3) above follow easily from Proposition 1.7 and the lower semi-continuity of the support map on $\mathcal {M}_F({\mathbb {R}}^d)$ (see Lemma 4.3 below). So the importance of (v3) and (t3) in these cases are the matching upper bounds.

For the voter model, in an interesting article Merle [31] has studied the probability that a distant site *x* ever holds the opinion 1 (i.e. ${\mathbb {P}}(\xi _t(x)=1 \text { for some }t\ge 0)$ in the limit as $|x| \rightarrow \infty$). Although clearly related to the behaviour of the range of the voter model, and in particular (v3) above, neither result implies the other.

### Discussion on conditions and extensions

Note that, although the above list of conditions may appear lengthy, we shall see that for our prototype models, all of the conditions are either already proved in the literature, or are fairly easy to establish from known results, although Condition 4 was in some cases proved very recently in response to this work. For the voter model this Condition is elementary for any $d\ge 2$ and any $p>4$; see Sect. 7. For our prototype models, a Markov (or sub-Markov) argument reduces Condition 4 to the $s=t$ case, which is a bound of the form

2.20 $$\begin{aligned} \sum _x |x|^p {\mathbb {P}}(x \in \mathcal {T}_t)\le C_p(t\vee 1)^{(p/2)}, \quad \forall t>0. \end{aligned}$$

For the voter model such a reduction is implicit in (7.18), while for OP, the CP, and LT the reductions are given by Lemma 8.2, Lemma 10.2 and Lemma 9.5 respectively, with $f(x)=|x|^p$. For OP, (2.20) was established by Chen and Sakai [6] (see Theorem 1.2 of [6] and the ensuing comment there to allow $\alpha =\infty$) where in fact exact asymptotics are given for large *t*. In response to our main results, Sakai and Slade [33] established (2.20) for sufficiently spread-out critical CP ($d>4$) and LT ($d>8$) for any $p>4$ (their method also applies to OP). For LT, their result extends the result for $p=6$ that appeared in an earlier version of this paper [25] (and their method is simpler). Thus the earlier version [25] has been superseded by this paper and [33]. Note that (2.19) shows that their upper bounds then immediately give exact asymptotics as $t\rightarrow \infty$.

Recent work [20] suggests that it may be of interest to consider these results in the setting of critical sufficiently spread out percolation ($d>6$). In this setting, for example, Theorem 3 would refine a result of Kozma and Nachmias [28], by giving a bona fide limit for the one-arm probability. However we quickly point out that there is much work still to be done here. For example, the important Condition 6 has yet to be verified in this setting (see [18] for partial results). In a very interesting paper Tim Hulshof [26] has shown that there is a phase transition in the one-arm exponents which corresponds to the condition $p>4$ in Condition 4, both for critical percolation ($d>6$) and critical branching random walk (BRW) $(d>2)$. He works with infinite range kernels *D* satisfying $D(x)\approx |x|^{-d-\alpha }$ as |*x*| becomes large (where $f(x)\approx g(x)$ means $cg\le f\le Cg$ for some $0<c\le C<\infty$) and shows that if $R^{\scriptscriptstyle (1)}$ is the open cluster of the origin (resp. set of vertices visited by BRW), then

$$\begin{aligned} {\mathbb {P}}(r_0(R^{\scriptscriptstyle (1)})>r)\approx r^{-\min (4,\alpha )/2} \text{ as } r\rightarrow \infty . \end{aligned}$$

For $\alpha \ge 4$ this gives the one-arm exponent 2, found for the finite-range setting in [28], but for $\alpha <4$ the one-arm exponent is no longer 2. It is easy to check that $\alpha >4$ implies that $\sum _z|z|^p D(z)<\infty$ for some $p>4$. Although we have assumed single occupancy, our proof is modelled after the proof for branching Brownian motion, which is implicit in [10], and minor modifications to our arguments will give similar results for branching random walk. (One needs to define (AR) for individual particles at each site, for example by using the arboreal labelling in [10]). Hulshof’s results, described above, strongly suggest that the restriction $p>4$ in Condition 4 is sharp. In fact we conjecture that Theorems 1–3 will continue to hold without the finite range assumption on *D*, but will all fail if we then allow $p<4$ in Condition 4 for our four prototype models with such “long-range” kernels.

The remainder of this paper is organised as follows. In Sect. 3 we establish the moduli of continuity, i.e., Theorems 1 and $1^\prime$. In Sect.  4 we prove our general result on convergence of the rescaled ranges, Theorem 2. In Sect.  5 both of the above ingredients are used to prove the one-arm result, Theorem 3. Lemmas  2.2 and 2.3 (dealing with checking Conditions 6 and 7, respectively) and Proposition 1.5 (existence of ancestral paths) are proved in Sect. 6. In Sects. 7–10, respectively, we verify our conditions for the voter model, OP, LT’s and CP, and so establish Theorems 4, 5, 6 and 7.

## Modulus of continuity

In this section we prove Theorems 1 and $1^\prime$.

### Proposition 3.1

Assume Conditions 1, 3, 4, and let $\alpha ,\beta$ satisfy (2.9). There is a constant $C_{3.1}$, and for all $n\ge 1$ a random variable $\delta _n\in (0,1]$ such that if

3.1 $$\begin{aligned} t\in {\mathbb {Z}}_+/n,\ x\in {\mathcal {T}}_t^{\scriptscriptstyle {(n)}},\ 0\le s_1<s_2\le t-n^{-1}, \ s_i\in {\mathbb {Z}}_+/n,\text { and }|s_2-s_1|\le \delta _n, \end{aligned}$$

then

3.2 $$\begin{aligned} (s_1,y_1)\overset{\varvec{a,n}}{\rightarrow }(s_2,y_2)\overset{\varvec{a,n}}{\rightarrow }(t,x) \end{aligned}$$

implies

3.3 $$\begin{aligned} |y_2-y_1|\le C_{3.1}|s_2-s_1|^\alpha . \end{aligned}$$

Moreover $$\delta _n$$ satisfies

3.4 $$\begin{aligned} \mu _n(\delta _n\le \rho )\le C_{3.1}\rho ^\beta , \quad \text { for every }\rho \in [0,1). \end{aligned}$$

### Proof

We first note that it suffices to consider $n=2^{n_0}$ for some $n_0\in {\mathbb {N}}$. Assuming the result for this case, for $n\ge 1$, choose $n_0\in {\mathbb {N}}$ so that $2^{n_0-1}\le n<2^{n_0}$ and set $\delta _n(\omega )=\delta _{2^{n_0}}(\omega )\in (0,1]$. The monotonicity of *m*(*n*) from Condition 1 (survival probability) shows that

$$\begin{aligned} \mu _n(\delta _n\le \rho )\le \mu _{2^{n_0}}(\delta _{2^{n_0}}\le \rho )\le C_{3.1}\rho ^{\beta }, \quad \text { for every }\rho \in [0,1). \end{aligned}$$

Assume now that the conditions in (3.1) are satisfied by $t=k/n$, $x=z/\sqrt{n}$ and $s_i=k_i/n$ where $k,k_i\in {\mathbb {Z}}_+$ and $z\in {\mathbb {Z}}^d$, and that (3.2) is satisfied by $y_i=\frac{x_i}{\sqrt{n}}$, where $x_i\in {\mathbb {Z}}^d$ for $i=1,2$. Then $k_2\le k-1$, which implies $k_2 2^{-n_0}\le k 2^{-n_0}-2^{-n_0}$, and $0<(k_2-k_1)2^{-n_0}<(k_2-k_1)n^{-1}\le \delta _{2^{n_0}}$. By scaling this implies $(\frac{k_1}{2^{n_0}},\frac{x_1}{2^{n_0/2}})\overset{\varvec{a,2^{n_0}}}{\rightarrow }(\frac{k_2}{2^{n_0}},\frac{x_2}{2^{n_0/2}})\overset{\varvec{a,2^{n_0}}}{\rightarrow }(\frac{k}{2^{n_0}},\frac{z}{2^{n_0/2}})$. So the result for $2^{n_0}$ implies that

$$\begin{aligned} |y_2-y_1|&=\frac{2^{n_0/2}}{\sqrt{n}}\Bigl |\frac{x_2}{2^{n_0/2}}-\frac{x_1}{2^{n_0/2}}\Bigr |\le \sqrt{2}C_{3.1}\Bigl (\frac{k_2-k_1}{2^{n_0}}\Bigr )^{\alpha }\le \sqrt{2}C_{3.1}|s_2-s_1|^{\alpha }. \end{aligned}$$

So the result follows for general $$n\ge 1$$ by increasing $$ C_{3.1}$$ to $$\sqrt{2} C_{3.1}$$.

We set $n=2^{n_0}$ for $n_0\in {\mathbb {N}}$. For a natural number *m* define

$$\begin{aligned} I(n_0,m)=\{k\in {\mathbb {Z}}_+:k\le n_0, 2^{n_0-k+1}\le m\}, \end{aligned}$$

and for $k\in I(n_0,m)$ define

$$\begin{aligned} A_k(n_0,m)=\Bigl \{\omega :\max \{|y_2-y_1|:&(m-2^{n_0-k+1},y_1)\overset{\varvec{a}}{\rightarrow }(m-2^{n_0-k},y_2)\\&~~~~~~~~~~~~~~~~~~~~~~~~~~~~~~~~~~~~~\overset{\varvec{a}}{\rightarrow }(m,x)\ \exists \, x\in {\mathcal {T}}_m\}\ge 2^{n_0/2}2^{-k\alpha }\Bigr \}. \end{aligned}$$

Note that the $\max$ exists by (1.3). For $\ell \in {\mathbb {Z}}_+$, let

$$\begin{aligned} B_\ell (n_0,m)={\left\{ \begin{array}{ll}\displaystyle \Bigg [\bigcup _{\begin{array}{c} k> \ell :\\ k\in I(n_0,m) \end{array}}A_k(n_0,m)\Bigg ]\cup A_{n_0}(n_0,m+1),&{}\quad \text {if }\ell \le n_0,\\ \varnothing ,&{}\quad \text {if }\ell >n_0. \end{array}\right. } \end{aligned}$$

### Lemma 3.2

Assume (2.9). There is a constant $C_{3.2}$ so that for all $m\in {\mathbb {N}}$,

- for all $$k\in I(n_0,m)$$, $$\mu _{2^{n_0}}(A_k(n_0,m))\le c_{3.2}2^{-k(p/2-p\alpha -1)}$$,
- $$\begin{aligned} \mu _{2^{n_0}}(B_\ell (n_0,m))\le {\left\{ \begin{array}{ll} c_{3.2}2^{-\ell (p/2-p\alpha -1)},&{}\qquad \text {if } 0\le \ell \le n_0,\\ 0, &{} \qquad \text {if }\ell > n_0.\end{array}\right. } \end{aligned}$$

### Proof

(a) Clearly we have

3.5 $$\begin{aligned}&\mu _{2^{n_0}}(A_k(n_0,m))\nonumber \\&\quad \le m(2^{n_0}){\mathbb {E}}\Bigg [\sum _{y_2\in {\mathcal {T}}_{m-2^{n_0-k}}}\sum _{y_1\in {\mathcal {T}}_{m-2^{n_0-k+1}}}\! \! \! \! \mathbb {1}\big ((m-2^{n_0-k+1},y_1)\overset{\varvec{a}}{\rightarrow }(m-2^{n_0-k},y_2), \nonumber \\&\quad \quad \quad \quad \quad \quad \quad \quad ~~~~~~~~~~~~~~~~~~~~~~~~~~~~~~~~~~~~~~~~|y_2-y_1|\ge 2^{n_0/2}2^{-k\alpha }\big ) \nonumber \\&\quad \quad \quad \quad \quad \quad \quad \quad \quad \quad \quad \quad \quad \quad \quad \quad \times \mathbb {1}\big (\exists x\in {\mathcal {T}}_m\text { s.t. }(m-2^{n_0-k},y_2)\overset{\varvec{a}}{\rightarrow }(m,x)\big )\Bigg ]. \end{aligned}$$

By Condition 3 (conditional survival probability),

3.6 $$\begin{aligned} {\mathbb {P}}\big (\exists x\in {\mathcal {T}}_m\text { s.t. }(m-2^{n_0-k},y_2)\overset{\varvec{a}}{\rightarrow }(m,x)\,\big |\,\mathcal {F}_{m-2^{n_0-k}}\big )\le \frac{c_{3}}{m(2^{n_0-k})}. \end{aligned}$$

Recall that ${\mathcal {T}}_{m-2^{n_0-k}}$ is $\mathcal {F}_{m-2^{n_0-k}}$-measurable, and by AR(iv),

$$\begin{aligned} \mathbb {1}\big ((m-2^{n_0-k+1},y_1)\overset{\varvec{a}}{\rightarrow }(m-2^{n_0-k},y_2)\big )=e_{m-2^{n_0-k+1},m-2^{n_0-k}}(y_1,y_2), \end{aligned}$$

is also $\mathcal {F}_{m-2^{n_0-k}}$-measurable. We therefore can use (3.6) in (3.5) to conclude

$$\begin{aligned}&\mu _{2^{n_0}}(A_k(n_0,m))\\&\quad \le \frac{c_{3} m(2^{n_0})}{m(2^{n_0-k})}{\mathbb {E}}\Bigg [\sum _{y_2\in {\mathcal {T}}_{m-2^{n_0-k}}}\sum _{y_1\in {\mathcal {T}}_{m-2^{n_0-k+1}}}\!\!\!\!\mathbb {1}\big ((m-2^{n_0-k+1},y_1)\overset{\varvec{a}}{\rightarrow }(m-2^{n_0-k},y_2)\big )\\&\quad \quad \quad \quad \quad \quad \quad \quad \quad \quad \quad \quad \quad \quad \quad ~~~~~~~~~~~~~~~~~~~~~\times \frac{|y_2-y_1|^p}{2^{pn_0/2}2^{-pk\alpha }}\Bigg ]\\&\quad \le c_{3}c_{1.21}2^kc_{4}\frac{2^{(n_0-k)p/2}}{2^{pn_0/2}2^{-p\alpha k}}=:c2^{-k(p/2-p\alpha -1)}, \end{aligned}$$

where Condition 4 (spatial increments) and (1.21) are used in the last.

(b) Note first that (2.9) implies $p/2-p\alpha >2$. Sum the bound in (a) over $k>\ell$, note that $n_0\in I(n_0,m+1)$ (to apply (a) to $A_{n_0}(n_0,m+1)$), and use $B_\ell (n_0,m)=\varnothing$ if $\ell > n_0$ to derive (b) (where we can adjust the constants after the fact). $\square$

### Lemma 3.3

Assume (2.9). There is a $c_{3.3}=c_{3.3}(\alpha ,L)$ such that if $m\in {\mathbb {N}}$ and $\ell \in \{0,\dots ,n_0\}$ satisfies $2^{n_0-\ell }\le m$, then

$$\begin{aligned}&B_\ell (n_0,m)^c\subset \Bigl \{\omega :\max \{|x'-y|: (m-2^{n_0-\ell },y)\overset{\varvec{a}}{\rightarrow }(m,x')\overset{\varvec{a}}{\rightarrow }(m+1,x)\\&\quad \text { for some }x\in {\mathcal {T}}_{m+1}\}\le c_{3.3}2^{(n_0/2)-\ell \alpha }\Bigr \}. \end{aligned}$$

### Proof

The conditions on $\ell$ and *m* show that $\{\ell +1,\dots ,n_0\}\subset I(n_0,m)$. This implies that

$$\begin{aligned} B_\ell (n_0,m)^c\subset \cap _{k={\ell +1}}^{n_0}A_k(n_0,m)^c\cap A_{n_0}(n_0,m+1)^c. \end{aligned}$$

Choose $\omega \in B_\ell (n_0,m)^c$ and assume $(m-2^{n_0-\ell },y)\overset{\varvec{a}}{\rightarrow }(m,x')\overset{\varvec{a}}{\rightarrow }(m+1,x)$ for some $x\in {\mathcal {T}}_{m+1}$. By (1.10) we may choose $x''$ so that $(m-2^{n_0-\ell },y)\overset{\varvec{a}}{\rightarrow }(m-1,x'')\overset{\varvec{a}}{\rightarrow }(m,x')$ (set $x''=y$ if $\ell =n_0$). Then since $\omega \not \in A_{n_0}(n_0,m+1)$,

3.7 $$\begin{aligned} |x'-x''|<2^{n_0/2-n_0\alpha }. \end{aligned}$$

Let $y_\ell =y$. By (1.10) for $k=\ell +1,\dots ,n_0$ we may choose $y_k\in {\mathcal {T}}_{m-2^{n_0-k}}$ such that $y_{n_0}=x''$, and

$$\begin{aligned} (m-2^{n_0-k+1},y_{k-1})\overset{\varvec{a}}{\rightarrow }(m-2^{n_0-k},y_k)\text { for }k=\ell +1,\dots ,n_0. \end{aligned}$$

By $\omega \notin A_k(n_0,m)$ and the triangle inequality, we have

$$\begin{aligned} |x''-y|=|y_{n_0}-y_\ell |&\le \sum _{k=\ell +1}^{n_0}|y_k-y_{k-1}|\le 2^{n_0/2}\sum _{k=\ell +1}^{n_0}2^{-k\alpha }\le C2^{n_0/2}2^{-\ell \alpha }. \end{aligned}$$

This and (3.7) imply $|x'-y|\le C2^{(n_0/2)-\ell \alpha }$ and so completes the proof. $\square$

Returning now to the proof of Proposition 3.1, we define

$$\begin{aligned}&C_r(n_0)=\cup _{\ell =r}^{n_0}\cup _{i=1}^{\lceil 2^{\ell (1+\beta )}\rceil }B_\ell (n_0,i2^{n_0-\ell })\text { for }r\in {\mathbb {Z}}_+\ (C_r(n_0)=\varnothing \text { if }r>n_0),\\&K^1_{n_0}=\min \{r\in \{0,\dots ,n_0+1\}:\omega \notin C_r(n_0)\}\le n_0+1,\\&K^2_{n_0}=\min \{r\in {\mathbb {Z}}_+:2^{r\beta +n_0}\ge S^{\scriptscriptstyle (1)}\},\\&K_{n_0}=(K^1_{n_0}\vee K^2_{n_0})\wedge (n_0+1)\in {\mathbb {Z}}_+. \end{aligned}$$

Note that $C_r(n_0)$ is non-increasing in *r*. Set $\delta _{2^{n_0}}(\omega )=2^{-K_{n_0}(\omega )}\in (0,1]$. Then for $r\in \{0,\dots ,n_0\}$,

$$\begin{aligned} \mu _{2^{n_0}}(K_{n_0}>r)&\le \mu _{2^{n_0}}(C_r(n_0)) +\mu _{2^{n_0}}(S^{\scriptscriptstyle (1)}>2^{r\beta +n_0})\\&\le \left[ \sum _{\ell =r}^{n_0}\sum _{i=1}^{\lceil 2^{\ell (1+\beta )}\rceil } c_{3.2}2^{-\ell (p/2-p\alpha -1)}\right] +\frac{m(2^{n_0})c_{3}}{m(2^{r\beta }2^{n_0})}, \end{aligned}$$

where we have used Lemma 3.2(b) and Condition 3 with $(s,y))=(0,o)$ and $t=2^{r\beta }2^{n_0}$. Since $2^{r\beta }\le 2^{n_0}$ (recall $\beta \le 1$) we may use (1.21) to see that

$$\begin{aligned} \frac{m(2^{n_0})}{m(2^{r\beta }2^{n_0})}\le c_{1.21}2^{-r\beta }, \end{aligned}$$

and so from the above,

3.8 $$\begin{aligned} \mu _{2^{n_0}}(K_{n_0}>r)\le c_{3.2}\sum _{\ell =r}^{n_0}2^{1-\ell (p/2-2-p\alpha -\beta )}+c_{3}c_{1.21}2^{-r\beta }\le C2^{-r\beta }, \end{aligned}$$

where (2.9) is used in the last inequality. If $r\in \{n_0+1,n_0+2,\dots \}$ then (3.8) is trivial since the left-hand side is zero. If $\rho \in (0,1]$ choose $r\in {\mathbb {Z}}_+$ so that $2^{-r-1}<\rho \le 2^{-r}$. Then by (3.8),

$$\begin{aligned} \mu _{2^{n_0}}(\delta _{2^{n_0}}<\rho )\le \mu _{2^{n_0}}(K_{n_0}>r)\le C2^{-r\beta }\le C2^\beta \rho ^\beta . \end{aligned}$$

Take limits from the right in $\rho <1$ in the above to derive (3.4).

Turning now to (3.3), we can rescale and restate our objective as (note that $t=(m+1)/n$ for some $m\in {\mathbb {N}}$ or the conclusion is vacuously true):

If

$$\begin{aligned}&k_1,k_2\in {\mathbb {Z}}_+, m\in {\mathbb {N}}, x\in {\mathcal {T}}_{m+1}, 0\le k_1<k_2\le m\text { satisfies }\\&|k_2-k_1|\le 2^{n_0-K_{n_0}},\text { and }(k_1,y_1)\overset{\varvec{a}}{\rightarrow }(k_2,y_2)\overset{\varvec{a}}{\rightarrow }(m+1,x), \end{aligned}$$

then

$$\begin{aligned} |y_2-y_1|\le C_{3.1}|k_2-k_1|^{\alpha }2^{(n_0/2)-(n_0\alpha )}. \end{aligned}$$

It suffices to consider $k_2=m$ in the above because if we choose (by (1.10)) $x'$ s.t. $(k_2,y_2)\overset{\varvec{a}}{\rightarrow }(k_2+1,x')\overset{\varvec{a}}{\rightarrow }(m+1,x)$, then we can work with $(k_2+1,x')$ in place of $(m+1,x)$. So let us assume

3.9 $$\begin{aligned}&k\in {\mathbb {Z}}_+, \,m\in {\mathbb {N}}, \,x\in {\mathcal {T}}_{m+1}, \,0\le k< m, \, |m-k|\le 2^{n_0-K_{n_0}}, \nonumber \\&\qquad \text {and }(k,y)\overset{\varvec{a}}{\rightarrow }(m,x')\overset{\varvec{a}}{\rightarrow }(m+1,x). \end{aligned}$$

We must show that

3.10 $$\begin{aligned} |x'-y|\le C_{3.1}|m-k|^{\alpha }2^{(n_0/2)-(n_0\alpha )}. \end{aligned}$$

If $K_{n_0}=n_0+1$, then (3.9) leads to a contradiction and so we have

3.11 $$\begin{aligned} K_{n_0}\le n_0,\text { and so }K_{n_0}=K_{n_0}^1\vee K_{n_0}^2\le n_0. \end{aligned}$$

By (3.9), $2^{-n_0}\le (m-k)2^{-n_0}\le 2^{-K_{n_0}}$, and so we may choose $r\in {\mathbb {Z}}_+$ so that

3.12 $$\begin{aligned} 2^{-r-1}<\frac{m-k}{2^{n_0}}\le 2^{-r},\quad K_{n_0}\le r\le n_0. \end{aligned}$$

Using the fact that $0<(m-k)2^{-n_0}\le 2^{-r}$, we can choose $i_r,j_r\in {\mathbb {Z}}_+$ with $j_r-i_r\in \{0,1\}$ and $i_\ell ,j_\ell \in \{0,1\}$ for $\ell \in (r,n_0]\cap {\mathbb {N}}$ so that

$$\begin{aligned} m2^{-n_0}=j_r2^{-r}+\sum _{\ell =r+1}^{n_0}j_\ell 2^{-\ell },\quad k2^{-n_0}=i_r 2^{-r}+\sum _{\ell =r+1}^{n_0}i_\ell 2^{-\ell }. \end{aligned}$$

For $$q\in (r,n_0]\cap {\mathbb {N}}$$ define

$$\begin{aligned}&m_r=j_r2^{n_0-r},\quad m_q=m_r+\sum _{\ell =r+1}^qj_\ell 2^{n_0-\ell },\\&k_r=i_r2^{n_0-r}, \quad k_q=k_r+\sum _{\ell =r+1}^qi_\ell 2^{n_0-\ell }, \end{aligned}$$

so that $k_{n_0}=k$ and $m_{n_0}=m$. By Lemma 1.2 there are $(y_q)_{q=r}^{n_0}$ and $(z_q)_{q=r}^{n_0}$ s.t. $y_{n_0}=y$, $z_{n_0}=x'$ and

3.13 $$\begin{aligned} (k_{q-1},y_{q-1})\overset{\varvec{a}}{\rightarrow }(k_q,y_q),\quad (m_{q-1},z_{q-1})\overset{\varvec{a}}{\rightarrow }(m_q,z_q)\quad \text {for }q \in (r,n_0]\cap {\mathbb {N}}\end{aligned}$$

(if $k_{q-1}=k_q$ set $y_q=y_{q-1}$ and similarly if $m_{q-1}=m_q$). In addition if $i_r=j_r$ so that $k_r=m_r$ we may assume

3.14 $$\begin{aligned} y_r=z_r\quad \text { if }\quad i_r=j_r. \end{aligned}$$

On the other hand if $j_r=i_r+1$, then

$$\begin{aligned} k_{n_0}\le k_r+\sum _{\ell =r+1}^{n_0}2^{n_0-\ell }<k_r+2^{n_0-r}=(i_r+1)2^{n_0-r}=m_r, \end{aligned}$$

and so (by Lemma 1.2) we may choose $z_r$ in the above s.t. $(k_{n_0},y_{n_0})\overset{\varvec{a}}{\rightarrow }(m_r,z_r)$. Hence we have

3.15 $$\begin{aligned} \text {if }j_r&=i_r+1,\text { then }(k_r,y_r)\overset{\varvec{a}}{\rightarrow }\cdots \overset{\varvec{a}}{\rightarrow }(k_{n_0},y_{n_0})\overset{\varvec{a}}{\rightarrow }(m_r,z_r)\nonumber \\&\overset{\varvec{a}}{\rightarrow }(m,x')\overset{\varvec{a}}{\rightarrow }(m+1,x). \end{aligned}$$

Recalling from (3.11), and (3.12) that $r\ge K_{n_0}\ge K^1_{n_0}$, we have from the definition of $K^1_{n_0}$ that $\omega \notin C_r(n_0)$ and so for all $\ell \in [r,n_0]\cap {\mathbb {Z}}_+$,

$$\begin{aligned} \omega \in \cap _{i=1}^{\lceil 2^{\ell (1+\beta )}\rceil }B_\ell (n_0,i2^{n_0-\ell })^c, \end{aligned}$$

which by Lemma 3.3 (and $2^{n_0-\ell }\le i2^{n_0-\ell }$) implies

3.16 $$\begin{aligned}&\forall \, 1\le i\le \lceil 2^{\ell (1+\beta )} \rceil \text { if }((i-1)2^{n_0-\ell },y)\overset{\varvec{a}}{\rightarrow }(i2^{n_0-\ell },y')\overset{\varvec{a}}{\rightarrow }(i2^{n_0-\ell }+1,z)\nonumber \\&\quad \text {for some }z\in {\mathcal {T}}_{i2^{n_0-\ell }+1},\text { then }|y-y'|\le c_{3.3}2^{n_0/2}2^{-\ell \alpha }. \end{aligned}$$

As $r\ge K_{n_0}\ge K^2_{n_0}$, we also have $S^{\scriptscriptstyle (1)}\le 2^{r\beta +n_0}\le 2^{\ell \beta +n_0}$ for all $\ell \ge r$ and so if $i>\lceil 2^{\ell (1+\beta )}\rceil$, then

$$\begin{aligned} i2^{n_0-\ell }>2^{\ell (1+\beta )}2^{n_0-\ell }=2^{\ell \beta +n_0}\ge S^{\scriptscriptstyle (1)}, \end{aligned}$$

and so ${\mathcal {T}}_{i2^{n_0-\ell }+1}=\varnothing$. This implies (3.16) holds vacuously and we may conclude

3.17 $$\begin{aligned}&\forall \, i \in {\mathbb {N}}\text { if }((i-1)2^{n_0-\ell },y)\overset{\varvec{a}}{\rightarrow }(i2^{n_0-\ell },y')\overset{\varvec{a}}{\rightarrow }(i2^{n_0-\ell }+1,z) \nonumber \\&\quad \text {for some }z\in {\mathcal {T}}_{i2^{n_0-\ell }+1},\text { then }|y-y'|\le c_{3.3}2^{n_0/2}2^{-\ell \alpha }. \end{aligned}$$

By $y_{n_0}=y$, $z_{n_0}=x'$ and the triangle inequality, we have

3.18 $$\begin{aligned} |x'-y|=|z_{n_0}-y_{n_0}|\le \sum _{q=r+1}^{n_0}\Bigl [|z_q-z_{q-1}|+|y_{q}-y_{q-1}|\Bigr ]+|z_r-y_r|. \end{aligned}$$

Note that for $q\in (r,n_0]\cap {\mathbb {N}}$, $m_q={{\bar{j}}}_q2^{n_0-q}$ for some ${{\bar{j}}}_q\in {\mathbb {Z}}_+$ and also $m_{q-1}=({{\bar{j}}}_q-j_q)2^{n_0-q}$, where $j_q=0$ or 1. Therefore (3.13) implies

$$\begin{aligned} (({{\bar{j}}}_q-j_q)2^{n_0-q},z_{q-1})\overset{\varvec{a}}{\rightarrow }({{\bar{j}}}_q 2^{n_0-q},z_q)\overset{\varvec{a}}{\rightarrow }(m+1,x), \end{aligned}$$

and so by (1.10) there is a $z\in {\mathcal {T}}_{{{\bar{j}}}_q 2^{n_0-q}+1}$ s.t.

$$\begin{aligned} (({{\bar{j}}}_q-j_q)2^{n_0-q},z_{q-1})\overset{\varvec{a}}{\rightarrow }({{\bar{j}}}_q 2^{n_0-q},z_q)\overset{\varvec{a}}{\rightarrow }({\bar{j}}_q 2^{n_0-q}+1,z). \end{aligned}$$

Therefore (3.17) implies

3.19 $$\begin{aligned} |z_q-z_{q-1}|\le c_{3.3}2^{n_0/2}2^{-q \alpha }\quad \text {for }q\in (r,n_0]\cap {\mathbb {N}}\end{aligned}$$

(note here that if ${{\bar{j}}}_q=0$, then $j_q=0$ and so $z_q=z_{q-1}$ and the above inequality is trivial). Similar reasoning shows

$$\begin{aligned} |y_q-y_{q-1}|\le c_{3.3}2^{n_0/2}2^{-q\alpha }\quad \text {for }(r,n_0]\cap {\mathbb {N}}. \end{aligned}$$

To handle the last term in (3.18), first observe that if $j_r-i_r=1$, then $k_r=(j_r-1)2^{n_0-r}$ and so (by (3.15)) $(k_r,y_r)\overset{\varvec{a}}{\rightarrow }(m_r,z_r)\overset{\varvec{a}}{\rightarrow }(m+1,x)$ implies

$$\begin{aligned} (( j_r-1)2^{n_0-r},y_r)\overset{\varvec{a}}{\rightarrow }(j_r 2^{n_0-r},z_r)\overset{\varvec{a}}{\rightarrow }((j_r 2^{n_0-r}+1,z) \end{aligned}$$

for some $z\in {\mathcal {T}}_{j_r2^{n_0-r}+1}$ (by (1.10)). It follows from (3.17) with $\ell =r$ that

3.20 $$\begin{aligned} \text {if }j_r-i_r=1,\text { then }|z_r-y_r|\le c_{3.3}2^{n_0/2}2^{-r \alpha }. \end{aligned}$$

Use (3.19)–(3.20) and (3.14) in (3.18) and so conclude that

$$\begin{aligned} |x'-y|&\le \Bigg [2c_{3.3}2^{n_0/2}\sum _{q=r+1}^{n_0}2^{-q\alpha }\Bigg ]+c_{3.3}2^{n_0/2}2^{-r\alpha }\\&\le C2^{n_0/2}2^{-r\alpha }\le C2^\alpha \Bigl (\frac{m-k}{2^{n_0}}\Bigr )^\alpha 2^{n_0/2}\quad (\text {by }(3.12)), \end{aligned}$$

which gives (3.10), as required. $\square$

*Proof of Theorem* $1^\prime$. Let $n\in [1,\infty )$ and take $\delta _n$ as in Proposition 3.1, so that (2.16) and $\delta _n\in (0,1]$ are immediate from that proposition. Assume $s_1,s_2,y_1,y_2$ are as in (2.14). If $s_1=s_2$ then $y_1=y_2$ by (AR)(i), and the result is trivial. Otherwise $s_1<s_2$, and by (1.10) we may choose $x'$ s.t.

$$\begin{aligned} (s_1,y_1)\overset{\varvec{a,n}}{\rightarrow }(s_2-n^{-1},x')\overset{\varvec{a,n}}{\rightarrow }(s_2,y_2). \end{aligned}$$

We also have $|s_2-n^{-1}-s_1|\le |s_2-s_1|\le \delta _n$. Proposition 3.1 and (2.3) imply

$$\begin{aligned} |y_2-y_1|&\le |y_2-x'|+|x'-y_1|\\&\le \frac{\sqrt{d} L}{\sqrt{n}}+C_{3.1}|s_2-n^{-1}-s_1|^\alpha \\&\le (\sqrt{d} L+C_{3.1})|s_2-s_1|^\alpha , \end{aligned}$$

the last since $|s_2-s_1|\ge 1/n$ and $\alpha <1/2$. This proves (2.15) where $C_{1'}=\sqrt{d} L+C_{3.1}$.

Now suppose instead that $s_1,s_2\in {\mathbb {R}}_+$ (otherwise as above). The validity of (2.12) now follows by an elementary argument using (2.3) and the fact that for $0\le s_1<s_2$, $(s_1,y_1)\overset{\varvec{a,n}}{\rightarrow }(s_2,y_2)$ iff $(\lfloor s_1n \rfloor /n,y_1)\overset{\varvec{a,n}}{\rightarrow }(\lfloor s_2n \rfloor /n,y_2)$. $\square$

Consider next the proof of Theorem 1 and assume the hypotheses of that theorem. We first use Conditions 5 (local jumps) and 2 (total mass) to handle the small increments of $w^{\scriptscriptstyle {(n)}}(t,x)$ near *t*.

### Lemma 3.4

There is a $C_{3.4}$ such that for any $\alpha \in (0,1/2)$, $t^*\ge 1$ and $n\ge 1$,

$$\begin{aligned}&\mu _n\Bigl (\max \{|y-x|:(s,y)\overset{\varvec{a,n}}{\rightarrow }(t,x), t\in [s,s+(2/n)], s\in {\mathbb {Z}}_+/n,s\le t^*\} >n^{-\alpha }\Bigr )\\&\quad \le C_{3.4}t^*n^{2-\kappa ((1/2)-\alpha )}. \end{aligned}$$

### Proof

If $s=j/n\in {\mathbb {Z}}_+/n$ is fixed, then by scaling,

$$\begin{aligned}&\mu _n\Bigl (\max \{|y-x|:(s,y)\overset{\varvec{a,n}}{\rightarrow }(t,x), t\in [s,s+(2/n)]\}>n^{-\alpha }\Bigr )\\&\quad =\mu _n\Bigl (\max \{|y-x|:(j,y)\overset{\varvec{a}}{\rightarrow }(t,x), t\in [j,j+2]\}>n^{(1/2)-\alpha }\Bigr )\\&\quad \le m(n)\sum _{y\in {\mathbb {Z}}^d}{\mathbb {E}}\Big [\mathbb {1}(y\in {\mathcal {T}}_j){\mathbb {P}}(\exists \ t\in [j,j+2]\text { and }x\in {\mathcal {T}}_t\text { s.t. }\\&\quad \quad \quad \quad \quad \quad \quad \quad \quad \quad |x-y|>n^{(1/2)-\alpha }\text { and }(j,y)\overset{\varvec{a}}{\rightarrow }(t,x)|\mathcal {F}_j)\Big ]. \end{aligned}$$

Now use Conditions 5 and 2, and (1.23) to see the above is at most

$$\begin{aligned} c_{1.23}nc_{3}c_{5}n^{-\kappa ((1/2)-\alpha )}. \end{aligned}$$

Finally sum over $s\in ({\mathbb {Z}}_+/n)\cap [0,t^*]$ to obtain the desired upper bound. $\square$

### Proof of Theorem 1

Fix $n\ge 1$ and $q\in (0,1]$ as in the Theorem and define

$$\begin{aligned} \varOmega _n=&\{S^{\scriptscriptstyle {(n)}}\le n^q\}\\&\cap \Bigl \{\max \{|y-x|:(s,y)\overset{\varvec{a,n}}{\rightarrow }(t,x), t\in [s,s+2],s\in {\mathbb {Z}}_+/n,s\le n^q\}\le n^{-\alpha }\Bigr \}. \end{aligned}$$

By Lemma 3.4 and (1.31) and the fact that $n^q\le n$,

3.21 $$\begin{aligned} \mu _n(\varOmega _n^c)\le \frac{C}{n^q}+C_{3.4}n^{q+2 -\kappa ((1/2)-\alpha )}&\le \frac{C}{n^q}, \end{aligned}$$

where the definition of *q* is used in the last line. To avoid confusion we denote the $\delta _n$ arising in Proposition 3.1 by $\delta _n^{3.1}$ and then define

$$\begin{aligned} \delta _n(\omega )={\left\{ \begin{array}{ll} \delta _n^{3.1}(\omega ),&{}\text { if }\omega \in \varOmega _n,\\ 0,&{}\text { if }\omega \in \varOmega _n^c. \end{array}\right. } \end{aligned}$$

Then for $$\rho \in [0,1)$$,

$$\begin{aligned} \mu _n(\delta _n\le \rho )\le \mu _n(\varOmega _n^c)+\mu _n(\delta _n^{3.1}\le \rho )\le \frac{C}{n^q}+C_{3.1}\rho ^\beta , \end{aligned}$$

the last by (3.21) and Proposition 3.1. This proves (2.13).

In proving (2.11) implies (2.12) to reduce subscripts we assume

$$\begin{aligned} 0\le s<t, \quad |t-s|\le \delta _n, \quad \text { and }(s,y)\overset{\varvec{a,n}}{\rightarrow }(t,x). \end{aligned}$$

This implies $\delta _n>0$ and so $\omega \in \varOmega _n$, which in turn implies $S^{\scriptscriptstyle {(n)}}\le n^q$ and so (as ${\mathcal {T}}_t^{\scriptscriptstyle {(n)}}$ is non-empty),

3.22 $$\begin{aligned} t\le S^{\scriptscriptstyle {(n)}}\le n^q. \end{aligned}$$

We must show that, for an appropriate $C_{1}$,

3.23 $$\begin{aligned} |x-y|\le C_{1}[|t-s|^\alpha +n^{-\alpha }]. \end{aligned}$$

If $t\le 2/n$ this follows easily from $\omega \in \varOmega _n$ and the triangle inequality, so assume without loss of generality that $t>2/n$. Write $[u]_n=\lfloor nu \rfloor /n$, and set $t_n=[t-n^{-1}]_n, t'_n=t_n+n^{-1}, s_n=[s]_n\wedge t_n\le t'_n$, all in $\in {\mathbb {Z}}_+/n$. Therefore

3.24 $$\begin{aligned} 0\le t_n-s_n\le t-n^{-1}-(s-n^{-1})=t-s\le \delta _n \end{aligned}$$

(we can assume $s_n=[s]_n$ in the above derivation, since otherwise $s_n=t_n$ and the desired upper bound in (3.24) is trivial), and

3.25 $$\begin{aligned} 0\le t-t_n\le 2/n,\ 0\le s-s_n\le 2/n. \end{aligned}$$

Since $s_n\le s$ and $y\in {\mathcal {T}}^{\scriptscriptstyle {(n)}}_s$ by Lemma 1.2 there exists $y_n$ s.t.

$$\begin{aligned} (s_n,y_n)\overset{\varvec{a,n}}{\rightarrow }(s,y). \end{aligned}$$

We also have $s_n\le t_n<t_n+n^{-1}\le t\le n^q$ (by (3.22)) and $x\in {\mathcal {T}}^{\scriptscriptstyle {(n)}}_t$, so by Lemma 1.2 there are $x_n,x_n'$ s.t.

3.26 $$\begin{aligned} (s_n,y_n)\overset{\varvec{a,n}}{\rightarrow }(t_n,x_n)\overset{\varvec{a,n}}{\rightarrow }(t_n+n^{-1},x_n')\overset{\varvec{a,n}}{\rightarrow }(t,x). \end{aligned}$$

Therefore by $\omega \in \varOmega _n$ and (3.25) we may conclude,

$$\begin{aligned} |x-y|&\le |x-x_n|+|x_n-y_n|+|y_n-y|\\&\le n^{-\alpha }+|x_n-y_n|+n^{-\alpha }\\&\le 2n^{-\alpha }+C_{3.1}|t_n-s_n|^\alpha \quad (\text { by } (3.24), (3.26) \text { and Proposition }3.1)\\&\le 2n^{-\alpha }+C_{3.1}|t-s|^\alpha , \end{aligned}$$

the last by (3.24). This proves (3.23) and the proof is complete. $\square$

## Convergence of the range

In this section we prove Theorem 2. Recall that ${\mathbb {N}}_o^s(\cdot )={\mathbb {N}}_o(\cdot \,|S>s)$.

### Lemma 4.1

4.1 $$\begin{aligned} {\mathbb {N}}_o^1\left( \int _1^2X_s(1)ds\le a \right)&\sim \frac{4}{ \sqrt{2\pi \gamma }} \sqrt{a}, \qquad \text { as }a \downarrow 0 \nonumber \\ {\mathbb {N}}^1_o\left( \int _1^2X_s(1)ds\le a\right)&\le 2 \sqrt{\frac{2}{\gamma }}{\mathrm {e}}\sqrt{a}, \qquad \text { for every }a>0. \end{aligned}$$

### Proof

First recall from (1.29) that ${\mathbb {N}}_o(S>1)=2/\gamma$, and by Theorem II.7.2(iii) of [32]

4.2 $$\begin{aligned} {\mathbb {N}}_o^1\big (X_1(1) \in {\mathrm {d}}x \big )=(2/\gamma ){\mathrm {e}}^{-(2/\gamma )x}{\mathrm {d}}x. \end{aligned}$$

Let $v^{(\lambda )}_t=\sqrt{\frac{2\lambda }{\gamma }}\frac{[{\mathrm {e}}^{t \sqrt{2\gamma \lambda }}-1]}{[{\mathrm {e}}^{t \sqrt{2\gamma \lambda }}+1]}$ be the unique solution of

$$\begin{aligned} \frac{{\mathrm {d}}v}{{\mathrm {d}}t}=\frac{-\gamma v_t^2}{2}+\lambda , \qquad v(0)=0. \end{aligned}$$

Then the Markov property under ${\mathbb {N}}_o$ and exponential duality (see Theorem II.5.11(c) of [32]), together with (4.2) gives

$$\begin{aligned} {\mathbb {N}}_o^1&\left[ \exp \Bigg \{-\lambda \int _1^2X_s(1)ds\Bigg \}\right] \\&={\mathbb {N}}_o\left[ {\mathbb {E}}_{X_1}\Bigg [\exp \Bigg \{-\lambda \int _0^1X_s(1)ds\Bigg \}\mathbb {1}_{\{S>1\}}\Bigg ]\right] /{\mathbb {N}}_o(S>1)\\&=\int _0^\infty \exp \{-x v_1^{(\lambda )}\}\frac{2}{\gamma } {\mathrm {e}}^{-2x/\gamma } {\mathrm {d}}x \quad =\quad \frac{2}{2+\gamma v_1^{(\lambda )}}. \end{aligned}$$

Since $$v_1^{(\lambda )}\sqrt{\gamma /(2 \lambda )} \rightarrow 1$$ as $$\lambda \rightarrow \infty $$,

$$\begin{aligned} {\mathbb {N}}_o^1\left[ \exp \Bigg \{-\lambda \int _1^2X_s(1)ds\Bigg \} \right] \sim \sqrt{\frac{2}{\gamma \lambda }}, \qquad \text { as } \lambda \rightarrow \infty . \end{aligned}$$

A Tauberian theorem (e.g. Theorems 2,3 in Section XIII.5 of [15]) now gives

$$\begin{aligned} {\mathbb {N}}_o^1\left( \int _1^2X_s(1)ds\le a\right) \sim \frac{4}{ \sqrt{2\pi \gamma }} \sqrt{a}, \qquad \text { as }a \downarrow 0. \end{aligned}$$

Now

$$\begin{aligned} v_1^{(\lambda )}&=\sqrt{\frac{2\lambda }{\gamma }}\frac{[{\mathrm {e}}^{ \sqrt{2\gamma \lambda }}-1]}{[{\mathrm {e}}^{ \sqrt{2\gamma \lambda }}+1]}=\sqrt{\frac{2\lambda }{\gamma }}\left[ 1-\frac{2}{{\mathrm {e}}^{ \sqrt{2\gamma \lambda }}+1}\right] \ge \frac{1}{2}\sqrt{\frac{2\lambda }{\gamma }}, \quad \text { if }\lambda \ge \gamma ^{-1}. \end{aligned}$$

If $a\le \gamma$ and so $\lambda :=1/a\ge \gamma ^{-1}$, then using $\mathbb {1}_{Y\le a}\le {\mathrm {e}}^{\lambda (a-Y)}$ we have

$$\begin{aligned} {\mathbb {N}}_o^1\left( \int _1^2X_s(1)ds\le a \right)&\le {\mathrm {e}}^{\lambda a} {\mathbb {N}}^1_o\left[ \exp \Bigg \{-\lambda \int _1^2X_s(1) {\mathrm {d}}s\Bigg \} \right] \\&\le {\mathrm {e}}^{\lambda a} \frac{2}{2+\gamma v_1^{(\lambda )}}\le \dfrac{ 2{\mathrm {e}}}{2+\sqrt{\frac{\lambda \gamma }{2}}} \le \frac{2\sqrt{2} {\mathrm {e}}}{\sqrt{\gamma }} \sqrt{a}. \end{aligned}$$

For $a>\gamma$ the bound is trivial. $\square$

### Lemma 4.2

$$0\in R\ \ {\mathbb {N}}_o-a.e.$$

### Proof

This is immediate from the description of the integral of SBM in terms of the Brownian snake, and the continuity of the snake under ${\mathbb {N}}_o$. See Proposition 5 and Section 5 of Chapter IV of [29]. $\square$

Recall that $\mathcal {K}$ is the Polish space of compact subsets of ${\mathbb {R}}^d$, equipped with the Hausdorff metric $d_0$ and $\varDelta _1$ is as in (1.2).

### Lemma 4.3

If $\nu _n \rightarrow \nu$ in $M_F({\mathbb {R}}^d)$, and ${\mathrm {supp}}(\nu )\in \mathcal {K}$, then

$$\begin{aligned} \varDelta _1({\mathrm {supp}}(\nu ),{\mathrm {supp}}(\nu _n)) \rightarrow 0. \end{aligned}$$

### Proof

Fix $\varepsilon >0$. We must show that ${\mathrm {supp}}(\nu )\subset {\mathrm {supp}}(\nu _n)^\varepsilon$ for all *n* sufficiently large. Let $x\in {\mathrm {supp}}(\nu )$. Then $\liminf _{n \rightarrow \infty }\nu _n(B(x,\varepsilon /2))\ge \nu (B(x,\varepsilon /2))>0$. Therefore there exists *n*(*x*) such that for every $n\ge n(x)$,

$$\begin{aligned} \nu _n(B(x,\varepsilon /2))\ge \frac{1}{2} \nu (B(x,\varepsilon /2))>0, \end{aligned}$$

and therefore $x \in {\mathrm {supp}}(\nu _n)^{\varepsilon /2}$. As ${\mathrm {supp}}(\nu )$ is compact there exist $x_1,\dots , x_k \in {\mathrm {supp}}(\nu )$ such that ${\mathrm {supp}}(\nu )\subset \cup _{i=1}^k B(x_i,\varepsilon /2)$. Thus if $n\ge n_0=\max _{i\le k}n(x_i)$ then

$$\begin{aligned} {\mathrm {supp}}(\nu )\subset \cup _{i=1}^kB(x_i,\varepsilon /2)\subset {\mathrm {supp}}(\nu _n)^\varepsilon , \end{aligned}$$

as required. $$\square $$

In the rest of the Section we will assume Conditions 1–7, let $\alpha ,\beta$ satisfy (2.9) and (2.10), and assume $(\gamma , \sigma _0^2)$ are as in Condition 6 (measure convergence). The parameter *q* is as in Theorem 1. We start with some simple consequences of Theorem 1. Recall from (1.37) that $R^{\scriptscriptstyle {(n)}}$ is a.s. finite. For $t\ge 0$, define

$$\begin{aligned} R^{\scriptscriptstyle {(n)}}_t=\cup _{s\le t}\mathcal {T}^{\scriptscriptstyle {(n)}}_s\subset R^{\scriptscriptstyle {(n)}}. \end{aligned}$$

### Lemma 4.4

- There is a $c_{4.4}>0$ such that on $\{\delta _n\ge n^{-1}\}$ we have
  4.3 $$\begin{aligned} r_0(R^{\scriptscriptstyle {(n)}})\le c_{4.4}(S^{\scriptscriptstyle {(n)}}\delta _n^{\alpha -1}+1). \end{aligned}$$
- $$\eta _{4.4}(u):=\sup _{n\ge 1}\mu _n(r_0(R^{\scriptscriptstyle {(n)}})\ge u)\rightarrow 0\text { as }u\rightarrow \infty $$.
- For any $\tau _0,\varepsilon ,s>0$ there is a $\tau =\tau (\tau _0,\varepsilon ,s)>0$ and $n_0(\tau _0,\varepsilon ,s)\ge 1$ so that
  $$\begin{aligned} {\mathbb {P}}(R_\tau ^{\scriptscriptstyle {(n)}}\not \subset B(0,\tau _0)|S^{\scriptscriptstyle {(n)}}>s)\le \varepsilon \quad \forall n\ge n_0. \end{aligned}$$

### Proof

(a) Assume $\delta _n(\omega )\ge 1/n$. Assume also $x\in {\mathcal {T}}_s^{\scriptscriptstyle {(n)}}$ for some $s> 0$. Choose $M\in {\mathbb {N}}$ so that $(M-1)\delta _n<s\le M\delta _n$, and set $s_i=i\delta _n$ for $0\le i<M$ and $s_M=s$. Clearly $s<S^{\scriptscriptstyle {(n)}}$ (since $\mathcal {T}^{\scriptscriptstyle {(n)}}_s$ is non-empty) and so

4.4 $$\begin{aligned} M\le s\delta _n^{-1}+1\le S^{\scriptscriptstyle {(n)}}\delta _n^{-1}+1. \end{aligned}$$

By Lemma 1.2 there are $y_i\in {\mathcal {T}}_{s_i}$ for $0\le i\le M$ s.t. $y_0=o$, $y_M=x$ and $(s_{i-1},y_{i-1})\overset{\varvec{a,n}}{\rightarrow }(s_i,y_i)$ for $1\le i\le M$. Theorem 1 implies for all $1\le i\le M$, $|y_i-y_{i-1}|\le 2C_{1}\delta _n^{\alpha }$, and so by the triangle inequality, (4.4), and $\delta _n\le 1$,

$$\begin{aligned} |x|=|y_M|\le M 2C_{1}\delta _n^{\alpha }\le 2C_{1}[S^{\scriptscriptstyle {(n)}}\delta _n^{\alpha -1}+1]. \end{aligned}$$

This gives (a) with $c_{4.4}=2C_{1}$.

(b) Use (a) to see that for $u,n\ge 1$ and $u> 2c_{4.4}$,

4.5 $$\begin{aligned}&\mu _n (r_0(R^{\scriptscriptstyle {(n)}})\ge u) \nonumber \\&\quad \le \mu _n(\delta _n\le u^{-1}\vee n^{-1})+\mu _n(S^{\scriptscriptstyle {(n)}}\delta _n^{\alpha -1}\ge (u/c_{4.4})-1,\delta _n\ge 1/u) \nonumber \\&\quad \le C_{1}(u^{-\beta }+n^{-\beta }+n^{-q})+\mu _n(S^{\scriptscriptstyle {(n)}}\ge u^{\alpha -1}[(u/c_{4.4})-1]> u^\alpha /(2c_{4.4})), \end{aligned}$$

where in the last line we have used Theorem 1 and the lower bound on *u*. Now (1.31) implies that

$$\begin{aligned} \mu _n(S^{\scriptscriptstyle {(n)}}>u^\alpha /(2c_{4.4}))&\le \overline{s}_Dc_{1.21}/((u^\alpha /2c_{4.4})\wedge n). \end{aligned}$$

Using this in the bound (4.5) we see that for any $\varepsilon >0$ there is an $n_0\ge 1$ such that

4.6 $$\begin{aligned} \mu _n(r_0(R^{\scriptscriptstyle {(n)}})\ge u)<\varepsilon \qquad \text {for all } n,u\ge n_0. \end{aligned}$$

But for $n \in [1,n_0)\cap {\mathbb {N}}$ we have

$$\begin{aligned} \mu _n(r_0(R^{\scriptscriptstyle {(n)}})\ge u)\le m(n_0){\mathbb {P}}(r_0(R^{\scriptscriptstyle (1)})\ge u)<\varepsilon , \end{aligned}$$

for $u>u_0(\varepsilon )$. The result follows from this last inequality and (4.6).

(c) Fix $\tau _0,\varepsilon ,s>0$ and then choose $n_1(\tau _0)\ge 1$ and $\tau _1(\tau _0)>0$ so that

$$\begin{aligned} n>n_1(\tau _0)\text { and }\tau \in [0,\tau _1(\tau _0))\text { imply } C_{1}(\tau ^\alpha +n^{-\alpha })<\tau _0. \end{aligned}$$

So for $n>n_1$ and $\tau \in (0,\tau _1)$, by Theorem 1 (and the fact that $y\in {\mathcal {T}}^{\scriptscriptstyle {(n)}}_s$ for some $s\le \tau$ implies $(0,o)\overset{\varvec{a,n}}{\rightarrow }(s,y)$) we have

$$\begin{aligned} \tau \in (0,\delta _n]\text { implies }R_\tau ^{\scriptscriptstyle {(n)}}\subset B(o,C_{1}(\tau ^\alpha +n^{-\alpha }))\subset B(o,\tau _0). \end{aligned}$$

Therefore using (1.31) and (2.13) we see that for $n>n_1$ and $\tau \in (0,\tau _1)$,

$$\begin{aligned} {\mathbb {P}}(R^{\scriptscriptstyle {(n)}}_\tau \not \subset B(o,\tau _0)|S^{\scriptscriptstyle {(n)}}>s)&\le {\mathbb {P}}(\delta _n<\tau ,S^{\scriptscriptstyle {(n)}}>s)/{\mathbb {P}}(S^{\scriptscriptstyle {(n)}}>s)\\&\le \mu _n(\delta _n<\tau )/\mu _n(S^{\scriptscriptstyle {(n)}}>s)\\&\le C_{1}(\tau ^\beta +n^{-q})c_{1.21}(s\vee 1)({\underline{s}}_D)^{-1}\\&<\varepsilon , \end{aligned}$$

where the last inequality holds for $\tau$ sufficiently small and *n* sufficiently large, depending only on $\varepsilon$ and *s*. The result follows. $\square$

### Lemma 4.5

For every $$s>0$$,

$$\begin{aligned} {\mathbb {P}}_n^{s}\left( \bar{X}^{\scriptscriptstyle {(n)}}_\infty \in \cdot \right) \overset{w}{\longrightarrow }{\mathbb {N}}_o^s\left( \bar{X}^{}_\infty \in \cdot \right) \text { on }\mathcal {M}_F({\mathbb {R}}^d). \end{aligned}$$

### Proof

Fix $s>0$ and $\varepsilon >0$. Choose $t_\varepsilon >\max \{1/\varepsilon ,s\}$ and let $\varphi \in C_b(M_F({\mathbb {R}}^d))$. Then $\big |{\mathbb {P}}_n^{s}[\varphi (\bar{X}^{\scriptscriptstyle {(n)}}_\infty )]-{\mathbb {N}}_o^{s}[\varphi (\bar{X}_\infty )]\big |$ is at most

4.7 $$\begin{aligned}&\big |{\mathbb {P}}_n^{s}[\varphi (\bar{X}^{\scriptscriptstyle {(n)}}_\infty )\mathbb {1}_{\{S^{\scriptscriptstyle {(n)}}\le t_\varepsilon \}}]-{\mathbb {N}}_o^{s}[\varphi (\bar{X}^{}_\infty )\mathbb {1}_{\{S\le t_\varepsilon \}}]\big |\nonumber \\&\qquad +\big |{\mathbb {P}}_n^{s}[\varphi (\bar{X}^{\scriptscriptstyle {(n)}}_\infty )\mathbb {1}_{\{S^{\scriptscriptstyle {(n)}}>t_\varepsilon \}}]-{\mathbb {N}}_o^{s}[\varphi (\bar{X}^{}_\infty )\mathbb {1}_{\{S>t_\varepsilon \}}]\big |\nonumber \\&\quad \le \big |{\mathbb {P}}_n^{s}[\varphi ({\bar{X}}_{t_\varepsilon }^{\scriptscriptstyle {(n)}})]-{\mathbb {N}}_o^{s}[\varphi ({\bar{X}}_{t_\varepsilon })]\big | \end{aligned}$$

4.8 $$\begin{aligned}&\qquad +\big |{\mathbb {P}}_n^{s}[\varphi ({\bar{X}}_{t_\varepsilon }^{\scriptscriptstyle {(n)}})\mathbb {1}_{\{S^{\scriptscriptstyle {(n)}}> t_\varepsilon \}}]-{\mathbb {N}}_o^{s}[\varphi ({\bar{X}}_{t_\varepsilon })\mathbb {1}_{\{S>t_\varepsilon \}}]\big | \end{aligned}$$

4.9 $$\begin{aligned}&\qquad + \Vert \varphi \Vert _\infty ({\mathbb {P}}_n^{s}(S^{\scriptscriptstyle {(n)}}>t_\varepsilon )+{\mathbb {N}}_o^{s}(S>t_\varepsilon )). \end{aligned}$$

For *n* sufficiently large (depending on $s,t_\varepsilon ,\varepsilon$) the term (4.7) is less than $\varepsilon$ by Condition 6 (measure convergence). The quantity (4.8) is equal to

$$\begin{aligned} \left| {\mathbb {P}}_n^{t_\varepsilon }[\varphi ({\bar{X}}_{t_\varepsilon }^{\scriptscriptstyle {(n)}})]\frac{{\mathbb {P}}(S^{\scriptscriptstyle {(n)}}>t_\varepsilon )}{{\mathbb {P}}(S^{\scriptscriptstyle {(n)}}>s)}-{\mathbb {N}}_o^{t_\varepsilon }[\varphi ({\bar{X}}_{t_\varepsilon })]\frac{{\mathbb {N}}_0(S>t_\varepsilon )}{{\mathbb {N}}_o(S>s)}\right| . \end{aligned}$$

This is less than $\varepsilon$ for *n* sufficiently large depending on $s,t_\varepsilon ,\varepsilon$ by Condition 6 and Condition 1 (survival probability).

Similarly, (4.9) is equal to

$$\begin{aligned} \Vert \varphi \Vert _\infty \Big (\frac{{\mathbb {P}}(S^{\scriptscriptstyle {(n)}}>t_\varepsilon )}{{\mathbb {P}}(S^{\scriptscriptstyle {(n)}}>s)}+\frac{{\mathbb {N}}_o(S>t_\varepsilon )}{{\mathbb {N}}_o(S>s)}\Big ), \end{aligned}$$

which is less than or equal to $c \varepsilon$ for *n* sufficiently large (where *c* depends on *s* and $\Vert \varphi \Vert _\infty$) due to Condition 1 and (1.29). $\square$

Recalling Condition 7 (low density inequality), we set $K=1024$ and for $i\in {\mathbb {Z}}_+$, $\ell \in {\mathbb {N}}$ and $n\in [1,\infty ),$ define

$$\begin{aligned} \varOmega ^n_{i,\ell }&=\left\{ \omega : \exists x\in {\mathcal {T}}^{\scriptscriptstyle {(n)}}_{i2^{-\ell }}, \exists x'\text { s.t. }(i2^{-\ell },x)\overset{\varvec{a,n}}{\rightarrow }((i+1)2^{-\ell },x')\text { and }\right. \\&~~~~~~~~~~\quad \left. \int _{(i+1)2^{-\ell }}^{(i+2)2^{-\ell }}|\{y:(i2^{-\ell },x)\overset{\varvec{a,n}}{\rightarrow }(s,y)\}|ds/m(n)\le K^{-\ell }\right\} , \end{aligned}$$

and ${\hat{\varOmega }}_\ell ^n=\cup _{i=0}^{2^{2\ell }-1}\varOmega ^n_{i,\ell }$. The following result together with the modulus of continuity will ensure that, with high probability, points in the discrete range are near areas of significant integrated mass.

### Lemma 4.6

There is a $c_{4.6}>0$ and for any $n\ge 1$ there is an $M_n\in {\mathbb {N}}$ so that $M_n\uparrow \infty$ as $n\rightarrow \infty$, and if $\varOmega _m^n=\cup _{\ell =m}^{M_n}{\hat{\varOmega }}_\ell ^n$ for $m \in {\mathbb {N}}$, then

$$\begin{aligned} \mu _n(\varOmega _m^n)\le c_{4.6}2^{-m}\quad \text {for all }n\in [1,\infty ),\ m\in {\mathbb {N}}. \end{aligned}$$

### Proof

Let $\ell \in {\mathbb {N}}$, $i\in \{0,1,\dots ,2^{2\ell }-1\}$ and assume

4.10 $$\begin{aligned} n\ge K^{3\ell /2}. \end{aligned}$$

A simple change of variables ($u=ns$) in the time integral in the definition of $\varOmega ^n_{i,\ell }$ shows that

$$\begin{aligned} \mu _n(\varOmega ^n_{i,\ell })&\le m(n){\mathbb {E}}\,\,\Bigg [\sum _{x\in {\mathcal {T}}_{ni2^{-\ell }}}\mathbb {1}(\exists \,x'\text { s.t. }(ni2^{-\ell },x)\overset{\varvec{a}}{\rightarrow }(n(i+1)2^{-\ell },x'))\\&\quad \quad \quad \quad \quad \quad \times \mathbb {1}\Bigg (\int _{ni2^{-\ell }+n2^{-\ell }}^{ni2^{-\ell }+2n2^{-\ell }}|\{y:(ni2^{-\ell },x)\overset{\varvec{a}}{\rightarrow }(u,y)\}|du\le nm(n)K^{-\ell }\Bigg )\Bigg ]. \end{aligned}$$

So using Condition 7 with $\varDelta =n 2^{-\ell }\ge 4$ (by (4.10)) and $t=ni2^{-\ell }$ yields

4.11 $$\begin{aligned}&\mu _n(\varOmega ^n_{i,\ell })\nonumber \\&\quad \le c_{7}m(n){\mathbb {P}}\left( S^{\scriptscriptstyle (1)}>n2^{-\ell },\, \int _{n2^{-\ell }+2}^{2n2^{-\ell }-2}\frac{|{\mathcal {T}}_s|}{m(n2^{-\ell })n2^{-\ell }}ds\le \frac{m(n) K^{-\ell }}{m(n 2^{-\ell })2^{-\ell }}\right) \nonumber \\&\quad \le c_{7}m(n){\mathbb {P}}\left( S^{\scriptscriptstyle (n2^{-\ell })}>1,\, \int _{1+(2/(n2^{-\ell }))}^{2-(2/(n2^{-\ell }))}X_u^{(n2^{-\ell })}(1)du\le (K/2)^{-\ell }c_{1.21}2^{\ell }\right) , \end{aligned}$$

where in the last inequality we used (1.21), which applies because $n2^{-\ell }\ge 1$ by (4.10).

If $$I_{n,\ell }=[1,1+\frac{2^{\ell +1}}{n}]\cup [2-\frac{2^{\ell +1}}{n},2]$$, then

4.12 $$\begin{aligned}&{\mathbb {E}}\Bigg [\int \mathbb {1}(u\in I_{n,\ell })X_u^{(n2^{-\ell })}(1)du\,\Big |\,S^{\scriptscriptstyle (n2^{-\ell })}>1\Bigg ]\nonumber \\&\quad =\int _{I_{n,\ell }}{\mathbb {E}}\big [X_u^{(n2^{-\ell })}(1)\big ]du/{\mathbb {P}}(S^{\scriptscriptstyle (1)}>n2^{-\ell })\nonumber \\&\quad =\int _{I_{n,\ell }}\frac{{\mathbb {E}}[|{\mathcal {T}}_{un2^{-\ell }}|]}{m(n2^{-\ell }){\mathbb {P}}(S^{\scriptscriptstyle (1)}>n2^{-\ell })}du\le C_{4.12}\frac{2^\ell }{n}, \end{aligned}$$

where Condition 2 (total mass) and (1.22) are used in the last inequality.

Use the upper bound from (4.11), (1.21), and then (1.22) to conclude that

4.13 $$\begin{aligned}&\mu _n(\varOmega ^n_{i,\ell }) \nonumber \\&\quad \le c_{7}\frac{m(n)}{m(n2^{-\ell })}m(n2^{-\ell }){\mathbb {P}}(S^{\scriptscriptstyle (1)}>n2^{-\ell }) \nonumber \\&\qquad \times {\mathbb {P}}\left( \int _{1+(2/(n2^{-\ell }))}^{2-(2/(n2^{-\ell }))}X_u^{(n2^{-\ell })}(1)du\le c_{1.21}(K/4)^{-\ell }\Bigr |S^{\scriptscriptstyle (n2^{-\ell })}>1\right) \nonumber \\&\quad \le c_{7}c_{1.21}2^\ell \bar{s}_D{\mathbb {P}}\left( \int _{1+(2^{\ell +1}/n)}^{2-(2^{\ell +1}/n)} X_u^{(n2^{-\ell })}(1)du\le c_{1.21}(K/4)^{-\ell }\Bigr |S^{\scriptscriptstyle (n2^{-\ell })}>1\right) \nonumber \\&\quad \le C\Bigg [2^{\ell }{\mathbb {P}}\left( \int _1^2 X_u^{(n2^{-\ell })}(1)\,du\le 2c_{1.21}(K/4)^{-\ell }\Bigr |S^{\scriptscriptstyle (n2^{-\ell })}>1\right) \nonumber \\&\quad \quad \quad \quad +2^{\ell }{\mathbb {P}}\Bigg (\int _{I_{n,\ell }}X_u^{(n2^{-\ell })}(1)du>c_{1.21}(K/4)^{-\ell }\Bigr |S^{\scriptscriptstyle (n2^{-\ell })}>1\Bigg )\Bigg ]\nonumber \\&\quad :=C[T_1(\ell ,n)+T_2(\ell ,n)]. \end{aligned}$$

Markov’s inequality and (4.12) imply that

4.14 $$\begin{aligned} T_2(\ell ,n)\le \frac{C_{4.12}2^{2\ell }}{n}c_{1.21}^{-1}(K/4)^{\ell }:=C\frac{K^\ell }{n}\le C\,K^{-\ell /2},\ \forall n\ge K^{3\ell /2}. \end{aligned}$$

By Condition 6 (measure convergence) and the upper bound in (4.1),

$$\begin{aligned}&\limsup _{n\rightarrow \infty }\ 2^\ell {\mathbb {P}}\Bigl (\int _1^2 X_u^{(n2^{-\ell })}(1)du\le 2c_{1.21}(K/4)^{-\ell }\Bigr |S^{(n2^{-\ell })}>1\Bigr )\\&\quad \le 2^\ell {\mathbb {N}}_o\Bigg (\int _1^2X_u(1)du\le 2 c_{1.21}(K/4)^{-\ell }\Bigr |S>1\Bigg )\\&\quad \le (C/2)(\sqrt{K}/4)^{-\ell }. \end{aligned}$$

Therefore there is an increasing sequence $\{n(\ell ):\ell \in {\mathbb {N}}\}$ such that $n(\ell )\ge K^{3\ell /2}$ and

4.15 $$\begin{aligned} T_1(\ell ,n)\le C(\sqrt{K}/4)^{-\ell }\quad \text { for all }n\ge n(\ell ). \end{aligned}$$

Use (4.14) and (4.15) in (4.13) and sum over $i=0,\dots , 2^{2\ell }-1$ to deduce that

4.16 $$\begin{aligned} \mu _n({\hat{\varOmega }}_\ell ^n)\le C(\sqrt{K}/16)^{-\ell }=C2^{-\ell }\quad \text {for all }n\ge n(\ell ). \end{aligned}$$

For $n\ge 1$, define $M_n=\max \{\ell :n(\ell )\le n\}\uparrow \infty$ as $n\rightarrow \infty$ ($\max \varnothing =0$). Then by (4.16) for any $m\in {\mathbb {N}}$,

$$\begin{aligned} \mu _n(\varOmega _m^n)\le \sum _{\ell =m}^{M_n}\mu _n({\hat{\varOmega }}_\ell ^n)\le \sum _{\ell =m}^{M_n}C2^{-\ell }\le 2C2^{-m}, \end{aligned}$$

because $\ell \le M_n$ implies that $n(\ell )\le n$. $\square$

*Proof of Theorem* 2. Recall (1.5). Let

$$\begin{aligned} {\tilde{e}}^{\scriptscriptstyle {(n)}}=\big (({{{\hat{e}}}}^{\scriptscriptstyle {(n)}}_t(y/\sqrt{n},x/\sqrt{n}))_{t \ge 0}:y,x\in {\mathbb {Z}}^d)\in \big (\mathcal {D}([0,\infty ),\mathcal {D}_{{\mathbb {R}}})\big )^{{\mathbb {Z}}^d\times {\mathbb {Z}}^d}:=\tilde{\mathcal {D}}, \end{aligned}$$

where (1.11) is used to show that, using the usual countable product metric,

$$\begin{aligned} {\tilde{e}}^{\scriptscriptstyle {(n)}}&\text { belongs to the complete separable metric space }\tilde{\mathcal {D}}. \end{aligned}$$

For $s>0$, the joint probability law of $(\varvec{X}^{\scriptscriptstyle {(n)}},\tilde{e}^{\scriptscriptstyle {(n)}})$ (on $\mathcal {D}\times \tilde{\mathcal {D}}$) conditional on $S^{\scriptscriptstyle {(n)}}>s$ is written as

$$\begin{aligned}&\mu ^s_n((\varvec{X}^{\scriptscriptstyle {(n)}},{\tilde{e}}^{\scriptscriptstyle {(n)}})\in \cdot )\\&\quad =\mu _n((\varvec{X}^{\scriptscriptstyle {(n)}},{\tilde{e}}^{\scriptscriptstyle {(n)}})\in \cdot |S^{\scriptscriptstyle {(n)}}>s). \end{aligned}$$

Although $\mu ^s_n={\mathbb {P}}^s_n$, we will soon be trading in our familiar ${\mathbb {P}}$ to apply Skorokhod’s Theorem and so to avoid confusion it will be useful to work with $\mu ^s_n$ on our original probability space.

Fix $s>0$. It suffices to show weak convergence along any sequence $n_k\rightarrow \infty$ and to ease the notation we will simply assume $n\in {\mathbb {N}}$ (the proof being the same in the general case). By Lemma 4.5 and the Skorokhod Representation Theorem we may work on some $(\varOmega ,\mathcal {F},{\mathbb {P}}^{\scriptscriptstyle \{s\}})$ on which there are random measures $(\bar{X}^{\scriptscriptstyle {(n)}}_\infty )_{n\in {\mathbb {N}}}$ and $\bar{X}^{}_\infty$ such that $\bar{X}^{\scriptscriptstyle {(n)}}_\infty$ has law $\mu _n^s(\bar{X}^{\scriptscriptstyle {(n)}}_\infty \in \cdot )$ for each *n*, $\bar{X}^{}_\infty$ has law ${\mathbb {N}}_o^s(\bar{X}^{}_\infty \in \cdot )$, and

4.17 $$\begin{aligned} \bar{X}^{\scriptscriptstyle {(n)}}_\infty \overset{a.s.}{\longrightarrow }\bar{X}^{}_\infty \quad \text { as }n\rightarrow \infty . \end{aligned}$$

For now we may assume $\mathcal {F}=\sigma ((\bar{X}^{\scriptscriptstyle {(n)}}_\infty )_{n\in {\mathbb {N}}}, \bar{X}^{}_\infty )$. We claim that we may assume that for all $n\in {\mathbb {N}}$ there are processes $(\varvec{X}^{\scriptscriptstyle {(n)}},{\tilde{e}}^{\scriptscriptstyle {(n)}})\in \mathcal {D}\times \tilde{\mathcal {D}}$ defined on $(\varOmega ,\mathcal {F},{\mathbb {P}}^{\scriptscriptstyle \{s\}})$ such that

$$\begin{aligned}&\forall n\in {\mathbb {N}}\ \text { the law of }(\varvec{X}^{\scriptscriptstyle {(n)}},\tilde{e}^{\scriptscriptstyle {(n)}})\text { on }\mathcal {D}\times \tilde{\mathcal {D}}\text { is }\mu _n^s((\varvec{X}^{\scriptscriptstyle {(n)}},{\tilde{e}}^{\scriptscriptstyle {(n)}})\in \cdot )\\&\quad \text { and }\bar{X}^{\scriptscriptstyle {(n)}}_\infty =\int _0^\infty X_u^{\scriptscriptstyle {(n)}}\,du\ \text { a.s.} \end{aligned}$$

To see this, work on ${{\bar{\varOmega }}}=\varOmega \times \mathcal {D}\times \tilde{\mathcal {D}}$ with the product $\sigma$-field $\bar{\mathcal {F}}$, and define ${{\bar{{\mathbb {P}}}}}^{\{s\}}$ on $\bar{\mathcal {F}}$ by

$$\begin{aligned}&{{\bar{{\mathbb {P}}}}}^{\{s\}} \left( (\bar{X}^{}_\infty ,(\bar{X}^{\scriptscriptstyle {(n)}}_\infty )_{n\in {\mathbb {N}}})\in A, (\varvec{X}^{\scriptscriptstyle {(n)}},{\tilde{e}}^{\scriptscriptstyle {(n)}})_{n\le N}\in \prod _{n=1}^N B_n\right) \\&\quad =\int \mathbb {1}_A(\bar{X}^{}_\infty ,(\bar{X}^{\scriptscriptstyle {(n)}}_\infty )_{n\in {\mathbb {N}}})\prod _{n=1}^N\mu _n^s((\varvec{X}^{\scriptscriptstyle {(n)}},\tilde{e}^{\scriptscriptstyle {(n)}})\in B_n|\bar{X}^{\scriptscriptstyle {(n)}}_\infty )d{\mathbb {P}}^{\scriptscriptstyle \{s\}}, \end{aligned}$$

where the above conditional probabilities are taken to be a regular conditional probabilities. In short, given $(\bar{X}^{}_\infty ,(\bar{X}^{\scriptscriptstyle {(n)}}_\infty )_{n\in {\mathbb {N}}})$, we take $(\varvec{X}^{\scriptscriptstyle {(n)}},\tilde{e}^{\scriptscriptstyle {(n)}})_{n\in {\mathbb {N}}}$ to be conditionally independent and with laws $\mu _n^s((\varvec{X}^{\scriptscriptstyle {(n)}},{\tilde{e}}^{\scriptscriptstyle {(n)}})\in \cdot |\bar{X}^{\scriptscriptstyle {(n)}}_\infty )$. Clearly the resulting enlarged space satisfies the above claim. We now relabel the enlarged space as $(\varOmega ,\mathcal {F},{\mathbb {P}}^{\scriptscriptstyle \{s\}})$ to ease the notation.

Note that for each fixed *n*, $\varvec{{\mathcal {T}}}^{\scriptscriptstyle {(n)}}=({\mathcal {T}}^{\scriptscriptstyle {(n)}}_t)_{t\ge 0}:=(\text {supp}(X^{\scriptscriptstyle {(n)}}_t))_{t\ge 0}$ is a copy of our rescaled set-valued process so we can define $S^{\scriptscriptstyle {(n)}}, R^{\scriptscriptstyle {(n)}}$ and $\overset{\varvec{a,n}}{\rightarrow }$ just as before using ${\tilde{e}}^{\scriptscriptstyle {(n)}}$ and $\varvec{{\mathcal {T}}}^{\scriptscriptstyle {(n)}}$. For the latter, set $e^{\scriptscriptstyle {(n)}}_{s,t}(y,x)={\tilde{e}}^{\scriptscriptstyle {(n)}}_t(y,x)(s)$ and write $(s,u)\overset{\varvec{a,n}}{\rightarrow }(t,x)$ iff $s\le t$ and $e^{\scriptscriptstyle {(n)}}_{s,t}(x,y)=1$ (recall (1.5) and (1.17)). In particular $\varOmega ^n_{i,\ell }$ and $\varOmega ^n_m$, as in Lemma 4.6 can be defined as subsets of $\varOmega$, and that result and (1.31) imply

4.18 $$\begin{aligned} {\mathbb {P}}^{\scriptscriptstyle \{s\}}(\varOmega ^n_m)=\mu _n^s(\varOmega _m^n) \le \frac{\mu _n(\varOmega _m^n)}{\mu _n(S^{\scriptscriptstyle {(n)}}>s)}&\le c_{4.6}2^{-m}/\mu _n(S^{\scriptscriptstyle {(n)}}>s) \nonumber \\&\le \frac{c_{4.6}c_{1.21}}{{\underline{s}}_D}(s\vee 1)2^{-m}:=c_{4.18}(s)2^{-m}. \end{aligned}$$

To reinterpret Theorem 1 we introduce

4.19 $$\begin{aligned} \varDelta ^{\scriptscriptstyle {(n)}}(\rho )=\sup \{|y_2-y_1|:\ |s_2-s_1|\le \rho ,\ (s_1,y_1)\overset{\varvec{a,n}}{\rightarrow }(s_2,y_2)\}, \end{aligned}$$

and note that the law of $\varDelta ^{\scriptscriptstyle {(n)}}(\rho )$ is the same under ${\mathbb {P}}^{\scriptscriptstyle \{s\}}$ and $\mu _n^s$. Therefore

$$\begin{aligned} {\mathbb {P}}^{\scriptscriptstyle \{s\}}(\varDelta ^{\scriptscriptstyle {(n)}}(\rho )>C_{1}(\rho ^\alpha +n^{-\alpha }))\le \frac{\mu _n^s(\varDelta ^{\scriptscriptstyle {(n)}}(\rho )>C_{1}(\rho ^\alpha +n^{-\alpha }))}{\mu _n(S^{\scriptscriptstyle {(n)}}>s)}. \end{aligned}$$

On the original space $\varDelta ^{\scriptscriptstyle {(n)}}(\rho )>C_{1}(\rho ^\alpha +n^{-\alpha })$ implies $\delta _n<\rho$ and so by Theorem 1 and (1.31) (as used in (4.18))

4.20 $$\begin{aligned} \forall \rho \in [0,1],\ \ {\mathbb {P}}^{\scriptscriptstyle \{s\}}(\varDelta ^{\scriptscriptstyle {(n)}}(\rho )>C_{1}(\rho ^\alpha +n^{-\alpha }))&\le \mu _n(\delta _n<\rho )/\mu _n(S^{\scriptscriptstyle {(n)}}>s) \nonumber \\&\le c_{4.20}(s) [\rho ^\beta +n^{-q}]. \end{aligned}$$

Next note that (1.31) implies for $m\in {\mathbb {N}}$,

4.21 $$\begin{aligned} {\mathbb {P}}^{\scriptscriptstyle \{s\}}(S^{\scriptscriptstyle {(n)}}>2^{m-1})\le \frac{\mu _n(S^{\scriptscriptstyle {(n)}}>2^{m-1})}{\mu _n(S^{\scriptscriptstyle {(n)}}>s)}\le \frac{c_{4.21}(s)}{2^{m-1}\wedge n}. \end{aligned}$$

Finally use (1.31) and recall Lemma 4.4(b) to see that for $m\in {\mathbb {N}}$,

4.22 $$\begin{aligned} {\mathbb {P}}^{\scriptscriptstyle \{s\}}(r_0(R^{\scriptscriptstyle {(n)}})\ge 2^{m-1})\le \frac{\mu _n(r_0(R^{\scriptscriptstyle {(n)}})\ge 2^{m-1})}{\mu _n(S^{\scriptscriptstyle {(n)}}>s)}\le c_{4.22}(s)\eta _{4.4}(2^{m-1}). \end{aligned}$$

We have $R^{\scriptscriptstyle {(n)}}=\mathcal {R}(\varvec{X}^{\scriptscriptstyle {(n)}})={\mathrm {supp}}(\bar{X}^{\scriptscriptstyle {(n)}}_\infty )$, and $R={\mathrm {supp}}(\bar{X}^{}_\infty )$ defines the range of a SBM. It suffices to show

$$\begin{aligned} d_0(R^{\scriptscriptstyle {(n)}},R)\overset{{\mathbb {P}}^{\scriptscriptstyle \{s\}}}{\longrightarrow } 0 \quad \text { as }n\rightarrow \infty . \end{aligned}$$

Lemma 4.3 and (4.17) show that $\varDelta _1(R,R^{\scriptscriptstyle {(n)}})\overset{a.s.}{\longrightarrow }0$, and therefore if we set $R^\delta :=\{x:d(x,R)\le \delta \}$ it suffices to fix $\tau _0>0$ and prove

4.23 $$\begin{aligned} \lim _{n\rightarrow \infty }{\mathbb {P}}^{\scriptscriptstyle \{s\}}(R^{\scriptscriptstyle {(n)}}\not \subset R^{\tau _0})=0. \end{aligned}$$

Fix $\varepsilon >0$ and let $\tau (\tau _0,\varepsilon ,s)$, $n_0(\tau _0,\varepsilon ,s)$ be as in Lemma 4.4(c), and choose $0<\tau <\tau (\tau _0,\varepsilon ,s)$. Recalling $R^{\scriptscriptstyle {(n)}}_\tau =\cup _{s\le \tau }{\mathcal {T}}_s^{\scriptscriptstyle {(n)}}$, we see from Lemmas 4.2 and 4.4(c) that

4.24 $$\begin{aligned} {\mathbb {P}}^{\scriptscriptstyle \{s\}}(R_\tau ^{\scriptscriptstyle {(n)}}\not \subset R^{\tau _0})\le {\mathbb {P}}^{\scriptscriptstyle \{s\}}(R_\tau ^{\scriptscriptstyle {(n)}}\not \subset B(0,\tau _0))=&\,\mu ^s_n(R_\tau ^{\scriptscriptstyle {(n)}}\not \subset B(0,\tau _0))\le \varepsilon \nonumber \\&\text { for }n\ge n_0(\tau _0,\varepsilon ,s). \end{aligned}$$

For $m\in {\mathbb {N}}$, define the finite grid of points

$$\begin{aligned} G_m= \Bigl \{\frac{\mathbf {i}2^{-m}}{K_0}:\mathbf {i}\in {\mathbb {Z}}^d\Bigr \}\cap [-2^m,2^m]^d, \end{aligned}$$

where $K_0\in {\mathbb {N}}$ is chosen so that

4.25 $$\begin{aligned} \forall x\in B(o,2^{-m})\ \exists q\in G_m\text { so that }|q-x|\le 2^{-m}. \end{aligned}$$

Define the finite collection

$$\begin{aligned} \mathcal {B}_m=\{B(x,\tau _0/2):\,x\in G_m\}. \end{aligned}$$

Fix $m\in {\mathbb {N}}$ sufficiently large so that

4.26 $$\begin{aligned}&(i)\ 2^{-m}+2C_{1}2^{(1-m)\alpha }<\tau _0/10, \end{aligned}$$

4.27 $$\begin{aligned}&(ii)\ 2^{1-m}<\tau , \end{aligned}$$

4.28 $$\begin{aligned}&(iii)\ c_{4.18}(s)2^{-m}+c_{4.20}(s)2^{(1-m)\beta } +\frac{c_{4.21}(s)}{2^{m-1}}\nonumber \\&\qquad \qquad \qquad +c_{4.22}(s)\eta _{4.3}(2^{m-1})<\varepsilon . \end{aligned}$$

If $(W_u,u\ge 0)$ denotes a *d*-dimensional Brownian motion with variance parameter $\sigma _0^2$ starting at *o* under a probability measure ${\mathbb {P}}_o$, then for any open ball *B*,

$$\begin{aligned} {\mathbb {E}}^{\scriptscriptstyle \{s\}}[\bar{X}^{}_\infty (\partial B)]={\mathbb {N}}_o^s[\bar{X}^{}_\infty (\partial B)]&\le \int _0^\infty {\mathbb {N}}_o[X_u(\partial B)]\,du/{\mathbb {N}}_o(S>s)\\&=\int _0^\infty {\mathbb {P}}_o(W_u\in \partial B)\,du/{\mathbb {N}}_o(S>s)\\&=0, \end{aligned}$$

where the second line is standard (e.g. Theorem II.7.2(iii) of [32]). Therefore by (4.17), the above equality, and standard properties of the weak topology we have (recall $K=1024$)

$$\begin{aligned} \lim _{n\rightarrow \infty }{\mathbb {P}}^{\scriptscriptstyle \{s\}}\Bigg (\sup _{B\in \mathcal {B}_m}|\bar{X}^{\scriptscriptstyle {(n)}}_\infty (B)-\bar{X}^{}_\infty (B)|\ge \frac{1}{2}K^{-m}\Bigg )=0. \end{aligned}$$

So there is an $n_1=n_1(m,\varepsilon ,\tau _0,s)$ so that for $n\ge n_1$ we have

4.29 $$\begin{aligned} {\mathbb {P}}^{\scriptscriptstyle \{s\}}\Big (\sup _{B\in \mathcal {B}_m}|\bar{X}^{\scriptscriptstyle {(n)}}_\infty (B)-\bar{X}^{}_\infty (B)|\ge \frac{1}{2}K^{-m}\Big )<\varepsilon , \end{aligned}$$

and, if $M_n$ is as in Lemma 4.6, then for $n\ge n_1$

4.30 $$\begin{aligned}&(i)\ m\le M_n, \end{aligned}$$

4.31 $$\begin{aligned}&(ii)\ 2C_{1}n^{-\alpha }<\tau _0/10 , \end{aligned}$$

4.32 $$\begin{aligned}&(iii)\ n>2^{m-1}\text { and } [c_{4.20}(s)+c_{4.21}(s)]n^{-q}<\varepsilon . \end{aligned}$$

Assume $n\ge n_1$, and then choose $\omega$ so that ($\varDelta ^{\scriptscriptstyle (n)}$ is as in (4.19))

4.33 $$\begin{aligned}&(i)\ \omega \in (\varOmega ^n_m)^c,\quad (ii)\ \varDelta ^{\scriptscriptstyle {(n)}}(2^{1-m})\le C_{1}(2^{(1-m)\alpha }+n^{-\alpha }), \end{aligned}$$

4.34 $$\begin{aligned}&(iii)\ S^{\scriptscriptstyle {(n)}}\le 2^{m-1}, \quad (iv)\ r_0(R^{\scriptscriptstyle {(n)}})<2^{m-1}, \end{aligned}$$

4.35 $$\begin{aligned}&(v)\ \sup _{B\in \mathcal {B}_m}|\bar{X}^{\scriptscriptstyle {(n)}}_\infty (B)-\bar{X}^{}_\infty (B)|< \frac{1}{2}K^{-m}. \end{aligned}$$

Let $x\in R^{\scriptscriptstyle {(n)}}{\setminus } R_\tau ^{\scriptscriptstyle {(n)}}$, and then choose $s>\tau$ so that $x\in {\mathcal {T}}_s^{\scriptscriptstyle {(n)}}$. By (4.27), (iii) in (4.34), and $s<S^{\scriptscriptstyle {(n)}}$ (since ${\mathcal {T}}_s^{\scriptscriptstyle {(n)}}$ is non-empty), we have

$$\begin{aligned} 2^{1-m}<\tau<s<S^{\scriptscriptstyle {(n)}}\le 2^{m-1}, \end{aligned}$$

and so we can choose $i\in \{0,\dots ,2^{2m-1}\}$ so that $s\in [(i+1)2^{-m},(i+2)2^{-m})$. Since $|x|\le r_0(R^{\scriptscriptstyle {(n)}})<2^{m-1}$ (by (iv) of (4.34)), (4.25) shows there is a $q\in G_m$ so that

4.36 $$\begin{aligned} |x-q|\le 2^{-m}<\tau _0/10, \end{aligned}$$

the last by (4.26). By (1.10) $\exists \, x_0\in {\mathcal {T}}^{\scriptscriptstyle {(n)}}_{i2^{-m}}$ such that $(i2^{-m},x_0)\overset{\varvec{a,n}}{\rightarrow }(s,x)$. Assume $u\in [(i+1)2^{-m},(i+2)2^{-m}]$ and $(i2^{-m},x_0)\overset{\varvec{a,n}}{\rightarrow }(u,y)$ for some $y\in {\mathcal {T}}^{\scriptscriptstyle {(n)}}_u$. Then use (4.36), (4.19), and (ii) in (4.33) to see that

$$\begin{aligned} |y-q|&\le |y-x_0|+|x_0-x|+|x-q|\\&\le 2\varDelta ^{\scriptscriptstyle {(n)}}(2^{1-m})+\tau _0/10\\&\le 2C_{1}(2^{(1-m)\alpha }+n^{-\alpha })+\tau _0/10\\&\le 3\tau _0/10<\tau _0/2, \end{aligned}$$

where we have used (4.26) and (4.31) in the last line. This implies that

4.37 $$\begin{aligned} \bar{X}^{\scriptscriptstyle {(n)}}_\infty (B(q,\tau _0/2))&\ge \int _{(i+1)2^{-m}}^{(i+2)2^{-m}} X_u^{\scriptscriptstyle {(n)}}(B(q,\tau _0/2))\,du\nonumber \\&\ge \int _{(i+1)2^{-m}}^{(i+2)2^{-m}} X_u^{\scriptscriptstyle {(n)}}(\{y\in {\mathcal {T}}^{\scriptscriptstyle {(n)}}_u:\,(i 2^{-m},x_0)\overset{\varvec{a,n}}{\rightarrow }(u,y)\})\,du. \end{aligned}$$

By (1.10) and $(i2^{-m},x_0)\overset{\varvec{a,n}}{\rightarrow }(u,y)$, there is an $x'\in {\mathcal {T}}_{(i+1)2^{-m}}$ s.t.

$$\begin{aligned} (i2^{-m},x_0)\overset{\varvec{a,n}}{\rightarrow }((i+1)2^{-m},x')\overset{\varvec{a,n}}{\rightarrow }(u,y). \end{aligned}$$

The fact that $\omega \notin \varOmega _m^n$ (by (i) of (4.33)), $m\le M_n$ (by (4.30)), $i\in \{0\dots ,2^{2m-1}\}$, $x_0\in {\mathcal {T}}_{i2^{-m}}^{\scriptscriptstyle {(n)}}$, and $(i2^{-m},x_0)\overset{\varvec{a,n}}{\rightarrow }((i+1)2^{-m},x')$ shows that the right-hand side of (4.37) is at least $K^{-m}$. This inequality and (4.35) imply

$$\begin{aligned} \bar{X}^{}_\infty (B(q,\tau _0/2))>K^{-m}/2. \end{aligned}$$

Use this and (4.36) to conclude $\bar{X}^{}_\infty (B(x,\tau _0))>K^{-m}/2$ and therefore that $x\in R^{\tau _0}$. We have shown that

4.38 $$\begin{aligned} \text {For }n\ge n_1, \text { conditions}(4.33)-(4.35)\text { imply that }(R^{\scriptscriptstyle {(n)}}{\setminus } R^{\scriptscriptstyle {(n)}}_\tau )\subset R^{\tau _0}. \end{aligned}$$

Now use (4.18), (4.20), (4.21), (4.22), and (4.29) to see that the probability, *P*(*m*, *n*) that one of the 5 conditions listed in (4.33)-(4.35) fails is at most

$$\begin{aligned}&{\mathbb {P}}^{\scriptscriptstyle \{s\}}(\varOmega _m^n)+{\mathbb {P}}^{\scriptscriptstyle \{s\}}(\varDelta ^{\scriptscriptstyle {(n)}}(2^{1-m})>C_{1}(2^{(1-m)\alpha }+n^{-\alpha }))\\&\quad \quad +{\mathbb {P}}^{\scriptscriptstyle \{s\}}(S^{\scriptscriptstyle {(n)}}>2^{m-1})+{\mathbb {P}}^{\scriptscriptstyle \{s\}}(r_0(R^{\scriptscriptstyle {(n)}})\ge 2^{m-1})+\varepsilon \\&\quad \le c_{4.18}(s)2^{-m}+c_{4.20}(s)[2^{(1-m)\beta }+n^{-q}]+\frac{c_{4.21}(s)}{2^{m-1}\wedge n} \\&\quad \quad +c_{4.22}(s)\eta _{4.4}(2^{m-1})+\varepsilon . \end{aligned}$$

Our lower bounds on *m* and *n* (in particular use (4.28) and (4.32)) shows that the above is at most $3\varepsilon$, and so we have shown

$$\begin{aligned} P(m,n)<3\varepsilon \text { for }n>n_1(m,\varepsilon ,\tau _0) \text { and where }m \text { is chosen as above}. \end{aligned}$$

Recalling (4.38) we conclude that

$$\begin{aligned} {\mathbb {P}}^{\scriptscriptstyle \{s\}}((R^{\scriptscriptstyle {(n)}}{\setminus } R_\tau ^{\scriptscriptstyle {(n)}})\not \subset R^{\tau _0})<3\varepsilon \text { for }n>n_1. \end{aligned}$$

This, together with (4.24), proves (4.23) and so completes the proof. $\square$

## The extrinsic one-arm probability

In this section we prove Theorem 3. Recall that $r_0(G)=\sup \{|x|: x\in G\}$.

### Lemma 5.1

The map $r_0: \mathcal {K} \rightarrow [0,\infty )$ is continuous.

### Proof

Let $K_n \rightarrow K$, and let $\varepsilon >0$. Choose $n_0$ sufficiently large so that $K_n \subset K^\varepsilon$ and $K\subset K_n^\varepsilon$ for all $n\ge n_0$. Then for such *n*, $r_0(K_n)\le r_0(K)+\varepsilon$ and $r_0(K)\le r_0(K_n)+\varepsilon$. $\square$

### Proof of Theorem 3

Let $$\varepsilon \in (0,1)$$, $$\alpha ,\beta $$ be as in (2.9) and (2.10), and let $$\delta _n$$, $$C_{1}$$ be as in Theorem 1. $${\mathbb {N}}_o$$ is the canonical measure for the $$(\gamma , \sigma _0^2)$$-SBM arising in Theorem 2. By Lemma 1.9 we have $${\mathbb {N}}_o(r_0(R)>1)<\infty $$, and as $$S>0$$$${\mathbb {N}}_o$$-a.e., we may choose $$s\in (0,1)$$ small enough so that

5.1 $$\begin{aligned} {\mathbb {N}}_o(r_0(R)>1,S\le s)<\varepsilon , \end{aligned}$$

and

5.2 $$\begin{aligned} C_{1}(s^\alpha +s^\beta )<\varepsilon /2. \end{aligned}$$

Assume $n_1\ge 1$ is large enough so that

5.3 $$\begin{aligned} C_{1}(n_1^{-\alpha }+n_1^{-q})<\varepsilon /2, \end{aligned}$$

and

5.4 $$\begin{aligned} |\mu _n(S^{\scriptscriptstyle {(n)}}>s)-(s_D/s)|<\varepsilon \quad \forall n\ge n_1, \end{aligned}$$

where (1.30) is used for the last.

Assume that $n\ge n_1$ and $S^{\scriptscriptstyle {(n)}}\le s\le \delta _n$. Then for any $x\in {\mathcal {T}}^{\scriptscriptstyle {(n)}}_t$ we must have $t\le S^{\scriptscriptstyle {(n)}}\le s\le \delta _n$, and so by Theorem 1 and $(0,o)\overset{\varvec{a,n}}{\rightarrow }(t,x)$,

$$\begin{aligned} |x|=|x-o|\le C_{1}(t^\alpha +n^{-\alpha })\le C_{1}(s^\alpha +n^{-\alpha })<\varepsilon , \end{aligned}$$

the last inequality by (5.2) and (5.3). This proves that for all $n\ge n_1$,

$$\begin{aligned} S^{\scriptscriptstyle {(n)}}\le s\le \delta _n\Rightarrow r_0(R^{\scriptscriptstyle {(n)}})\le \varepsilon <1. \end{aligned}$$

Therefore for $n\ge n_1$, we have by Theorem 1,

5.5 $$\begin{aligned} \mu _n(r_0(R^{\scriptscriptstyle {(n)}})>1,S^{\scriptscriptstyle {(n)}}\le s)\le \mu _n(\delta _n<s)\le C_{1}(s^\beta +n^{-q})<\varepsilon , \end{aligned}$$

the last by (5.2) and (5.3) again.

If $D_c(h)$ denotes the set of discontinuity points of $h:\mathcal {K}\rightarrow {\mathbb {R}}$, then by Lemma 5.1,

$$\begin{aligned} {\mathbb {N}}_o(R\in D_c(\mathbb {1}_{\{r_0>1\}}))\le {\mathbb {N}}_o(r_0(R)=1)=0, \end{aligned}$$

where we have used Lemma 1.9 in the last equality. So Theorem 2 shows that we may also assume that $n_1$ is large enough so that

5.6 $$\begin{aligned} |{\mathbb {P}}_n^s(r_0(R^{\scriptscriptstyle {(n)}})>1)-{\mathbb {N}}_o^s(r_0(R)>1)|<\varepsilon \quad \text { for }n\ge n_1. \end{aligned}$$

Write $O(\varepsilon )$ for any quantity bounded in absolute value by $C\varepsilon$ for some constant *C* independent of *n*. Then for $n\ge n_1$ we have

5.7 $$\begin{aligned} m(n){\mathbb {P}}(r_0(R^{\scriptscriptstyle (1)})>\sqrt{n})&=\mu _n(r_0(R^{\scriptscriptstyle {(n)}})>1) \nonumber \\&=\mu _n(r_0(R^{\scriptscriptstyle {(n)}})>1,S^{\scriptscriptstyle {(n)}}>s)+O(\varepsilon )\quad (\text {by }(5.5)) \nonumber \\&={\mathbb {P}}_n^s(r_0(R^{\scriptscriptstyle {(n)}})>1)\mu _n(S^{\scriptscriptstyle {(n)}}>s)+O(\varepsilon )\nonumber \\&={\mathbb {N}}_o^s(r_0(R)>1)\mu _n(S^{\scriptscriptstyle {(n)}}>s)+O(\varepsilon )\ \ (\text {by }(5.6), (1.31)) \nonumber \\&={\mathbb {N}}_o^s(r_0(R)>1)\frac{s_D}{s}+O(\varepsilon )\quad (\text {by }(5.4)). \end{aligned}$$

Next use (5.1) and ${\mathbb {N}}_o(S>s)=\frac{2}{\gamma s}$ to see that

5.8 $$\begin{aligned} {\mathbb {N}}_o^s(r_0(R)>1)\frac{s_D}{s}&=\frac{{\mathbb {N}}_o(r_0(R)>1,S>s)}{2/(\gamma s)}\frac{s_D}{s} \nonumber \\&={\mathbb {N}}_o(r_0(R)>1)\frac{\gamma s_D}{2}+O(\varepsilon ) \nonumber \\&=\frac{ \sigma _0^2}{2}s_Dv_d(0)+O(\varepsilon ), \end{aligned}$$

where Lemma 1.9 is used in the final equality. Combining (5.7) with (5.8), we see that

$$\begin{aligned} \lim _{n\rightarrow \infty }m(n){\mathbb {P}}(r_0(R^{\scriptscriptstyle (1)})>\sqrt{n})=\frac{ \sigma _0^2}{2}s_Dv_d(0), \end{aligned}$$

which gives (2.18) in Theorem 3. (2.17) then follows from this and (1.30). $\square$

## On checking conditions 6–7 and the existence of ancestral paths

Here we prove Lemmas 2.2 and 2.3 as well as Propositions 1.5 and 2.4.

### Proof of Lemma 2.2

To prove Condition 6 (measure convergence), it suffices to prove convergence along any sequence. To simplify notation we assume $n\in {\mathbb {N}}$ and that the branching and diffusion parameters of the limiting super-Brownian motion are both one. Fix $0\le t_0<t_1$. Let $p\in {\mathbb {N}}$ and $u_1,\dots ,u_p\in [t_0,t_1]$. Let $\phi \ge 0$ be in $C_b({\mathbb {R}}^d)$. Then by (2.5),

6.1 $$\begin{aligned} {\mathbb {P}}^s_n\left[ \Bigl (\prod _{i=1}^pX^{\scriptscriptstyle {(n)}}_{u_i}(\phi )\Bigr )^2\right] \le \Vert \phi \Vert _\infty ^{2p}C(s,t_1,p)\quad \forall n\in {\mathbb {N}}. \end{aligned}$$

It follows easily from the finite-dimensional convergence (2.4) and the above $L^2$ bound (e.g. use Skorokhod’s representation to get a.s. convergence in (2.4)) that

6.2 $$\begin{aligned} \lim _{n\rightarrow \infty }{\mathbb {P}}_n^s\left[ \prod _{i=1}^pX^{\scriptscriptstyle {(n)}}_{u_i}(\phi )\right] ={\mathbb {N}}_o^s\Big [\prod _{i=1}^p X_{u_i}(\phi )\Big ]. \end{aligned}$$

Using Fubini’s theorem we have

6.3 $$\begin{aligned}&\lim _{n\rightarrow \infty } {\mathbb {P}}_n^s\left[ \Bigg (\int _{t_0}^{t_1}X^{\scriptscriptstyle {(n)}}_u(\phi )du\Bigg )^p\right] \nonumber \\&\quad =\lim _{n\rightarrow \infty }\int _{t_0}^{t_1}\dots \int _{t_0}^{t_1}{\mathbb {P}}_n^s\Bigg [\prod _{i=1}^pX^{\scriptscriptstyle {(n)}}_{u_i}(\phi )\Bigg ]du_1\dots du_p \nonumber \\&\quad =\int _{t_0}^{t_1}\dots \int _{t_0}^{t_1}{\mathbb {N}}_o^s\Bigg [\prod _{i=1}^p X_{u_i}(\phi )\Bigg ]du_1\dots du_p \nonumber \\&\quad ={\mathbb {N}}_o^s\left[ \Bigg (\int _{t_0}^{t_1} X_u(\phi )du\Bigg )^p\right] <\infty , \end{aligned}$$

where (6.2), (6.1) and dominated convergence are used in the second equality. If *W* denotes a standard Brownian motion starting at *o* under $P_o$, then take $p=1$ in the above to see that

$$\begin{aligned} \lim _{n\rightarrow \infty }{\mathbb {P}}_n^s\Bigg [({{\bar{X}}}_{t_1}^{\scriptscriptstyle {(n)}}-\bar{X}_{t_0}^{\scriptscriptstyle {(n)}})(\phi )\Bigg ]&={\mathbb {N}}_o^s\Big [\int _{t_0}^{t_1}X_u(\phi )du\Big ]\\&\le E_o\Big [\int _{t_0}^{t_1}\phi (W_u)du\Big ]/{\mathbb {N}}_o(\mathcal {S}>s), \end{aligned}$$

where the last is because the mean measure of $X_t$ under ${\mathbb {N}}_o$ is $P_o(B_t\in \cdot )$ (e.g. Theorem II.7.2(iii) in [32]). It now follows easily from the above that sequence of laws $\{{\mathbb {P}}^s_n({{\bar{X}}}^{\scriptscriptstyle {(n)}}_{t_1}-{{\bar{X}}}^{\scriptscriptstyle {(n)}}_{t_0}\in \cdot ):n\in {\mathbb {N}}\}$ on $\mathcal {M}_F({\mathbb {R}}^d)$ are tight.

To show the limit points are unique, assume

6.4 $$\begin{aligned} {{\bar{X}}}^{\scriptscriptstyle (n_k)}_{t_1}-{{\bar{X}}}^{\scriptscriptstyle (n_k)}_{t_0}\overset{w}{\longrightarrow }\tilde{X}_{t_0,t_1}\text { in }\mathcal {M}_F({\mathbb {R}}^d). \end{aligned}$$

It remains to show that

6.5 $$\begin{aligned} {\mathbb {P}}(\tilde{X}_{t_0,t_1}\in \cdot )={\mathbb {N}}_o^s\Bigg (\int _{t_0}^{t_1}X_udu\in \cdot \Bigg ). \end{aligned}$$

(6.4) and the convergence, hence boundedness, in (6.3) with 2*p* in place of *p*, imply that (again one can use Skorokhod’s representation theorem)

$$\begin{aligned} {\mathbb {E}}[{\tilde{X}}_{t_0,t_1}(\phi )^p]=\lim _{k\rightarrow \infty } {\mathbb {E}}^s_{n_k}\left[ \Bigg [\int _{t_0}^{t_1}X_u^{(n_k)}(\phi )du\Bigg ]^p\right] . \end{aligned}$$

This together with (6.3), implies that

6.6 $$\begin{aligned} {\mathbb {E}}[\tilde{X}_{t_0,t_1}(\phi )^p]={\mathbb {N}}_o^s\left[ \Bigg (\int _{t_0}^{t_1}X_u(\phi )du\Bigg )^p\right] \text { for all }\phi \in C_b({\mathbb {R}}^d)\text { and }p\in {\mathbb {N}}. \end{aligned}$$

So to conclude (6.5) we must show the moment problem is well-posed. Assume $\Vert \phi \Vert _\infty \le 1$. Recall that ${\mathbb {P}}_{\delta _o}$ is the probability law of a SBM started from a unit mass at the origin (with $(\gamma , \sigma _0^2)=(1,1)$). Then for $0\le \theta <2/t_1^2$,

$$\begin{aligned}&{\mathbb {E}}_{\delta _0}\left[ \exp \Bigg (\theta \int _{t_0}^{t_1}X_u(\phi )du\Bigg )\right] \\&\quad \le {\mathbb {E}}_{\delta _0}\left[ \exp \Bigg (\theta \int _{0}^{t_1}X_u(1)du\Bigg )\right] \\&\quad \le {\mathbb {E}}_{\delta _0}\left[ \int _0^{t_1}e^{\theta t_1X_u(1)}du/t_1\right] \text { (Jensen applied to the integrand)}\\&\quad \le \exp (t_1\theta [1-(t_1^2\theta /2)]^{-1})<\infty , \end{aligned}$$

where the last line uses the exponential bound in Lemma III.3.6 of [32]. By (1.28), the left-hand side of the above equals

$$\begin{aligned}&\exp \left[ \int \left( \exp \Bigg (\theta \int _{t_0}^{t_1}\nu _u(\phi )du\Bigg )-1\right) \,d{\mathbb {N}}_o(\nu )\right] \\&\quad \ge \exp \left( \int \Bigg ( \exp \Bigl (\theta \int _{t_0}^{t_1}\nu _u(\phi )du\Bigr )-1\Bigg )\mathbb {1}(S>s)\,d{\mathbb {N}}_o(\nu )\right) . \end{aligned}$$

Noting that ${\mathbb {N}}_o(S>s)<\infty$, the above implies that

$$\begin{aligned} \int \exp \Bigg (\theta \int _{t_0}^{t_1}X_u(\phi )du\Bigg )\,d{\mathbb {N}}_o^s<\infty \text { for }0\le \theta <2t_1^{-2}, \end{aligned}$$

which in turn implies that the moment problem for the random variable $\int _{t_0}^{t_1}X_u(\phi )du$ under ${\mathbb {N}}_o^s$ is well-posed (see, e.g. Theorem 3.3.11 in [12]). Therefore (6.6) implies that for non-negative $\phi \in {\mathbb {C}}_b({\mathbb {R}}^d)$ satisfying $\Vert \phi \Vert _\infty \le 1$,

$$\begin{aligned} {\mathbb {P}}({\tilde{X}}_{t_0,t_1}(\phi )\in \cdot )={\mathbb {N}}_o^s\Bigg (\int _{t_0}^{t_1} X_u(\phi )du\in \cdot \Bigg ). \end{aligned}$$

This clearly then follows for all non-negative $\phi \in C_b({\mathbb {R}}^d)$ by linearity. This shows the Laplace functionals of the above two measures are identical and so (6.5) holds (e.g. by Lemma II.5.9 of [32]) and the proof is complete. $\square$

### Proof of Lemma 2.3

Let $t,M,\varDelta$ be as in Condition 7 and set $\ell =\lfloor t\rfloor \in {\mathbb {Z}}_+$, $m=\lfloor \varDelta \rfloor \in {\mathbb {N}}^{\ge 4}$. Assume $x\in {\mathcal {T}}_t$, $\exists x'$ s.t. $(t,x)\overset{\varvec{a}}{\rightarrow }(t+\varDelta ,x')$ and

6.7 $$\begin{aligned} \int _{t+\varDelta }^{t+2\varDelta }|\{y:(t,x)\overset{\varvec{a}}{\rightarrow }(s,y)\}|\,ds\le M. \end{aligned}$$

Clearly $x\in {\mathcal {T}}_\ell$, and $(\ell ,x)\overset{\varvec{a}}{\rightarrow }(t,x)\overset{\varvec{a}}{\rightarrow }(t+\varDelta ,x')$. So by (1.9) and (1.10) $\exists x''\in {\mathcal {T}}_{\ell +m}$ s.t.

6.8 $$\begin{aligned} (\ell ,x)\overset{\varvec{a}}{\rightarrow }(\ell +m,x'')\overset{\varvec{a}}{\rightarrow }(t+\varDelta ,x'). \end{aligned}$$

Next by (6.7) and (1.16),

6.9 $$\begin{aligned} M&\ge \int _{t+\varDelta }^{t+2\varDelta }|\{y:(t,x)\overset{\varvec{a}}{\rightarrow }(s,y)\}|\,ds \nonumber \\&\ge \int _{\ell +m+2}^{\ell +2m}|\{y:(t,x)\overset{\varvec{a}}{\rightarrow }(s,y)\}|\,ds \nonumber \\&=|\{(i,y):(\ell ,x)\overset{\varvec{a}}{\rightarrow }(i,y),\ \ell +m+2\le i\le \ell +2m-1, i\in {\mathbb {N}}\}|. \end{aligned}$$

From (6.8) and (6.9) we see that the left-hand side of (2.6) (in Condition 7) is at most

$$\begin{aligned}&{\mathbb {E}}\Bigg [\sum _{x\in {\mathcal {T}}_\ell }\mathbb {1}\Big (\exists x''\text { s.t. }(\ell ,x)\overset{\varvec{a}}{\rightarrow }(\ell +m,x''),\\&\quad \quad \quad \quad \quad \quad |\{(i,y):(\ell ,x)\overset{\varvec{a}}{\rightarrow }(i,y), \ell +m+2\le i\le \ell +2m-1\}|\le M\Big )\Bigg ]\\&\quad \le c_{7}{\mathbb {P}}\Bigg (S^{\scriptscriptstyle (1)}>m,\, \sum _{i=m+2}^{2m-1}|{\mathcal {T}}_i|\le M\Bigg )\quad (\text {by } 2.7)\\&\quad =c_{7}{\mathbb {P}}\Bigg (S^{\scriptscriptstyle (1)}>\varDelta ,\,\int _{m+2}^{2m}|{\mathcal {T}}_s|ds\le M\Bigg )\\&\quad \le c_{7}{\mathbb {P}}\Bigg (S^{\scriptscriptstyle (1)}>\varDelta ,\int _{\varDelta +2}^{2\varDelta -2}|{\mathcal {T}}_s|ds\le M\Bigg ). \end{aligned}$$

This proves (2.6), as required. $\square$

### Proof of Proposition 2.4

If $\phi _p(x)=|x|^p$, the expression inside the limit on the left side of (2.8) is

6.10 $$\begin{aligned} {\mathbb {E}}\left[ \sum _{x\in {\mathcal {T}}_t}\Bigg (\frac{|x|}{\sqrt{t}}\Bigg )^{p}\right] =m(t){\mathbb {P}}(S>t){\mathbb {E}}_t^1[X_1^{(t)}(\phi _p)]. \end{aligned}$$

By the above, (1.22), and Condition 4 (spatial increments) for $s=t$, we see that for all $p>4$ and $t\ge 1$,

6.11 $$\begin{aligned} {\mathbb {E}}^1_t[X_1^{(t)}(\phi _p)]\le \frac{{\mathbb {E}}\Bigl [\sum _x \mathbb {1}((0,0)\overset{\varvec{a}}{\rightarrow }(t,x))|x|^p\Bigr ]}{t^{p/2}\underline{s}_D}\le \frac{c_4(p)}{\underline{s}_D}. \end{aligned}$$

The assumed weak convergence of the one-dimensional distributions and boundedness in (2.5) for $p=2$ imply weak convergence of the mean measures on ${\mathbb {R}}^d$, i.e.

6.12 $$\begin{aligned} {\mathbb {E}}^1_t[X_1^{(t)}(\cdot )]\overset{w}{\longrightarrow }{\mathbb {N}}_o^1[X_1(\cdot )],\text { as }t\rightarrow \infty . \end{aligned}$$

This weak convergence, the bound in (6.11), and a uniform integrability argument imply that

$$\begin{aligned} \lim _{t\rightarrow \infty }{\mathbb {E}}^1_t[X_1^{(t)}(\phi _p)]={\mathbb {N}}_o^1[X_1(\phi _p)],\quad \forall p\ge 0. \end{aligned}$$

So we may now let $t\rightarrow \infty$ in (6.10) and use the above and Condition 1 (survival probability) to see that the limit in (2.8) is

$$\begin{aligned} s_D\frac{\int X_1(\phi _p)d{\mathbb {N}}_o}{{\mathbb {N}}_o(S>1)}=\frac{\gamma s_D \sigma _0^p}{2}E[|B_1|^p], \end{aligned}$$

where *B* is a standard Brownian motion starting at 0. We have also used (1.29) and the fact that the mean measure of $X_1$ under ${\mathbb {N}}_o$ is the law of $\sigma _0B_1$ (e.g. by Theorem II.7.2 (iii) of [32]). $\square$

### Lemma 6.1

W.p.1 there is a random variable $M\in {\mathbb {N}}$ and $(\mathcal {F}_t)$-stopping times $0=\tau _0<\tau _1<\dots <\tau _M=S^{\scriptscriptstyle (1)}$ such that

$$\begin{aligned} {\mathcal {T}}_t={\left\{ \begin{array}{ll} {\mathcal {T}}_{\tau _{i-1}}&{}\text {on }[\tau _{i-1},\tau _i)\text { for }1\le i\le M\\ \varnothing &{}\text {on }[\tau _M,\infty )=[S^{\scriptscriptstyle (1)},\infty ).\end{array}\right. } \end{aligned}$$

### Proof

Choose $\omega$ so that $S^{\scriptscriptstyle (1)}<\infty$ and (AR)(i)–(iii) hold. In the discrete case the result is clear. Just set $M=S^{\scriptscriptstyle (1)}$ and $\tau _i=i$, and recall (1.13).

Consider next $I=[0,\infty )$. Recall ((1.3), (1.4)) that $({\mathcal {T}}_t,t\ge 0)$ is a cadlag $\mathcal {K}$-valued process taking values in the finite subsets of ${\mathbb {Z}}^d$, $P_F$. It follows that $d_0({\mathcal {T}}_{t-},{\mathcal {T}}_t)\ge 1$ whenever ${\mathcal {T}}_{t-}\ne {\mathcal {T}}_t$ and ${\mathcal {T}}$ is constant between jumps (distinct points in $P_F$ are distance 1 apart). Therefore the jump times cannot accumulate and so can be listed as an increasing sequence of stopping times $0<\tau _1<\tau _2<\dots$, where $\tau _m$ is the *m*th jump time and $\tau _m=\infty$ if there are fewer than *m* jumps. By (1.13) ${\mathcal {T}}_t=\varnothing$ for all $t\ge S^{\scriptscriptstyle (1)}<\infty$ and as $S^{\scriptscriptstyle (1)}$ is a jump time, clearly $S^{\scriptscriptstyle (1)}$ is the last jump time. Moreover the above implies that the number of jumps *M* is an ${\mathbb {N}}$-valued random variable and $\tau _M=S^{\scriptscriptstyle (1)}$. The proof is complete. $\square$

### Proof of Proposition 1.5

Consider first $I={\mathbb {Z}}_+$. Choose $\omega$ s.t. (AR)(i)–(iii) hold. Let $(t,x)\in {\mathcal {\varvec{T}}}$. If $t<1$, then $x=o$, and $w\equiv o$ is the required ancestral path, so assume $j=\lfloor t \rfloor \in {\mathbb {N}}$. We have $(0,o)\overset{\varvec{a}}{\rightarrow }(j,x)$ and so applying (1.10) $j-1$ times we can find $y_0,y_1,\dots ,y_j$ such that $(i-1,y_{i-1})\overset{\varvec{a}}{\rightarrow }(i,y_i)$ for all $1\le i\le j$, $y_0=o$ and $y_j=x$. Now define

$$\begin{aligned} w_s={\left\{ \begin{array}{ll}y_{i-1}&{}\text { if }s\in [i-1,i)\text { for }1\le i\le j\\ x&{}\text { if }s\ge j. \end{array}\right. } \end{aligned}$$

Then clearly *w* is an ancestral path to (*t*, *x*).

If $I=[0,\infty )$ one proceeds as above, choosing $\omega$ so that the conclusion of the previous lemma also holds, and now working with $\{\tau _0,\tau _1,\dots ,\tau _M\}\cap [0,t]$ in place of $\{0,1,\dots ,j\}$. $\square$

## Verifying the conditions for the voter model

Here we verify that (1.3), (AR) and Conditions 1–7 hold for the voter model in dimensions $d\ge 2$ (and hence prove Theorem 4). We first briefly describe the graphical construction of the voter model. This is a standard construction so we refer the reader to Section 2 of [3] for most justifications and further details (or alternatively Example 3.2 of [13]). Let $\{\varLambda (x,y):x,y\in {\mathbb {Z}}^d\}$ be a collection of independent Poisson point processes (ppp’s) on ${\mathbb {R}}_+$ where $\varLambda (x,y)$ has intensity $D(x-y)$. The points in $\varLambda (x,y)([t_1,t_2])$ are the times in $[t_1,t_2]$ when a voter at *y* imposes its opinion at site *x*. At such times an arrow is drawn from *y* to *x*. Let $\mathcal {F}^0_t=\sigma (\{\varLambda (x,y)([0,s]):s\le t, x,y\in {\mathbb {Z}}^d\})$ and $\mathcal {F}_t=\mathcal {F}^0_{t+}$. We assume below that the points in these point processes are all mutually disjoint and strictly positive, thus omitting a set of measure 0.

Recall that the voter model $$(\xi _t)_{t\ge 0}$$ is a $$\{0,1\}^{{\mathbb {Z}}^d}$$-valued Feller process. For each $$t\ge 0$$ and $$x\in {\mathbb {Z}}^d$$ we use the above ppp’s to trace the opinion $$\xi _t(x)$$ back to its source at time 0 by defining a “dual” random walk $$(W^{t,x}_s,0\le s\le t)$$. For this, set $$s_0=0$$, $$W^{t,x}_0=x=y_0$$ and from here we assume $$t>0$$. Let $$t-s_1$$ be the largest time in (0, *t*] when there is an arrow from some $$y_1$$ to $$y_0$$ if such a time exists (so $$s_1=0$$ is possible if there is an arrow at *t*). If no such time exists, set $$s_1=t$$ and $$n=0$$. In general assume we are given $$0<t-s_k<t-s_{k-1}<\cdots <t-s_1\le t$$ and points $$y_0,\dots , y_k$$ so that there is an arrow from $$ y_i$$ to $$y_{i-1}$$ at time $$t-s_i$$ for $$i=1,\dots ,k$$. Let $$t-s_{k+1}\in (0,t-s_k)$$ be the largest time in this interval when there is an arrow from some $$y_{k+1}$$ to $$y_k$$. If no such time exists set $$s_{k+1}=t$$ and $$n=k$$. It is easy to see this process stops after a finite number of steps for all $$t>0$$, $$x\in {\mathbb {Z}}^d$$ a.s. (for fixed (*t*, *x*) it is clear as the arrows are arising with rate 1, and if it is finite for all rational $$t>0$$ and $$x\in {\mathbb {Z}}^d$$, it will be finite for all (*t*, *x*) because for some rational $$q>t$$ there will be no arrows into *x* in (*t*, *q*]). Note that $$n\in {\mathbb {Z}}_+$$ gives the number of steps in the walk and $$s_{n+1}=t$$. Define $$W^{t,x}_s$$ for $$0<s\le t$$ by

$$\begin{aligned} W^{t,x}_s=y_k\text { if }s_k<s\le s_{k+1}\text { for }0\le k\le n. \end{aligned}$$

Then

7.1 $$\begin{aligned}&\{W^{t,x}_s:s\le t\}_{x\in {\mathbb {Z}}^d}\text { is a system of left-continuous, right-limited,} \nonumber \\&\qquad \text {rate one coalescing random walks with step distribution }D. \end{aligned}$$

The above definition easily implies

7.2 $$\begin{aligned}&W^{x,t} \text { is }\mathcal {F}_t-\text { measurable,} \end{aligned}$$

7.3 $$\begin{aligned}&\forall \ 0\le s\le t\ \ W^{t,x}_{t-s}\text { is }\sigma \Bigl (\varLambda (x',y')([s,t']):t'\ge s,\ x',y'\in {\mathbb {Z}}^d\Bigr )-\text {measurable}\\&\qquad \text {and, in particular, is independent of }\mathcal {F}_s, \nonumber \\&W^{t',x'}_{t'-s}=W^{t,x}_{t-s}\text { for some }s\in [0,t\wedge t')\Rightarrow W^{t',x'}_{t'-u}=W^{t,x}_{t-u}\ \ \forall u\in [s,t\wedge t'],\nonumber \end{aligned}$$

and

7.4 $$\begin{aligned} W^{t,x}_{t-u}=W_{s-u}^{s,W_{t-s}^{t,x}}\quad \text {for }0\le u\le s\le t,\ x\in {\mathbb {Z}}^d. \end{aligned}$$

If $$\xi _0\in \{0,1\}^{{\mathbb {Z}}^d}$$, then

7.5 $$\begin{aligned} \xi _t(x)=\xi _0(W^{t,x}_t)\quad \text {for }x\in {\mathbb {Z}}^d,t\ge 0 \end{aligned}$$

defines an $\mathcal {F}_t$-adapted voter model starting at $\xi _0$ with cadlag paths in $\{0,1\}^{{\mathbb {Z}}^d}$ and law $P_{\xi _0}$ on $\mathcal {D}([0,\infty ),\{0,1\}^{{\mathbb {Z}}^d})$. Right-continuity follows from the fact that we include arrows at *t* in our definition of $W^{t,x}$ so for some $\delta >0$, there are no arrows to *x* in $(t,t+\delta ]$ and so $W^{t+\delta ,x}_{t+\delta }=W^{t,x}_t$. Note that (7.4) with $u=0$ and (7.5) imply

7.6 $$\begin{aligned} \xi _t(x)=\xi _s(W^{t,x}_{t-s})\quad \forall \,0\le s\le t,\ x\in {\mathbb {Z}}^d. \end{aligned}$$

If $\xi _0=\mathbb {1}_{\{o\}}$ we write $\xi ^o_t(x)=\mathbb {1}(W^{t,x}_t=o)$ and define

7.7 $$\begin{aligned} {\mathcal {T}}_t=\{x\in {\mathbb {Z}}^d:\xi ^o_t(x)=1\}=\{x:W^{t,x}_t=o\}. \end{aligned}$$

### Lemma 7.1

- If $\xi _0\in \{0,1\}^{{\mathbb {Z}}^d}$ and *A* is a Borel subset of $\{0,1\}^{{\mathbb {Z}}^d}$, then
  7.8 $$\begin{aligned} {\mathbb {P}}(\xi _t\in A|\mathcal {F}_s)=P_{\xi _s}(\xi _{t-s}\in A) \text { a.s. for all }0\le s\le t. \end{aligned}$$
- $t\rightarrow |\mathcal {T}_t|$ is a cadlag $(\mathcal {F}_t)$-martingale s.t. $S^{\scriptscriptstyle (1)}<\infty$ a.s. and in particular $\sup _{t\ge 0}|\mathcal {T}_t|<\infty$ a.s.
- $$(\mathcal {T}_t)_{t\ge 0}$$ satisfies (1.3).

### Proof

(a) Use (7.6) to see that the left-hand side of (7.8) is

$$\begin{aligned} {\mathbb {P}}(\xi _s(W^{t,\cdot }_{t-s})\in A|\mathcal {F}_s)=P_{\xi _s}(\xi _{t-s}\in A)\ \text { a.s.}. \end{aligned}$$

In the above equality we used the fact that

$$\begin{aligned} \varLambda ^s(x,y)([0,u])=\varLambda (x,y)([s,s+u]) \end{aligned}$$

defines a collection of ppp’s equal in law to $\{\varLambda (x,y):x,y\in {\mathbb {Z}}^d\}$ and independent of $\mathcal {F}_s$, which implies that $\{W^{t,x}_{t-s}:x\in {\mathbb {Z}}^d\}$ are equal in law to $\{W^{t-s,x}_{t-s}:x\in {\mathbb {Z}}^d\}$ and are independent of $\mathcal {F}_s$. We also used the fact that $\xi _s$ is $\mathcal {F}_s$-measurable.

(b) See Proposition V.4.1 of [30] and its proof for this, except for the martingale property with respect to the larger filtration $(\mathcal {F}_t)$. This, however, then follows immediately from (a) and Proposition V.4.1(a) of [30].

(c) The fact that $\xi _t$ is cadlag in $\{0,1\}^{{\mathbb {Z}}^d}$ and $|{\mathcal {T}}_t|<\infty$ for all $t\ge 0$ a.s. (by (b)) shows $t\rightarrow {\mathcal {T}}_t$ is cadlag in $\mathcal {K}$. This establishes (1.3). $\square$

Define $(s,y)\overset{\varvec{a}}{\rightarrow }(t,x)$ iff $s\le t$, $x\in {\mathcal {T}}_t$ and $y=W^{t,x}_{t-s}$. It follows that

7.9 $$\begin{aligned} e_{s,t}(y,x)=\mathbb {1}(W^{t,x}_{(t-s)^+}=y,x\in {\mathcal {T}}_t)\text { for all }s,t\ge 0,x,y\in {\mathbb {Z}}^d, \end{aligned}$$

where $(t-s)^+$ is the positive part of $t-s$.

### Lemma 7.2

$\overset{\varvec{a}}{\rightarrow }$ defines an ancestral relation for the voter model.

### Proof

Starting with AR(i), note (1.6) is immediate. Assume $(s,y)\overset{\varvec{a}}{\rightarrow }(t,x)$. By definition $x\in {\mathcal {T}}_t$ and $s\le t$. (7.6) and (7.7) imply that $\xi _s(W^{t,x}_{t-s})=\xi _t(x)=1$ and so $y=W^{t,x}_{t-s}\in {\mathcal {T}}_s$, proving (1.7). (1.8) follows from (7.7).

Turning to (ii), (1.9) is a consequence of (7.4). Assume $(s_1,y_1)\overset{\varvec{a}}{\rightarrow }(s_3,y_3)$. Then $y_1=W^{s_3,y_3}_{s_3-s_1}$ and if $y_2=W^{s_3,y_3}_{s_3-s_2}$, then $(s_2,y_2)\overset{\varvec{a}}{\rightarrow }(s_3,y_3)$ by definition. By (7.4) we have $W^{s_2,y_2}_{s_2-s_1}=W^{s_3,y_3}_{s_3-s_1}=y_1$ and so $(s_1,y_1)\overset{\varvec{a}}{\rightarrow }(s_2,y_2)$. This gives (ii).

We will use Remark 1.1(2) to verify (iii). The fact that $s\rightarrow e_s(t,x)$ is cadlag on $[0,\infty )$ is immediate from (7.9) and the fact that $s\rightarrow W_s^{t,x}\in {\mathbb {Z}}^d$ is left-continuous with right limits on [0, *t*] (by (7.1)). There is a $\delta >0$ such that there is no arrow towards *x* in $(t,t+\delta ]$. Let $r\in (t,t+\delta ]$. Then by definition

7.10 $$\begin{aligned} W^{r,x}_s=x\quad \forall s\in [0,r-t], \end{aligned}$$

which by (7.6) implies

$$\begin{aligned} \xi _r(x)=\xi _t(W^{r,x}_{r-t})=\xi _t(x), \end{aligned}$$

and therefore

7.11 $$\begin{aligned} x\in {\mathcal {T}}_t\ \text { iff }\ x\in {\mathcal {T}}_r. \end{aligned}$$

Next, use (7.4) with (*u*, *s*, *t*) replaced by (*s*, *t*, *r*) to see that,

7.12 $$\begin{aligned} W_{r-s}^{r,x}=W_{t-s}^{t,W^{r,x}_{r-t}}=W_{t-s}^{t,x}\ \text { for all }s\le t, \end{aligned}$$

where (7.10) is used in the last equality. We also have

7.13 $$\begin{aligned} W^{r,x}_{(r-s)^+}=x=W^{t,x}_{(t-s)^+}\quad \text {for all }s\ge t, \end{aligned}$$

where we use (7.10) for the first equality when $r\ge s\ge t$. Now use (7.11), (7.12) and (7.13) in (7.9) to conclude that ${{\hat{e}}}_t(y,x)={{\hat{e}}}_r(y,x)$ for all $r\in [t,t+\delta ]$. This proves the first condition in Remark 1.1(2). For the second condition, (1.15), if $x\in {\mathcal {T}}_{t-}$ choose $\delta >0$ such that there are no arrows to *x* in $[t-\delta ,t)$ and proceed in a similar manner. This completes the proof of AR(iii).

If $s< t$, (7.9) shows that $e_{s,t}(y,x)$ is $\mathcal {F}_t$-measurable by (7.2) and the $\mathcal {F}_t$-adaptedness of ${\varvec{{\mathcal {T}}}}$. This gives (AR)(iv) and the proof is complete. $\square$

For $t\ge 0$ and $x\in {\mathbb {Z}}^d$ we define our candidate for an ancestral path to $(t,x)\in {\mathcal {\varvec{T}}}$ by

7.14 $$\begin{aligned} w_s(t,x)= W^{t,x}_{(t-s)^+}. \end{aligned}$$

### Lemma 7.3

For any $(t,x)\in {\mathcal {\varvec{T}}}$, *w*(*t*, *x*) is the unique ancestral path to (*t*, *x*), and therefore $\mathcal {W}=\{w(t,x):(t,x)\in {\mathcal {\varvec{T}}}\}$.

### Proof

Assume that $(t,x)\in {\mathcal {\varvec{T}}}$. Then $s\mapsto w_s(t,x)$ is cadlag by definition and the fact that $W_s^{t,x}$ is left-continuous with right limits in *s*. (7.6) implies that if $0\le s\le t$, then $1=\xi _t(x)=\xi _s(w_s(t,x))$ and so

7.15 $$\begin{aligned} w_s(t,x)\in {\mathcal {T}}_s\text { for all }s\le t. \end{aligned}$$

Let $0\le s\le s'\le t$. Then, using (7.15), we see that $(s,W^{t,x}_{t-s})\overset{\varvec{a}}{\rightarrow }(s',W^{t,x}_{t-s'})$ iff $W_{t-s}^{t,x}=W_{s'-s}^{s', W^{t,x}_{t-s'}}$, which holds by (7.4). As $w_s(t,x)=W^{t,x}_0=x$ for all $s\ge t$, we see that *w*(*t*, *x*) is an ancestral path to (*t*, *x*).

Turning to uniqueness, let ${\tilde{w}}_s(t,x)$ be any ancestral path to (*t*, *x*). Then $(0,o)\overset{\varvec{a}}{\rightarrow }(s,{\tilde{w}}_s(t,x)$) implies $\tilde{w}_s(t,x)=W^{t,x}_{t-s}$, and so ${\tilde{w}}(t,x)=w(t,x)$ is unique. The last assertion is then immediate. $\square$

Before proving Theorem 4 we note that the above definition of *w*(*t*, *x*) and part (v1) of the Theorem give a uniform modulus of continuity for the rescaled dual coalescing random walks connecting one-valued sites in the voter model conditioned on longterm survival.

### Corollary 7.4

Assume $\{W^{t,x}_s:0\le s\le t, x\in {\mathbb {Z}}^d\}$ ($d\ge 2$) is the coalescing dual of a voter model $({\mathcal {T}}_t)_{t\ge 0}$ starting with a single one at the origin, with bounded range kernel *D* and survival time $S^{\scriptscriptstyle (1)}$. Let $\alpha \in (0,1/2)$. There is a constant $C_{7.4}$ and for all $n\ge 1$ a random variable $\delta _n\in [0,1]$ so that

7.16 $$\begin{aligned} {\mathbb {P}}(\delta _n\le \rho |S^{\scriptscriptstyle (1)}>nt^*)\le C_{7.4}(t^*\vee 1)[\rho +n^{-1}],\ \forall \rho \in [0,1),\ t^*>0, \end{aligned}$$

and if $(t,x)\in {\mathcal {\varvec{T}}}$, $0\le s_1<s_2\le t$, and $|s_2-s_1|\le n\delta _n$,

7.17 $$\begin{aligned} |W^{t, x}_{s_1}-W^{t, x}_{s_2}|\le C_{7.4}n^{(1/2)-\alpha }[|s_2-s_1|^\alpha +1]. \end{aligned}$$

### Proof

Let $\delta _n$ be as in Theorem 4(v1) (see Definition 2.5). Then for $n\ge 1$, $t^*>0$, and $\rho \in [0,1)$, that Theorem gives

$$\begin{aligned} {\mathbb {P}}(\delta _n\le \rho |S^{\scriptscriptstyle (1)}>nt^*)&\le m(n) {\mathbb {P}}(\delta _n\le \rho )/(m(n){\mathbb {P}}(S^{\scriptscriptstyle (1)}>nt^*))\\&\le C_{1}[\rho +n^{-1}]{\underline{s}}_D^{-1}c_{1.21}(t^*\vee 1), \end{aligned}$$

where (1.31) is used in the last line. This gives (7.16), and (7.17) is then immediate from Corollary 1, (7.14) and Lemma 7.3. $\square$

### Proof of Theorem 4

Parts (v1), (v2) and (v3) will follow from Theorems 1, 2 and 3, respectively, once we verify Conditions 1–7 for the parameter values given in Theorem 4. Here we need to recall that $s_D=\beta _D^{-1}$ for the voter model (Proposition 1.6), and carry out a bit of arithmetic (especially for (v3)). We have already noted that Conditions 1 and 6 follow from Propositions 1.6(b) and 1.7, respectively. Condition 2 follows immediately from Lemma 7.1(b). So it remains to check Conditions 3, 4, 5 and 7 for the voter model. $\square$

### Condition 3

On $\{y\in {\mathcal {T}}_s\}$ we have

$$\begin{aligned} {\mathbb {P}}(\exists z\text { s.t. }(s,y)\overset{\varvec{a}}{\rightarrow }(s+t,z)|\mathcal {F}_s)&={\mathbb {P}}(\exists z\text { s.t. }W^{s+t,z}_t=y|\mathcal {F}_s)\\&={\mathbb {P}}(\exists z\text { s.t. }W_t^{s+t,z}=y)\quad (\text {by } (7.3))\\&={\mathbb {P}}(\exists z\text { s.t. }W_s^{s,z}=0), \end{aligned}$$

where in the last line we used translation invariance in both space and time of the system of Poisson point processes $\{\varLambda (x,y)\}$. More specifically we use the fact that $\{\varLambda (x'-y,y'-y)([t,t+u]):x',y'\in {\mathbb {Z}}^d,u\ge 0\}$ has the same law as $\{\varLambda (x',y')([0,u]):x',y'\in {\mathbb {Z}}^d,u\ge 0\}$. Recalling (7.7) we see that the right-hand side of the above equals

$$\begin{aligned} {\mathbb {P}}({\mathcal {T}}_s\text { is non-empty})\le \frac{{\overline{s}}_D}{m(s)}, \end{aligned}$$

by (1.22) (which applies because Condition 1 holds).

### Condition 7

On $\{x\in {\mathcal {T}}_t\}$ we can argue as above to see that

$$\begin{aligned}&{\mathbb {P}}\Bigg (\exists x'\text { s.t. }(t,x)\overset{\varvec{a}}{\rightarrow }(t+\varDelta ,x'),\ \int _{t+\varDelta }^{t+2\varDelta }|\{y:(t,x)\overset{\varvec{a}}{\rightarrow }(s,y)\}|ds\le M\Bigr |\mathcal {F}_t\Bigg )\\&\quad ={\mathbb {P}}\Bigg (\exists x'\text { s.t. }W_\varDelta ^{t+\varDelta ,x'}=x,\ \int _{t+\varDelta }^{t+2\varDelta }|\{y:W^{s,y}_{s-t}=x\}|ds\le M\Bigr |\mathcal {F}_t\Bigg )\\&\quad ={\mathbb {P}}\Bigg (\exists x'\text { s.t. }W_\varDelta ^{t+\varDelta ,x'}=x,\ \int _{t+\varDelta }^{t+2\varDelta }|\{y:W^{s,y}_{s-t}=x\}|ds\le M\Bigg )\ (\text {by }(7.3))\\&\quad ={\mathbb {P}}\Bigg (\exists x'\text { s.t. }W_\varDelta ^{t+\varDelta ,x'}=x,\ \int _{\varDelta }^{2\varDelta }|\{y:W^{s'+t,y}_{s'}=x\}|ds'\le M\Bigg )\ (s'=s-t)\\&\quad ={\mathbb {P}}\Bigl (\exists x'\text { s.t. }W_\varDelta ^{\varDelta ,x'}=o,\ \int _\varDelta ^{2\varDelta }|\{y:W^{s,y}_{s}=o\}|ds\le M\Bigr ), \end{aligned}$$

where in the last line we use the the translation invariance in space and time of the system of ppp’s as above. Now use (7.7) to see that the above equals

$$\begin{aligned} {\mathbb {P}}\Bigg (S^{\scriptscriptstyle (1)}>\varDelta ,\ \int _\varDelta ^{2\varDelta }|{\mathcal {T}}_s|\,ds\le M\Bigg ). \end{aligned}$$

Using this equality, the left-hand side of (2.6) (in Condition 7) is equal to

$$\begin{aligned}&{\mathbb {E}}\left[ \sum _{x\in {\mathcal {T}}_t}{\mathbb {P}}\Bigg (S^{\scriptscriptstyle (1)}>\varDelta ,\ \int _\varDelta ^{2\varDelta }|{\mathcal {T}}_s|\,ds\le M\Bigg )\right] \\&\quad ={\mathbb {E}}[|{\mathcal {T}}_t|]{\mathbb {P}}\Bigl (S^{\scriptscriptstyle (1)}>\varDelta ,\ \int _\varDelta ^{2\varDelta }|{\mathcal {T}}_s|\,ds\le M\Bigr ). \end{aligned}$$

Recall that ${\mathbb {E}}[|{\mathcal {T}}_t|]=1$ by Lemma 7.1(b), and so if $\varDelta \ge 4$ the above is trivially bounded above by the right-hand side of (2.6) with $c_{7}=1$, and so Condition 7 is established.

### Condition 4

Recall that for $0\le s\le t$, $(t-s,y)\overset{\varvec{a}}{\rightarrow }(t,x)$ iff $x\in {\mathcal {T}}_t$ and $y=W^{t,x}_{s}$. Therefore by translation invariance of $W^{t,x}_s$, we have for any $p>4$,

7.18 $$\begin{aligned} {\mathbb {E}}\Bigg [&\sum _{x\in {\mathcal {T}}_t}\sum _{y\in {\mathcal {T}}_{t-s}}\mathbb {1}((t-s,y)\overset{\varvec{a}}{\rightarrow }(t,x))|x-y|^p\Bigg ]\nonumber \\&={\mathbb {E}}\Bigg [\sum _{x\in {\mathcal {T}}_t}\sum _{y\in {\mathbb {Z}}^d}\mathbb {1}(W_s^{t,x}=y)|x-W^{t,x}_s|^p\Bigg ] \nonumber \\&={\mathbb {E}}\Bigg [\sum _{x\in {\mathbb {Z}}^d}\mathbb {1}(W^{t,x}_t=o)|x-W^{t,x}_s|^p\Bigg ]\ (\text {by } (7.7)) \nonumber \\&={\mathbb {E}}\Bigg [\sum _{x\in {\mathbb {Z}}^d}\mathbb {1}(W^{t,o}_t=-x)|W^{t,o}_s|^p\Bigg ] \nonumber \\&={\mathbb {E}}[|W^{t,o}_s|^p]. \end{aligned}$$

Recall (see (7.1)) $s\mapsto W^{t,o}_s$ is a rate one continuous time rw with step distribution *D* and so has steps bounded in Euclidean norm by *L*. If $S_n=\sum _{i=1}^n Z_i$ denotes the corresponding discrete time rw and $N_s$ is an independent rate one Poisson process, then we can use Burkholder’s predictable square function inequality (Theorem 21.1 in [5]) to see that

$$\begin{aligned} {\mathbb {E}}[|W^{t,o}_s|^p]&={\mathbb {E}}[|S_{N_s}|^p]\\&\le c_p{\mathbb {E}}[N_s^{p/2}+\max _{i\le N_s}|Z_i|^p]\\&\le c_p{\mathbb {E}}\left[ N_s^{p/2}+N_s\Bigg [\sum _x |x|^pD(x)\Bigg ]\right] \\&\le c(p,L)(s\vee 1)^{p/2}, \end{aligned}$$

and we arrive at Condition 4 for any $p>4$.

### Condition 5

To verify Condition 5 for all $\kappa >4$ we will dominate the range of the voter model by a pure birth process. The following result is standard (e.g. see Theorem 11 in Sec. 6.11 of [16] and use a stopping time argument to add values of $s=e^\lambda >1$ to those considered there).

### Lemma 7.5

Let $M_t$ denote a rate one pure birth process with $M_0=1$. Then for all $t,\lambda \ge 0$ satisfying $\lambda <-\ln (1-e^{-t})$,

$$\begin{aligned} {\mathbb {E}}[e^{\lambda M_t}]=(1+e^{t-\lambda }-e^t)^{-1}, \end{aligned}$$

and so if $\lambda _{7.5}=-\ln (1-e^{-2})/2$, there is a $C_{7.5}$ such that

7.19 $$\begin{aligned} {\mathbb {P}}(M_2\ge N)\le C_{7.5} e^{-\lambda _{7.5}N}\quad \forall N>0. \end{aligned}$$

To verify Condition 5 we couple the voter model $\xi _t(x)=\mathbb {1}(x\in {\mathcal {T}}_t)$ with a rate one branching random walk $Z_t(x)\in {\mathbb {Z}}_+,\ t\ge 0, x\in {\mathbb {Z}}^d$, so that $Z_0(x)=\xi _0(x)=\mathbb {1}(x=o)$ and $\xi _t(x)\le Z_t(x)$ for all *t*, *x*. This is standard so we only sketch the construction.

We extend the system of Poisson point processes used to construct the dual coalescing rw’s $\{W^{t,x}_s\}$ by considering an i.i.d. system of such processes $\varLambda _i,i\ge 1$, where $\varLambda _1=\varLambda$. Then every time $\varLambda _i(x,y)$ jumps at time *t*, and $Z_t(y)\ge i$, particle *i* at *y* will produce an offspring at *x*. In this way one can easily check that $Z_t$ is a rate one branching random walk with offspring law *D*. Moreover since $M_t=\sum _xZ_t(x)$ is a rate one pure birth process, and so is finite for all times, we can order the jumps of *Z* and $\xi$ as $0<T_1<T_2<\cdots$ (recall that the range of the voter model is finite a.s.). It is then easy to induct on *n* to check that $\xi _{T_n}\le Z_{T_n}$ (coordinatewise). (Here one really only needs check times at which a new one appears in $\xi _{T_n}$ at location *x*.) Since $Z_t$ is monotone increasing, this implies that

7.20 $$\begin{aligned} |R^{\scriptscriptstyle (1)}_t|\le M_t\quad \text {for all }t\ge 0, \end{aligned}$$

where we recall that $R^{\scriptscriptstyle (1)}_t$ is the range of the voter model up to time *t*.

Recall that $w_s(t,x)=W^{t,x}_{(t-s)^+}$ is the unique ancestral path to $(t,x)\in {\mathcal {\varvec{T}}}$. The independence in (7.3) and translation invariance of the system of Poisson point processes in the graphical construction of $\xi$, imply that for $s\ge 0, y\in {\mathbb {Z}}^d$ fixed, and on $\{y\in {\mathcal {T}}_s\}$,

$$\begin{aligned} {\mathbb {P}}&(\exists \ (t,x)\text { s.t. } (s,y)\overset{\varvec{a}}{\rightarrow }(t,x),\ t\in [s,s+2],\ |y-x|\ge N|\mathcal {F}_s)\\&={\mathbb {P}}(\exists \ (t,x)\text { s.t. } (0,o)\overset{\varvec{a}}{\rightarrow }(t,x),\ t\in [0,2],\ |x|\ge N)\\&\le {\mathbb {P}}(|\{w_s(t,x):s\in [0,t]\}|\ge N/(\sqrt{d} L)\text { for some }t\in [0,2]\text { and }x\in {\mathcal {T}}_t)\\&\le {\mathbb {P}}(|R^{\scriptscriptstyle (1)}_2|\ge N/(\sqrt{d} L)). \end{aligned}$$

The first inequality holds since $s\rightarrow w_s(t,x)=W^{t,x}_{t-s}$ is a step function from *o* to *x* taking steps of (Euclidean) length at most $\sqrt{d} L$, and the second holds since for $x\in {\mathcal {T}}_t$, the range of $w_\cdot (t,x)$ is in $R^{\scriptscriptstyle (1)}_t\subset R^{\scriptscriptstyle (1)}_2$ for $t\le 2$.

Now use (7.19) and (7.20) to see the above upper bound is at most

$$\begin{aligned} {\mathbb {P}}(M_2\ge N/(\sqrt{d} L))\le C_{7.5}\exp {\Bigl (-\frac{\lambda _{7.5}}{\sqrt{d} L}N}\Bigr ). \end{aligned}$$

This implies Condition 5 for each $\kappa >4$, and so completes the proof of Theorem 4. $\square$

## Verifying the conditions for oriented percolation

Recall the discussion after Proposition 1.5, and that in particular $\overset{\varvec{a}}{\rightarrow }$ defined therein for oriented percolation is an ancestral relation.

Here we verify that Conditions 1-7 hold for sufficiently spread out critical oriented percolation in dimensions $d>4$ (and hence prove Theorem 5).

### Condition 1

This was verified in Proposition 1.6 with $m(t)=A^2V(t \vee 1)$ and $s_D=2A$. $\square$

### Condition 2

This is immediate from [44, Theorem 1.11(a)] with $k=0$. $\square$

### Condition 3

This is a trivial consequence of Condition 1, since the event that there is an occupied path from (*s*, *y*) to $(s+t,z)$ (for some *z*) is independent of $\mathcal {F}_s$, and has probability $\theta (t)={\mathbb {P}}({\mathcal {T}}_t \ne \varnothing )$, by the translation invariance of the model.

We will show below that Condition 4 is a consequence of the following two lemmas, the first of which is Theorem 1.1 of [33] (it also is a special case of Theorem 1.2 of [6] with $\alpha =\infty$; see the comment in [6] after Theorem 1.2).

### Lemma 8.1

For $d>4$ if *L* is sufficiently large, then for all $p>4$ there exists $C_L(p)$ such that for all $n\in {\mathbb {Z}}_+$,

$$\begin{aligned} \sum _x |x|^p {\mathbb {P}}(x \in \mathcal {T}_n)\le C_L(p) n^{p/2}. \end{aligned}$$

### Lemma 8.2

Let $f:{\mathbb {Z}}^d \rightarrow {\mathbb {R}}_+$. Then for oriented percolation (critical, spread out, in dimensions $d>4$), and $m<n\in {\mathbb {Z}}_+$

8.1 $$\begin{aligned} {\mathbb {E}}\left[ \sum _{x\in {\mathcal {T}}_n}\sum _{y\in {\mathcal {T}}_{m}}\mathbb {1}((m,y)\rightarrow (n,x))f(x-y)\right]&={\mathbb {E}}[\mathcal {T}_{m}] \sum _{z\in {\mathbb {Z}}^d}f(z){\mathbb {P}}(z \in \mathcal {T}_{n-m})\nonumber \\&\le c_2 \sum _{z\in {\mathbb {Z}}^d}f(z){\mathbb {P}}(z \in \mathcal {T}_{n-m}) \end{aligned}$$

### Proof

Let $\mathcal {C}(n,(m,z))=\{x:(m,z)\rightarrow (n+m,x)\}$. Then the left hand side is equal to

$$\begin{aligned}&\sum _{x,y\in {\mathbb {Z}}^d}f(x-y){\mathbb {P}}(y \in {\mathcal {T}}_m,x \in \mathcal {C}(n-m,(m,y))) \\&\quad =\sum _{x,y\in {\mathbb {Z}}^d}f(x-y){\mathbb {P}}\big (y\in {\mathcal {T}}_m){\mathbb {P}}\big (x \in \mathcal {C}(n-m,(m,y))\big )\\&\quad =\sum _{x,y\in {\mathbb {Z}}^d}f(x-y){\mathbb {P}}\big (y\in {\mathcal {T}}_m){\mathbb {P}}\big (x-y \in \mathcal {C}(n-m,(0,o))\big )\\&\quad =\sum _y {\mathbb {P}}\big (y\in {\mathcal {T}}_m\big ) \sum _z f(z) {\mathbb {P}}\big (z \in \mathcal {C}(n-m,(0,o))\big )\\&\quad ={\mathbb {E}}[\mathcal {T}_{m}]\sum _z f(z) {\mathbb {P}}\big (z \in \mathcal {T}_{n-m}\big ), \end{aligned}$$

giving the equality. The inequality is then immediate from Condition 2. $\square$

### Condition 4

Assume *L* is sufficiently large so that the above two lemmas hold. Let $p>4$ and $f(x)=|x|^p$, $n=\lfloor t\rfloor$, and $m=\lfloor t-s\rfloor$. Then the left hand sides of (2.1) and (8.1) are identical. Therefore Lemma 8.2 shows that the left hand side of (2.1) is at most

$$\begin{aligned} c_2 \sum _{z\in {\mathbb {Z}}^d}|z|^p{\mathbb {P}}(z \in \mathcal {T}_{n-m}). \end{aligned}$$

By Lemma 8.1 this is at most $C_L (n-m)^{p/2}\le C'_L(s\vee 1)^{p/2}$ and so Condition 4 is verified for any $p>4$. $\square$

### Condition 5

This is immediate for any $\kappa >4$ when our steps are within a box of size *L* (see Remark 2.1). Note that, more generally, the left hand side of (2.2) is (by independence and translation invariance),

$$\begin{aligned} {\mathbb {P}}(\exists (t,x)\text { s.t. }t\in [0,2], x \in {\mathcal {T}}_{t},|x|\ge N)&\le \sum _{|x|\ge N}{\mathbb {P}}(x \in {\mathcal {T}}_1)+\sum _{|x|\ge N}{\mathbb {P}}(x' \in {\mathcal {T}}_2)\\&\le p_c \sum _{|x|\ge N} D(x)+ p_c^2 \sum _{|x|\ge N} D^{(*2)}(x)\\&\le C \sum _{|x|\ge N/2}D(x)\\&\le C \frac{\sum _x |x|^{4+\varepsilon }D(x)}{N^{4+\varepsilon }}, \end{aligned}$$

which satisfies the required bound with $\kappa =4+\varepsilon$ provided that *D* has $4+\varepsilon$ finite moments for some $\varepsilon >0$. $\square$

### Condition 6

We verify the conditions of Lemma 2.2 with $(\gamma , \sigma _0^2)=(1, \sigma ^2_{\scriptscriptstyle D}v)$. The first condition holds by [44, Theorem 1.2] together with the survival asymptotics [38, Theorem 1.5] and [24, Proposition 2.4]. The second condition holds since (by Condition 1)

$$\begin{aligned} {\mathbb {E}}_n^s[X_t^{\scriptscriptstyle (n)}(1)^p]\le C_s\frac{n}{n^p}\sum _{x_1,\dots , x_p}{\mathbb {P}}\left( \cap _{i=1}^p\{x _i \in {\mathcal {T}}_{\lfloor nt \rfloor }\}\right) , \end{aligned}$$

and by [44, Theorem 1.2] (with $\mathbf {k}=\mathbf {0}$) the sum is at most $C_{s,t^*,p}n^{p-1}$ for all $t\le t^*$.

$$\square $$

### Condition 7

We use Lemma 2.3. By independence of bond occupation status before time $\ell$ and after time $\ell$, and translation invariance, the left hand side of (2.7) is equal to

$$\begin{aligned}&\sum _x {\mathbb {P}}\big (x \in {\mathcal {T}}_\ell , \exists x'\in {\mathbb {Z}}^d\text { s.t. }(\ell ,x)\rightarrow (\ell +m,x'),\\&\quad \quad \quad \quad |\{(i,y):(\ell ,x)\rightarrow (i,y), m+2\le i-\ell \le 2m-1\}|\le M\big )\\&\quad =\sum _x {\mathbb {P}}\big (x \in {\mathcal {T}}_\ell \big )\\&\quad \quad \quad \quad \times {\mathbb {P}}(\exists z\in {\mathcal {T}}_m,|\{(i,y):(0,o)\rightarrow (i,y), m+2\le i\le 2m-1\}|\le M\big )\\&\quad =\left[ \sum _x {\mathbb {P}}\big (x \in {\mathcal {T}}_\ell \big )\right] {\mathbb {P}}\left( S^{(1)}>m,\ \sum _{i=m+2}^{2m-1}|{\mathcal {T}}_i|\le M\right) . \end{aligned}$$

From Condition 2 we see that the first term is bounded by a constant as required. $\square$

### Proof of Theorem 5

Conditions 1–7 all hold, with $s_D=2A$ in Condition 1, any $p>4$ in Condition 4, any $\kappa >4$ in Condition 5 and $(\gamma , \sigma _0^2)=(1, \sigma ^2_{\scriptscriptstyle D}v)$ in Condition 6. Hence, with a bit of elementary arithmetic, Theorem $1^\prime$ implies (op1), Theorem 2 implies (op2), and Theorem 3 implies (op3). $\square$

We end with a more explicit interpretation of the modulus of continuity in Theorem 5.

### Corollary 8.3

Let $\varvec{{\mathcal {T}}}$ be critical oriented percolation with $d>4$, survival time $S^{\scriptscriptstyle (1)}$, and *L* sufficiently large so that the hypotheses of Theorem 5 hold. Assume $\alpha \in (0,1/2)$. Then there is a constant $C_{8.3}$, and for any $n\ge 1$ a random variable $\delta _n\in (0,1]$ so that

8.2 $$\begin{aligned} {\mathbb {P}}(\delta _n\le \rho |S^{\scriptscriptstyle (1)}>nt^*)\le C_{8.3}(t^*\vee 1)\rho \ \ \forall \rho \in [0,1),\ t^*>0, \end{aligned}$$

and if $(0,o)\rightarrow (k_1,x_1)\rightarrow (k_2,x_2)$, $k_1,k_2\in {\mathbb {Z}}_+$, and $|k_2-k_1|\le n\delta _n$, then

$$\begin{aligned} |x_2-x_1|\le C_{8.3}|k_2-k_1|^\alpha n^{(1/2)-\alpha }. \end{aligned}$$

### Proof

This follows immediately from Corollary $1^\prime$, and a short calculation to derive (8.2). The latter is similar to the derivation of (7.16) in the proof of Corollary 7.4. $\square$

## Verifying the conditions for lattice trees

Recall that in Sect. 1 (following Proposition 1.5) we saw that (1.3) and (AR), except for (AR)(iv), were elementary. Here we verify that (AR)(iv) and Conditions 1–7 hold for sufficiently spread out critical lattice trees in dimensions $d>8$, and hence prove Theorem 6.

Recall that we defined $\overset{\varvec{a}}{\rightarrow }$ by

$$\begin{aligned} (k,y)\overset{\varvec{a}}{\rightarrow }(m,x) \quad \iff \quad x\in {\mathcal {T}}_m, \, 0\le k\le m, \text { and }w_k(m,x)=y, \end{aligned}$$

where $w(m,x)=(w_k(m,x))_{k\le m}$ is the unique path in the tree $\varvec{{\mathcal {T}}}$ from *o* to *x*. Given $T\in {\mathbb {T}}_L(o)=:{\mathbb {T}}$, we let $T_{\le n}$ denote the subtree consisting of vertices in $\cup _{m\le n}T_m$ and all the bonds in *E*(*T*) between these vertices. Clearly $T_{\le n}$ is connected because for any $n'\le n$ and $x\in T_{n'}$, $(w_m(n',x))_{m\le n'}$ is a path in $T_{\le n}$ from *o* to *x*. It follows that $T_{\le n}$ is a tree and clearly the set ${\mathbb {T}}_{\le n}=\{T_{\le n}:T\in {\mathbb {T}}\}\subset {\mathbb {T}}$ is a finite set of trees. It also follows that for any $x\in {\mathbb {Z}}^d$

9.1 $$\begin{aligned} \mathbb {1}(x\in T_n)\mathbb {1}(w(n,x)\in A)\text { is a function of }T_{\le n} \text { for any} A\subset {\mathbb {T}}_{\le n}. \end{aligned}$$

Choosing a random tree $\varvec{{\mathcal {T}}}$ according to ${\mathbb {P}}$, we see that ${\mathcal {T}}_{\le n}$ is a random tree. We define

9.2 $$\begin{aligned} \mathcal {F}_n=\{\{\mathcal {T}_{\le n}\in A\}: A\text { is a subset of }{\mathbb {T}}_{\le n}\},\ n\in {\mathbb {Z}}_+, \end{aligned}$$

that is, $\mathcal {F}_n$ is just the $\sigma$-field generated by ${\mathcal {T}}_{\le n}$. Since ${\mathcal {T}}_{\le n}$ is a function of ${\mathcal {T}}_{\le n+1}$, $(\mathcal {F}_n)_{n\in {\mathbb {Z}}_+}$ is a filtration and clearly

$$\begin{aligned} {\mathcal {T}}_n=V({\mathcal {T}}_{\le n}){\setminus } V({\mathcal {T}}_{\le n-1})\text { is }\mathcal {F}_n\text {-measurable}. \end{aligned}$$

We can now verify (AR)(iv). Let $m,n\in {\mathbb {Z}}_+$ and $x,y\in {\mathbb {Z}}^d$. If $m< n$ then $e_{m,n}(y,x)=\mathbb {1}((m,y)\overset{\varvec{a}}{\rightarrow }(n,x))=\mathbb {1}(x \in \mathcal {T}_n,w_m(n,x)=y)$, which is $\mathcal {F}_n$-measurable by (9.1). If $m\ge n$ then $e_{m,n}(y,x)=\mathbb {1}(x=y \in {\mathcal {T}}_n)$, which is also $\mathcal {F}_n$-measurable by(9.2). This verifies (AR)(iv) as required.

For $x \in {\mathcal {T}}_n$ define the extended path $w'(n,x)$ by

$$\begin{aligned} w'_m(n,x)=w_m(n,x)\mathbb {1}(m<n)+x\mathbb {1}(m\ge n). \end{aligned}$$

It is then immediate from the definition of *w*(*n*, *x*) that $w_{m_1}(n,x)\in {\mathcal {T}}_{m_1}$ for $m_1\le n$ and that $w_{m_0}(m_1,w_{m_1}(n,x))=w_{m_0}(n,x)$ for $m_0\le m_1\le n$. Thus $w'(n,x)$ is an ancestral path to $(n,x)\in \mathbf {\mathcal {T}}$. Moreover it is easy to see $w'(n,x)$ is the only ancestral path to (*n*, *x*) and hence

9.3 $$\begin{aligned} \mathcal {W}:=\{w'(n,x):(n,x)\in \mathbf {\mathcal {T}}\} \end{aligned}$$

is the system of ancestral paths for $(\varvec{{\mathcal {T}}},\overset{\varvec{a}}{\rightarrow })$. Before verifying Conditions 1–7 with parameters as in Theorem 6, and hence verifying the conclusion of Corollary $1^\prime$, we can use the above characterization of $\mathcal {W}$ in (9.3) to give an explicit interpretation of this corollary. It is a large scale modulus of continuity for $w_k(m,x), k\le m$ conditional on longterm survival of the tree.

### Corollary 9.1

Let $\varvec{{\mathcal {T}}}$ be the critical lattice tree with $d>8$, *L* sufficiently large so that the hypotheses of Theorem 6 hold, and survival time $S^{\scriptscriptstyle (1)}$. Assume $\alpha \in (0,1/2)$. Then there is a constant $C_{9.1}$, and for any $n\ge 1$ a random variable $\delta _n\in (0,1]$ so that

9.4 $$\begin{aligned} {\mathbb {P}}(\delta _n\le \rho |S^{\scriptscriptstyle (1)}>nt^*)\le C_{9.1}(t^*\vee 1)\rho \ \ \forall \rho \in [0,1),\ t^*>0, \end{aligned}$$

and if $(m,x)\in {\mathcal {\varvec{T}}}$, $k_1,k_2\in {\mathbb {Z}}_+$, $k_i\le m$, and $|k_2-k_1|\le n\delta _n$, then

$$\begin{aligned} |w_{k_2}(m,x)-w_{k_1}(m,x)|\le C_{9.1}|k_2-k_1|^\alpha n^{(1/2)-\alpha }. \end{aligned}$$

### Proof

This follows immediately from Corollary $1^\prime$, (9.3), and a short calculation to derive (9.4), as in Corollary 8.3. $\square$

In the remainder of this section we verify Conditions 1–7 for critical sufficiently spread-out lattice trees in dimensions $d>8$. Condition 4 will be verified below as a consequence of the following bound on the moments of the two-point function from [33] (Theorem 1.3). (It was first proved in [25], for $p=6$.)

### Lemma 9.2

([33]) For $d>8$, *L* sufficiently large and $p>4$ there exists $C=C(d,L,p)>0$ such that for all $n\in {\mathbb {Z}}_+$,

$$\begin{aligned} \sum _x |x|^p {\mathbb {P}}(x \in \mathcal {T}_n)\le Cn^{p/2}. \end{aligned}$$

### Condition 1

This was checked in Proposition 1.6 with $m(t)=A^2V(t \vee 1)$ and $s_D=2A$. $\square$

### Condition 2

This is immediate from [22, Theorem 1.12] with $k=0$. $\square$

### Condition 5

For lattice trees (2.3) holds and hence so does Condition 5 for any $\kappa >4$ (Remark 2.1). $\square$

### Condition 6

This is immediate with $(\gamma , \sigma _0^2)=(1, \sigma ^2_{\scriptscriptstyle D}v)$ by Proposition 1.7. $\square$

It is worth noting however that we can also invoke Lemma 2.2 by checking its simpler hypotheses. The first hypothesis of Lemma 2.2 was verified for lattice trees with $d>8$ in [38, Theorem 1.5] as a consequence of the survival asymptotics proved therein and [22, Theorem 1.15] and [24, Proposition 2.4]. The second hypothesis of Lemma 2.2 is easily obtained from the identity

$$\begin{aligned} {\mathbb {E}}^s_n[X_t^{\scriptscriptstyle {(n)}}(1)^p]=\frac{C_s n}{n^p}\sum _{x_1,\dots ,x_p}\sum _{\begin{array}{c} T\in {\mathbb {T}}_L(o):\\ x_1,\dots , x_p \in T_{\lfloor nt \rfloor } \end{array}}W(T). \end{aligned}$$

This identity gives rise to the bound

$$\begin{aligned} \sup _{t\le t^*}{\mathbb {E}}^s_n[X_t^{\scriptscriptstyle {(n)}}(1)^p]\le \sup _{t\le t^*}\frac{C_{s,p}n}{n^p} \lfloor nt \rfloor ^{p-1}\le C_{s,p}{ t^*}^p, \end{aligned}$$

where the factor $\lfloor nt \rfloor ^{p-1}$ comes from the possible temporal locations of $p-1$ branch points in the minimal subtree connecting *o* to the points $x_1,\dots , x_p\in T_{\lfloor nt \rfloor }$ (see e.g. [22, (4.4)–(4.5)]).

In preparation for proving the remaining conditions, we introduce a bit of notation:

For any tree $T\in {\mathbb {T}}_L$ and any $x\in T$, let $R_x(T)$ denote the lattice tree consisting of *x* and the descendants of *x* in *T*, together with the edges in *T* connecting them. So in particular if $x\in T_n$, then

$$\begin{aligned} V(R_x(T))=\{y\in {\mathbb {Z}}^d: \exists m\ge n \text { s.t. }x=w_n(m,y)\}. \end{aligned}$$

Let $T_{\ngtr x}=(T{\setminus } R_x(T))\cup \{x\}$ denote the tree consisting of all vertices in *T* that are not descendants of *x*. It is connected, and hence a tree, since for any such vertex *y*, the path from *o* to *y* cannot contain any descendants of *x* or else *y* would also be a descendant. For any $B\subset {\mathbb {T}}_L$, let $B_x$ denote the event *B* shifted by *x* (i.e. for $T\ni o$, $T \in B \iff T+x \in B_x$, where $+$ is addition in ${\mathbb {Z}}^d$).

**Notation.** We will write ${\varvec{\omega }}_n=(\omega _0,\omega _1,\dots ,\omega _n)$ to denote an *n*-step random walk path, that is, a sequence of points $\omega _i\in {\mathbb {Z}}^d$ so that $\Vert \omega _i-\omega _{i-1}\Vert _\infty \le L$ for all $1\le i\le n$. We write ${\varvec{\omega }}_n:y\rightarrow z$ if, in addition, $\omega _0=y$ and $\omega _n=z$, in which case ${\varvec{\omega }}_n$ is a random walk path from *y* to *z*. If ${\varvec{\omega }}$ is an *n*-step random walk path we write ${\varvec{\omega }}\in {\mathbb {T}}_L(\omega _0)$ iff ${\varvec{\omega }}$ is also self-avoiding (i.e., $\omega _0,\dots ,\omega _n$ are distinct), where it is understood that the edge set is precisely the set of *n* edges $\{\{\omega _{i-1},\omega _i\}\}_{i=1}^n$.

If $(T_i)_{i \in I}$ are lattice trees, we define the union of these trees as the lattice subgraph with vertex set equal to the union of the vertex sets of the $T_i$, and edge set equal to the union of the edge sets of the $T_i$.

If ${\varvec{\omega }}_n$ is an *n*-step random walk path and $\mathbf {R}_n=(R_0,\dots ,R_n)$, where $R_i\in {\mathbb {T}}_L(\omega _i)$ for each $i=0,\dots , n$, then we write $\mathbf {R}_{_n}\ni {\varvec{\omega }}_n$.

### Remark 9.3

Here we describe a bijection between $T \in {\mathbb {T}}_L(o)$ such that $x\in T_n$ and collections $({\varvec{\omega }}_n,\mathbf {R}_n)$, where $\mathbf {R}_{_n}\ni {\varvec{\omega }}_n$, $\omega _0=o$ and $\omega _n=x$, and the $(R_i)_{i=0}^n$ are mutually avoiding (i.e. vertex disjoint, which implies that ${\varvec{\omega }}_n\in {\mathbb {T}}_L(o)$).

Firstly note that any lattice tree $T\in {\mathbb {T}}_L(o)$ such that $x\in T_n$ has a unique *n*-step random walk path ${\varvec{\omega }}_n:=w(n,x)\in {\mathbb {T}}_L(o)$ of vertices and edges in *T* from *o* to *x*. Define $R_i$ to be the connected component of $\omega _i$ in the tree after removing the edges of ${\varvec{\omega }}_n$ (but not the vertices) from *T*. Then trivially each $R_i\in {\mathbb {T}}_L(\omega _i)$, and the $(R_i)_{i=0}^n$ are mutually avoiding (i.e. vertex disjoint). Moreover *T* is the union of the trees ${\varvec{\omega }}_n$ and $(R_i)_{i=0}^n$.

On the other hand, given an *n*-step random walk path ${\varvec{\omega }}_n\in {\mathbb {T}}_L(o)$ from *o* to *x*, and $\mathbf {R}_n \ni {\varvec{\omega }}_n$, the (edge and vertex) union of these trees is a tree if (and only if) the $(R_i)_{i=0}^n$ are mutually avoiding.

It is immediate from Remark 9.3 (and the product form of *W*(*T*)) that the two-point function, ${\mathbb {P}}(x \in \mathcal {T}_n)$, can be written as

9.5 $$\begin{aligned} {\mathbb {P}}(x \in \mathcal {T}_n)&:=\rho ^{-1}\sum _{T \in {\mathbb {T}}_L(o)}W(T) \mathbb {1}_{\{x \in T_n\}} \nonumber \\&\, =\rho ^{-1}\sum _{ {\varvec{\omega }}_n: o \rightarrow x}W({\varvec{\omega }}_n)\sum _{\mathbf {R}_{_n}\ni {\varvec{\omega }}_n}\left( \prod _{i=0}^nW(R_i)\right) \mathbb {1}_{\{R_0,\dots , R_n \text { avoid each other}\}}. \end{aligned}$$

We henceforth write $W(\mathbf {R}_n):=\prod _{i=0}^nW(R_i)$ when $\mathbf {R}_n=(R_0,\dots , R_n)$.

Obviously we obtain an upper bound for (9.5) by replacing the indicator therein with a less restrictive one. This observation and generalisations of it will play a crucial role in our verification of the conditions for lattice trees.

Conditions 3 and 7 will be simple consequences of the following Lemma.

### Lemma 9.4

For all $A,B\subset {\mathbb {T}}_L$, and every $n\in {\mathbb {N}}$,

9.6 $$\begin{aligned} {\mathbb {P}}(x \in \mathcal {T}_n,\mathcal {T}_{\ngtr x}\in A, R_x(\mathcal {T})\in B_x)\le \rho {\mathbb {P}}(x \in \mathcal {T}_n,\mathcal {T}_{\ngtr x}\in A){\mathbb {P}}(\mathcal {T}\in B). \end{aligned}$$

### Proof

Using Remark 9.3 we see that the left hand side of (9.6) is equal to

9.7 $$\begin{aligned}&\frac{1}{\rho }\sum _{T \in {\mathbb {T}}_L}W(T) \mathbb {1}_{\{x\in T_n\}}\mathbb {1}_{\{T_{\ngtr x}\in A\}}\mathbb {1}_{\{R_x(T)\in B_x\}}\nonumber \\&\quad =\frac{1}{\rho }\sum _{{\varvec{\omega }}_n:o \rightarrow x}W({\varvec{\omega }}_n)\sum _{\mathbf {R}_{_n}\ni {\varvec{\omega }}_n}W(\mathbf {R}_n)\mathbb {1}_{\{R_0,\dots , R_n \text { avoid each other}\}}\nonumber \\&\quad \quad \quad \quad \quad \quad \quad \quad \quad \quad \quad \quad \quad \quad \quad \quad \quad \quad \quad \times \mathbb {1}_{\{\mathbf {R}_{n-1}\cup {\varvec{\omega }}_{n}\in A\}}\mathbb {1}_{\{R_n\in B_x\}}, \end{aligned}$$

where $\mathbf {R}_{n}\cup {\varvec{\omega }}_{n}=:T'$ is a lattice tree (containing *o*, and *x* at generation *n*) due to the indicator of avoidance, and $\mathbf {R}_{n-1}\cup {\varvec{\omega }}_{n}=(T'{\setminus } R_n)\cup \{x\}$ is a tree as well.

Now $R_n$ is a tree containing $x=\omega _n$, so by weakening the avoidance constraint this is at most

9.8 $$\begin{aligned}&\frac{1}{\rho }\sum _{{\varvec{\omega }}_n:o \rightarrow x}W({\varvec{\omega }}_n)\sum _{\mathbf {R}_{n}\ni {\varvec{\omega }}_n}W(\mathbf {R}_n)\mathbb {1}_{\{R_0,\dots , R_{n-1} \text { avoid each other and }x\}}\nonumber \\&\quad \quad \quad \quad \quad \quad \quad \quad \quad \quad \quad \quad \quad \quad \quad \quad \times \mathbb {1}_{\{\mathbf {R}_{n-1}\cup {\varvec{\omega }}_{n}\in A\}}\mathbb {1}_{\{R_n\in B_x\}}\nonumber \\&\quad =\frac{1}{\rho }\sum _{{\varvec{\omega }}_n:o \rightarrow x}W({\varvec{\omega }}_n)\sum _{\mathbf {R}_{n-1}\ni {\varvec{\omega }}_{n-1}}W(\mathbf {R}_{n-1})\mathbb {1}_{\{R_0,\dots , R_{n-1} \text { avoid each other and }x\}}\nonumber \\&\quad \quad \quad \quad \quad \quad \quad \quad \quad \quad \quad \quad \quad \quad \quad \quad \quad \quad \quad \quad \quad \quad \times \mathbb {1}_{\{\mathbf {R}_{n-1}\cup {\varvec{\omega }}_{n}\in A\}} \end{aligned}$$

9.9 $$\begin{aligned}&\qquad \times \sum _{R_n \in {\mathbb {T}}_L(x)}W(R_n)\mathbb {1}_{\{R_n\in B_x\}}, \end{aligned}$$

where we have used the fact that $\mathbb {1}_{\{\mathbf {R}_{n-1}\cup {\varvec{\omega }}_{n}\in A\}}$ does not depend on $R_n{\setminus } \{x\}$. Now note that (9.9) is equal to

$$\begin{aligned} \rho \frac{1}{\rho }\sum _{R \in {\mathbb {T}}_L(x)}W(R)\mathbb {1}_{\{R \in B_x\}}=\rho {\mathbb {P}}(\mathcal {T}+x\in B_x)=\rho {\mathbb {P}}(\mathcal {T}\in B). \end{aligned}$$

Next note that the weight of a lattice tree consisting of a single vertex $\{x\}$ is 1, so (9.8) is at most

9.10 $$\begin{aligned} \frac{1}{\rho }\sum _{{\varvec{\omega }}_n:o \rightarrow x}W({\varvec{\omega }}_n)\sum _{\mathbf {R}_{n}\ni {\varvec{\omega }}_{n}}W(\mathbf {R}_n)\mathbb {1}_{\{R_0,\dots , R_{n} \text { avoid each other}\}}\mathbb {1}_{\{\mathbf {R}_{n-1}\cup {\varvec{\omega }}_{n}\in A\}}, \end{aligned}$$

since (9.10) contains the case where $R_n=\{x\}$. But (9.10) is equal to

$$\begin{aligned} {\mathbb {P}}(x \in \mathcal {T}_n,\mathcal {T}_{\ngtr x}\in A), \end{aligned}$$

and the result follows. $\square$

### Condition 3

By (9.2), to verify Condition 3 for lattice trees, it is sufficient to show that there exists $c_{3}>0$ such that for all $n\in {\mathbb {Z}}_+,k\in {\mathbb {N}}$, any $T'\in {\mathbb {T}}_{\le n}$ such that ${\mathbb {P}}(\mathcal {T}_{\le n}=T')>0$ and any $x \in T'_n$,

9.11 $$\begin{aligned} {\mathbb {P}}(\exists z : (n,x) \overset{\varvec{a}}{\rightarrow }(n+k,z) \big | \mathcal {T}_{\le n}=T')\le \frac{c_{3}}{k}. \end{aligned}$$

Let *B* denote the set of lattice trees containing *o* that survive until at least generation *k*, so $B_x$ is the set of lattice trees rooted at *x* for which there is at least one vertex in the tree of tree distance *k* from *x*. Then

9.12 $$\begin{aligned} {\mathbb {P}}(\exists z : (n,x) \overset{\varvec{a}}{\rightarrow }(n+k,z) \big | \mathcal {T}_{\le n}=T')&=\frac{{\mathbb {P}}(\exists z : (n,x) \overset{\varvec{a}}{\rightarrow }(n+k,z) \, , \, \mathcal {T}_{\le n}=T')}{{\mathbb {P}}(\mathcal {T}_{\le n}=T')}\nonumber \\&=\frac{{\mathbb {P}}(R_x(\mathcal {T})\in B_x \, , \, \mathcal {T}_{\le n}=T')}{{\mathbb {P}}(\mathcal {T}_{\le n}=T')}. \end{aligned}$$

Note that for any $T\in {\mathbb {T}}_L$, if $x \in T_n$ then $T_{\le n}=(T_{\ngtr x})_{\le n}$. Therefore the numerator in (9.12) can be written as

$$\begin{aligned} {\mathbb {P}}\big (x \in \mathcal {T}_n, (\mathcal {T}_{\ngtr x})_{\le n}=T', R_x(\mathcal {T})\in B_x\big )\le \rho {\mathbb {P}}\big (x \in \mathcal {T}_n, (\mathcal {T}_{\ngtr x})_{\le n}=T'\big ){\mathbb {P}}(\mathcal {T}\in B\big ), \end{aligned}$$

where we have used Lemma 9.4. But (since $x \in T'_n$),

$$\begin{aligned} {\mathbb {P}}\big (x \in \mathcal {T}_n, (\mathcal {T}_{\ngtr x})_{\le n}=T'\big )={\mathbb {P}}(\mathcal {T}_{\le n}=T'), \end{aligned}$$

so for all $k\in {\mathbb {N}}$, (9.12) is bounded above by $\rho {\mathbb {P}}(\mathcal {T}\in B\big )=\rho \theta (k)\le c\rho /k$, by (1.22) and Condition 1. By (1.27) we have proved (9.11), as needed. $\square$

### Condition 7

Let $(R_x(\mathcal {T}))_m$ denote the set of vertices in the tree $R_x(\mathcal {T})$ of tree distance *m* from *x* (e.g. $(R_x(\mathcal {T}))_0=\{x\}$). By Lemma 2.3 we need to show that there exists $c_{7}>0$ such that for any $\ell \in {\mathbb {Z}}_+,m\in {\mathbb {N}}^{\ge 4}$, and $M>0$,

9.13 $$\begin{aligned}&{\mathbb {E}}\left[ \sum _{x \in \mathcal {T}_\ell }\mathbb {1}\Big (\exists y \in (R_x(\mathcal {T}))_m\, , \, \sum _{j=m+2}^{2m-1}|(R_x(\mathcal {T}))_j|\le M\Big )\right] \nonumber \\&\quad \le c_{7} {\mathbb {P}}\Big (S^{\scriptscriptstyle (1)}\ge m\, ,\, \sum _{j=m+2}^{2m-1} |\mathcal {T}_j|\le M\Big ). \end{aligned}$$

The left hand side can be written as

9.14 $$\begin{aligned}&\sum _{x\in {\mathbb {Z}}^d}{\mathbb {P}}\Bigg (x \in \mathcal {T}_\ell \, , \, \exists y \in (R_x(\mathcal {T}))_m\, , \,\sum _{j=m+2}^{2m-1} |(R_x(\mathcal {T}))_j|\le M\Bigg ). \end{aligned}$$

Let $B_x$ denote the set of lattice trees *T* rooted at *x* (i.e. the unique particle of generation 0 is *x*) that survive until time *m* such that the total number of particles of generation between $m+2$ and $2m-1$ is at most *M*, and let $B=B_o$. Then (9.14) is

$$\begin{aligned} \sum _{x\in {\mathbb {Z}}^d}{\mathbb {P}}\Big (x \in \mathcal {T}_\ell , R_x(\mathcal {T})\in B_x\Big ). \end{aligned}$$

Applying Lemma 9.4 this is at most

$$\begin{aligned} \rho \sum _{x\in {\mathbb {Z}}^d}{\mathbb {P}}(x \in \mathcal {T}_\ell ){\mathbb {P}}( \mathcal {T}\in B)=\rho {\mathbb {E}}\left[ \sum _{x \in \mathcal {T}_\ell }1\right] {\mathbb {P}}\Bigg (\exists y \in \mathcal {T}_m,\sum _{j=m+2}^{2m-1} |\mathcal {T}_j|\le M\Bigg ), \end{aligned}$$

which, by Condition 2, verifies (9.13) with $c_{7}=\rho c_{3}$. $\square$

For $T\in {\mathbb {T}}_L(o)$, $0\le m\le n$, and $x \in T_{n}$, let $x_m(T)=w_{m}(n,x)$ denote the unique ancestor of *x* in *T* of generation *m*. In preparation for verifying Condition 4 subject to Lemma 9.2 we prove the following Lemma.

### Lemma 9.5

If $c_{3}$ is the constant in Condition 2 for lattice trees, then for any $f:{\mathbb {Z}}^d \rightarrow {\mathbb {R}}_+$ such that $f(-x)=f(x)$ and any $m,n\in {\mathbb {Z}}_+$, s.t. $m\le n$,

$$\begin{aligned} {\mathbb {E}}\left[ \sum _{x \in \mathcal {T}_{n}}f(x-x_m(\mathcal {T}))\right] \le c_{3} \sum _{y\in {\mathbb {Z}}^d}f(y){\mathbb {P}}(y \in \mathcal {T}_{n-m}). \end{aligned}$$

### Proof

Assume without loss of generality that $m<n$. The left hand side is equal to

9.15 $$\begin{aligned}&\rho ^{-1}\sum _{x,y \in {\mathbb {Z}}^d}\sum _{T \in {\mathbb {T}}_L}W(T) \mathbb {1}_{\{x \in T_{n}\}}\mathbb {1}_{\{x_m(T)=y\}}f(x-y). \end{aligned}$$

Now every

*tree T rooted at o and containing x at tree distance n from o, such that the unique path in the tree from o to x passes through y at tree distance m from o*

is also

*a tree (with the same weight) rooted atxcontainingoat tree distancenfromosuch that the unique path in the tree fromxtoopasses throughyat tree distance*$n-m$*fromx*,

and vice versa. The above are actually the same tree, but since we are also specifying the root, we will refer to the latter as $T_x$.

Translating this tree by $-x$, we obtain a tree $T'=T_x-x$ (with the same weight as *T*) rooted at $o=x-x$, containing $x':=-x$ at tree distance *n* from *o* and such that the unique path in $T'$ from *o* to $x'$ passes through $y':=y-x$ at tree distance $n-m$ from *o*. Since $x-y=-y'$, and $f(-y')=f(y')$, (9.15) is equal to

$$\begin{aligned} \rho ^{-1}\sum _{x',y' \in {\mathbb {Z}}^d}\sum _{T' \in {\mathbb {T}}_L}W(T') \mathbb {1}_{\{x' \in T'_{n}\}}\mathbb {1}_{\{x'_{n-m}(T')=y'\}}f(y'). \end{aligned}$$

Now we can simply drop the $'$ to get that (9.15) is equal to

$$\begin{aligned} \rho ^{-1}\sum _{x,y \in {\mathbb {Z}}^d}\sum _{T \in {\mathbb {T}}_L}W(T) \mathbb {1}_{\{x \in T_{n}\}}\mathbb {1}_{\{x_{n-m}(T)=y\}}f(y). \end{aligned}$$

Now as in (9.7) we can write this as

9.16 $$\begin{aligned} \rho ^{-1}\sum _{x,y \in {\mathbb {Z}}^d}\sum _{{\varvec{\omega }}_n:o \overset{n-m}{\rightarrow }y \overset{m}{\rightarrow }x}W({\varvec{\omega }}_n)\sum _{\mathbf {R}_{n}\ni {\varvec{\omega }}_n}W(\mathbf {R}_n)\mathbb {1}_{\{(R_i)_{0\le i\le n} \text { avoid each other}\}}f(y), \end{aligned}$$

where the sum over ${\varvec{\omega }}_n$ is a sum over random walk paths of length *n* from *o* to *x* that are at *y* at time $n-m$.

Now since $R_{n-m}\ni y$, we have that

$$\begin{aligned}&\mathbb {1}_{\{(R_i)_{0\le i\le n} \text { avoid each other}\}}\\&\le \mathbb {1}_{\{(R_i)_{0\le i\le n-m} \text { avoid each other}\}}\mathbb {1}_{\{(R_i)_{n-m+1\le i\le n} \text { avoid each other and }y\}}. \end{aligned}$$

Thus, using the fact that also the weight of a tree containing a single vertex *y* is 1, we see that (9.16) is at most

$$\begin{aligned}&\rho ^{-1}\sum _{x,y \in {\mathbb {Z}}^d}\sum _{{\varvec{\omega }}^{\scriptscriptstyle (1)}_{n-m}:o \rightarrow y }\sum _{{\varvec{\omega }}^{\scriptscriptstyle (2)}_m:y \rightarrow x }W({\varvec{\omega }}^{\scriptscriptstyle (1)}_{n-m})W({\varvec{\omega }}^{\scriptscriptstyle (2)}_m)\\&\qquad \qquad \qquad \quad \quad \quad \quad \quad \quad \quad \times \sum _{\mathbf {R}^{\scriptscriptstyle (1)}_{n-m}\ni {\varvec{\omega }}_{n-m}^{\scriptscriptstyle (1)}}W(\mathbf {R}^{\scriptscriptstyle (1)}_{n-m})\mathbb {1}_{\{(R^{\scriptscriptstyle (2)}_i)_{0\le i\le n-m} \text { avoid each other}\}}f(y)\\&\quad \quad \quad \quad \quad \quad \quad \qquad \qquad \qquad \times \sum _{\mathbf {R}^{\scriptscriptstyle (2)}_{m}\ni {\varvec{\omega }}_m^{\scriptscriptstyle (2)}}W(\mathbf {R}^{\scriptscriptstyle (2)}_{m})\mathbb {1}_{\{(R^{\scriptscriptstyle (2)}_{i'})_{0\le i'\le m} \text { avoid each other}\}}. \end{aligned}$$

Collecting terms, this is equal to

$$\begin{aligned}&\rho ^{-1}\sum _{y \in {\mathbb {Z}}^d}\sum _{{\varvec{\omega }}^{\scriptscriptstyle (1)}_{n-m}:o \rightarrow y }W({\varvec{\omega }}^{\scriptscriptstyle (1)}_{n-m})\sum _{\mathbf {R}^{\scriptscriptstyle (1)}_{n-m}\ni {\varvec{\omega }}^{\scriptscriptstyle (1)}_{n-m}}W(\mathbf {R}^{\scriptscriptstyle (1)}_{n-m})\\&~~~~~~~~~~~~~~~~~~~~~~~~~~~~\qquad \quad \quad \quad \quad \quad \quad \qquad \qquad \times \mathbb {1}_{\{(R^{\scriptscriptstyle (1)}_i)_{0\le i\le n-m} \text { avoid each other}\}}f(y)\\&\quad \quad \times \left( \sum _{x \in {\mathbb {Z}}^d}\sum _{{\varvec{\omega }}^{\scriptscriptstyle (2)}_m:y \rightarrow x }W({\varvec{\omega }}^{\scriptscriptstyle (2)}_m)\sum _{\mathbf {R}^{\scriptscriptstyle (2)}_{m}\ni {\varvec{\omega }}^{\scriptscriptstyle (2)}_m}W(\mathbf {R}^{\scriptscriptstyle (2)}_{m})\mathbb {1}_{\{(R^{\scriptscriptstyle (2)}_{i'})_{0\le i\le m} \text { avoid each other}\}}\right) . \end{aligned}$$

Changing variables from *x* to $u=x-y$ in the last summation, we see the above is equal to

$$\begin{aligned} \sum _{y\in {\mathbb {Z}}^d}f(y){\mathbb {P}}(y \in \mathcal {T}_{n-m})\sum _{u \in {\mathbb {Z}}^d}{\mathbb {P}}(u\in {\mathcal {T}}_m)\le c_{3}\sum _{y\in {\mathbb {Z}}^d}f(y){\mathbb {P}}(y \in \mathcal {T}_{n-m}), \end{aligned}$$

by Condition 2 for lattice trees, as required. $\square$

### Condition 4

Use Lemma 9.5 with $f(x)=|x|^p$ ($p>4$), $n=\lfloor t\rfloor$, $m=\lfloor t-s\rfloor$, and apply the tree structure to see that the left side of (2.1) is at most

$$\begin{aligned} c_2\sum _{y\in {\mathbb {Z}}^d}|y|^p{\mathbb {P}}(y \in \mathcal {T}_{\lfloor t\rfloor -\lfloor t-s\rfloor })\le c(p,L)(s\vee 1)^{p/2}, \end{aligned}$$

the above inequality by Lemma 9.2. This verifies Condition 4. $\square$

We can now prove Theorem 6.

### Proof

Identical to the proof of Theorem 5. $\square$

## Verifying the conditions for the contact process

In this section we quickly verify all of our Conditions for the critical contact process with $d>4$, and hence establish Theorem 7.

Let ${\mathbb {P}}_\lambda$ denote a probability measure on a space under which we have a collection of independent Poisson point processes on ${\mathbb {R}}_+$, $\{\varLambda (x)(dt),\varLambda (x,y)(dt):x,y\in {\mathbb {Z}}^d\}$ where $\varLambda (x)$ has rate one and $\varLambda (x,y)$ has rate $\lambda D(x-y)$. Points of $\varLambda (x,y)$ are times where an infected site *y* will infect a non-infected site *x*, and points of $\varLambda (x)$ are times where an infected site *x* will recover. As for the voter model we may assume these point processes are mutually disjoint and do not contain 0. Let

$$\begin{aligned} {\mathcal {F}}^0_t=\sigma (\{\varLambda (x,y)([0,s]),\varLambda (x)([0,s]):s\le t, x,y\in {\mathbb {Z}}^d\}), \text { and }{\mathcal {F}}_t={\mathcal {F}}^0_{t+}. \end{aligned}$$

The construction below is a minor modification (to ensure right-continuity) of that described in Section 3 of [13]. For each point *s* in $\varLambda (x)$ we put a $\delta$ at (*s*, *x*), and for each point *t* in $\varLambda (x,y)$ we draw an arrow from (*t*, *y*) to (*t*, *x*). We write $(s,y)\rightarrow (t,x)$ iff $s=t$ and $x=y$ or $s<t$ and there is an oriented path from (*s*, *y*) to (*t*, *x*) which does not contain a $\delta$. The latter means that there are $s=s_0<s_1<\cdots s_k\le s_{k+1}=t$ ($k\in {\mathbb {Z}}_+$) and points $y=y_0,y_1,\dots , y_k,y_{k+1}=x$ in ${\mathbb {Z}}^d$ s.t.

10.1 $$\begin{aligned}&(i)\ \forall i=1,\dots ,k\text { there is an arrow from }(s_i,y_{i-1})\text { to }(s_i,y_i), \end{aligned}$$

10.2 $$\begin{aligned}&(ii)\ \forall i=0,\dots ,k, \ \{y_i\}\times (s_i,s_{i+1}]\text { contains no }\delta . \end{aligned}$$

It follows (see Section 3 of [13]) that

10.3 $$\begin{aligned} \xi _t(x)=1\ \text {iff }\exists y\ \text {s.t. }\xi _0(y)=1\text { and }(0,y)\rightarrow (t,x) \end{aligned}$$

defines a (cadlag) $({\mathcal {F}}_t)$-adapted contact process with initial state $\xi _0$ under ${\mathbb {P}}_\lambda$. Let $P_{\lambda ,\xi _0}$ denote the law of the process $(\xi _t)_{t\ge 0}$ with initial condition $\xi _0$. Define ${\mathcal {T}}_t=\{x:\xi _t(x)=1\}$. By Theorem 1.1 of [41] (see also (1.16) of that reference) there is a $\lambda _c=1+O(L^{-2d})>0$ as $L\rightarrow \infty$ such that $\lim _{t\rightarrow \infty }P_{\lambda ,\delta _o}({\mathcal {T}}_t\ne \emptyset )=0$ iff $\lambda \le \lambda _c$. Henceforth we will consider only the critical contact process and set $P_{\xi _0}=P_{\lambda _c,\xi _0}$ and ${\mathbb {P}}={\mathbb {P}}_{\lambda _c}$. Note that allowing $s_k=s_{k+1}=t$ and taking $y_k=y_{k+1}=x$ allows us to include arrows arising at time *t* and helps ensure right continuity. More generally by translation invariance and independence properties of our Poisson point processes, if for $s\ge 0$ and $\xi '\in \{0,1\}^{{\mathbb {Z}}^d}$ we define

$$\begin{aligned} \xi ^{s, \xi '}_t(x)=1\text { iff }\exists y\text { s.t. }\xi '(y)=1\text { and }(s,y)\rightarrow (s+t,x), \end{aligned}$$

then

10.4 $$\begin{aligned} \text {conditional on }{\mathcal {F}}_s,\ (\xi ^{s,\xi '}_t)_{t\ge 0}\text { has law }P_{\xi '}. \end{aligned}$$

As was done for the voter model in verifying Condition 5, it is easy to define a supercritical binary branching random walk $\hat{\xi }$ on the same space, starting at $\xi _0$, s.t.

10.5 $$\begin{aligned} \xi _t(x)\le \hat{\xi }_t(x)\text { for all }t\ge 0, x\in {\mathbb {Z}}^d. \end{aligned}$$

One essentially ignores recoveries; see (2.8) in [14] for a similar domination. In particular this shows that if $\mathcal {T}_0$ is almost surely finite then ${\mathcal {T}}_t$ is a cadlag (as for the voter model) $\mathcal {K}$-valued process taking values in the finite subsets of ${\mathbb {Z}}^d$. Setting $\mathcal {T}_0=\{o\}$ (equivalently $\xi _0=\delta _o$) we see that (1.3) holds under ${\mathbb {P}}$. It is easy to check that

10.6 $$\begin{aligned} \xi _t(x)=\xi ^{s,\xi _s}_{t-s}(x) \text { for all }0\le s\le t, x\in {\mathbb {Z}}^d. \end{aligned}$$

This and (10.4) readily give the $({\mathcal {F}}_t)$-Markov property:

### Lemma 10.1

For any $0\le s<t$ and Borel *A* in $\{0,1\}^{{\mathbb {Z}}_d}$,

$$\begin{aligned} {\mathbb {P}}(\xi _t\in A|{\mathcal {F}}_s)=P_{\xi _s}(\xi _{t-s}\in A). \end{aligned}$$

We define $(s,y)\overset{\varvec{a}}{\rightarrow }(t,x)$ iff $(0,o)\rightarrow (s,y)\rightarrow (t,x)$. It is straightforward to check this defines an ancestral relation for the CP. Only AR(iii) takes a bit of work and it is established using Remark 1.1(2).

Condition 1 holds by Theorem 1.5 of [38] with $m(t)=A^2V(t\wedge 1)$ and $s_D=2A$, where $A,V>0$ are the *L*-dependent constants introduced in [38].

### Lemma 10.2

Let $f:{\mathbb {Z}}^d \rightarrow {\mathbb {R}}_+$. Then for the critical contact process (sufficiently spread out, in dimensions $d>4$), and $0\le s<t$,

$$\begin{aligned} {\mathbb {E}}\left[ \sum _{x\in {\mathcal {T}}_t}\sum _{y\in {\mathcal {T}}_{s}}\mathbb {1}((s,y)\rightarrow (t,x))f(x-y)\right]&={\mathbb {E}}[\mathcal {T}_{s}] \sum _{z\in {\mathbb {Z}}^d}f(z){\mathbb {P}}(z \in \mathcal {T}_{t-s})\\&\le c_2 \sum _{z\in {\mathbb {Z}}^d}f(z){\mathbb {P}}(z \in \mathcal {T}_{t-s}). \end{aligned}$$

### Proof

This follows just as for the corresponding result for OP (Lemma 8.2). $\square$

### Proof of Theorem 7

To apply Corollary 1, Theorem 2 and Theorem 3 to derive these results it remains to check Conditions 2-7 for the claimed parameter values. Condition 2 is immediate from (1.9) in [42] with $k=0$. Condition 3 follows easily from (10.4) and Condition 1. Condition 4, for any $p>4$, is a consequence of Theorem 1.2 of [33] and Lemma 10.2. Condition 5 for any $\kappa >4$ is established by making minor modifications in the corresponding argument for the voter model using the domination (10.5). Condition 7 follows from a straightforward calculation using Lemma 10.1, (10.4) and Condition 2. Again it is similar to the corresponding verification for the voter model.

To check Condition 6 we verify the hypotheses of Lemma 2.2 with $(\gamma , \sigma _0^2)=(1, \sigma ^2_{\scriptscriptstyle D}v)$. The convergence of the finite-dimensional distributions follows from convergence of the Fourier transforms of the *r*-point functions in Theorem 1.2 of [42], Proposition 2.5 of [24], and the already checked survival asymptotics in Condition 1 (as for OP). The higher moment bound follows from Theorem 1.2(i) in [42] with $\mathbf {k}=\mathbf {0}$. $\square$
